# Supplementary material for: Assessing the impact of obesity interventions in the early years: a systematic review of UK-based studies
Source: BMJ Open. 2024 May 13;14(5):e076479. doi: 10.1136/bmjopen-2023-076479 (PMC11097867; doi:10.1136/bmjopen-2023-076479)

Supplementary Material

Appendix 1 – Search terms and search strategy

The following databases were searched in November 2021. The searches were updated in March 2023.

1. Pubmed/Medline
2. Cochrane Library Web
3. CINAHL
4. Scopus
5. PsycINFO
6. Web of Science
7. Social Policy and Practice
8. Health Management Information Consortium (HMIC)
9. Trials Register of Promoting Health Interventions (TRoPHI)

The indicative search terms that were used are presented on the table below:

Table S1: Search terms

**Concept 1**<br><br>**Participants**	**Concept 2**<br><br>**Exposure**	**Concept 3**<br><br>**Outcomes**
Subject headings:<br><br>“Child, preschool” OR<br><br>“Schools, nursery” OR<br><br>“Child day care centers”	(("diet" OR "food" OR "beverages" OR "food quality" OR "food preferences" OR "feeding behavior*")<br><br>OR<br><br>("exercise" OR "sedentary behavior" " OR "physical fitness"))<br><br>AND<br><br>("weight reduction programs" OR "health education" OR "health promotion" OR "primary prevention" OR "pilot projects" OR "feasibility studies" OR "program evaluation")	("obesity" OR "body mass index" OR "body weight" OR "body size" OR "body composition" OR "body weight changes" OR

Keywords<br><br>"child preschool" OR "preschool*" OR "preschool*" OR "pre school*" OR "kindergarten" OR "kindergarden" OR "prekinder*" OR "pre kinder*" OR "pre kinder*" OR "headstart" OR "head start" OR "sure start" OR "surestart" OR "family child care home*" OR "family childcare home*" OR "infant*" OR "toddler*" OR "childcare" OR "child-care" OR "child-care" OR "early learning center*" OR "early learning centre*" OR "early child*" OR "early care and education" OR ("child*" AND ("day-care" OR "day-care" OR "daycare")) OR "playgroup*" OR "play group*" OR "schools nursery" OR (("center" OR "center s" OR "centers"OR "centre" OR "centre s" OR "centres") AND "child, preschool") OR "child day care centers" OR "child day care centers")	(("diet" OR "dietary" OR "food" OR "beverages"OR "snack*" OR "fruit*" OR "vegetable*" OR OR "food quality" OR "drink*" OR "juice*" OR "nutrition*" OR "food habit*" OR "food choice*" OR "overeat*" OR "over eat*" OR "weight control" OR "weight management" OR "menu planning" OR "menu choice*" OR "meal choice*" OR "meal*" OR "meal planning" OR ("food" AND "consum*") OR "food preferences" OR "feeding behavior*" OR "feeding behaviour*" OR "energy density" OR "lifestyle behaviour" OR "eating behaviour" OR "healthy eating" OR "health promotion" OR "eating habit*")<br><br>OR<br><br>("exercise" OR "sedentary behaviour OR "physical fitness" OR "movement" OR "playtime"OR "play*" OR "sport*"))<br><br>AND<br><br>("intervention*" OR "program*" OR "programme*" OR "project*" OR "therapeutics" OR "scheme" OR "initiative*" OR "strateg*" OR "service*" OR "primary prevention" OR "health education" OR "health promotion" OR "primary prevention" OR "treat*" OR "therapy*" OR "pilot projects" OR "feasibility studies" OR "program evaluation")	"overweight")("obesity" OR "obes*" OR "body mass index" OR "BMI" OR "bmi z score" OR "bmi z score" OR "body mass index z-score" OR "body mass index z-score" OR "bmi percentile*" OR "bmi centile*" OR "body mass index percentile*" OR "body mass index centile*" OR "adipos*" OR "overweight" OR "healthy weight" OR "weight loss" OR "weight gain")

Search strategy in all databases:

PubMed

#1<br><br>Child/setting terms	("child, preschool"[MeSH Terms] OR "child preschool"[Title/Abstract] OR "preschool*"[Title/Abstract] OR "preschool*"[Title/Abstract] OR "pre school*"[Title/Abstract] OR "kindergarten"[Title/Abstract] OR "kindergarden"[Title/Abstract] OR "prekinder*"[Title/Abstract] OR "pre kinder*"[Title/Abstract] OR "pre kinder*"[Title/Abstract] OR "headstart"[Title/Abstract] OR "head start"[Title/Abstract] OR "sure start"[Title/Abstract] OR "surestart"[Title/Abstract] OR "family child care home*"[Title/Abstract] OR "family childcare home*"[Title/Abstract] OR "infant*"[Title/Abstract] OR "toddler*"[Title/Abstract] OR "childcare"[Title/Abstract] OR "child-care"[Title/Abstract] OR "child-care"[Title/Abstract] OR "early learning center*"[Title/Abstract] OR "early learning centre*"[Title/Abstract] OR "early child*"[Title/Abstract] OR "early care and education"[Title/Abstract] OR ("child*"[Title/Abstract] AND ("day-care"[Title/Abstract] OR "day-care"[Title/Abstract] OR "daycare"[Title/Abstract])) OR "playgroup*"[Title/Abstract] OR "play group*"[Title/Abstract] OR "schools, nursery"[MeSH Terms] OR "schools nursery"[Title/Abstract] OR (("center"[Title/Abstract] OR "center s"[Title/Abstract] OR "centers"[Title/Abstract] OR "centre"[Title/Abstract] OR "centre s"[Title/Abstract] OR "centres"[Title/Abstract]) AND "child, preschool"[MeSH Terms]) OR "child day care centers"[MeSH Terms] OR "child day care centers"[Title/Abstract])
#2<br><br>Diet terms	("diet"[MeSH Terms] OR "diet"[Title/Abstract] OR "dietary"[Title/Abstract] OR "food"[MeSH Terms] OR "food"[Title/Abstract] OR "beverages"[MeSH Terms] OR "beverages"[Title/Abstract] OR "snack*"[Title/Abstract] OR "fruit*"[Title/Abstract] OR "vegetable*"[Title/Abstract] OR "food quality"[MeSH Terms] OR "food quality"[Title/Abstract] OR "drink*"[Title/Abstract] OR "juice*"[Title/Abstract] OR "nutrition*"[Title/Abstract] OR "food habit*"[Title/Abstract] OR "food choice*"[Title/Abstract] OR "overeat*"[Title/Abstract] OR "over eat*"[Title/Abstract] OR "weight control"[Title/Abstract] OR "weight management"[Title/Abstract] OR "menu planning"[Title/Abstract] OR "menu choice*"[Title/Abstract] OR "meal choice*"[Title/Abstract] OR "meal*"[Title/Abstract] OR "meal planning"[Title/Abstract] OR ("food"[Title/Abstract] AND "consum*"[Title/Abstract]) OR "food preferences"[MeSH Terms] OR "food preferences"[Title/Abstract] OR "feeding behavior*"[MeSH Terms] OR "feeding behavior*"[Title/Abstract] OR "feeding behaviour*"[Title/Abstract] OR "energy density"[Title/Abstract] OR "lifestyle behaviour"[Title/Abstract] OR "eating behaviour"[Title/Abstract] OR "healthy eating"[Title/Abstract] OR "health promotion"[Title/Abstract] OR "eating habit*"[Title/Abstract])
#3<br><br>**PA terms**	("exercise"[MeSH Terms] OR "exercise"[Title/Abstract] OR "sedentary behavior"[MeSH Terms] OR "sedentary behavior"[Title/Abstract] OR "sedentary behaviour"[Title/Abstract] OR "physical fitness"[MeSH Terms] OR "physical fitness"[Title/Abstract] OR "movement"[Title/Abstract] OR "playtime"[Title/Abstract] OR "play*"[Title/Abstract] OR "sport*"[Title/Abstract])
#4<br><br>Intervention terms	("intervention*"[Title/Abstract] OR "weight reduction programs"[MeSH Terms] OR "program*"[Title/Abstract] OR "programme*"[Title/Abstract] OR "project*"[Title/Abstract] OR "therapeutics"[Title/Abstract] OR "scheme"[Title/Abstract] OR "initiatives"[Title/Abstract] OR "strateg*"[Title/Abstract] OR "service*"[Title/Abstract] OR "pilot projects"[MeSH Terms] OR "pilot projects"[Title/Abstract] OR "feasibility studies"[MeSH Terms] OR "feasibility studies"[Title/Abstract] OR "program evaluation"[MeSH Terms] OR "program evaluation"[Title/Abstract] OR "health education"[MeSH Terms] OR "health education"[Title/Abstract] OR "health promotion"[MeSH Terms] OR "health promotion"[Title/Abstract] OR "primary prevention"[Title/Abstract] OR "treat*"[Title/Abstract] OR "random*"[Title/Abstract] OR "cluster random*"[Title/Abstract] OR "trial*"[Title/Abstract] OR "evaluat*"[Title/Abstract] OR "effective*"[Title/Abstract] OR "mixed methods"[Title/Abstract] OR "impact*"[Title/Abstract])
#5	("intervention*"[Title/Abstract] OR "weight reduction programs"[MeSH Terms] OR "program*"[Title/Abstract] OR "programme*"[Title/Abstract] OR "project*"[Title/Abstract] OR "therapeutics"[Title/Abstract] OR "scheme"[Title/Abstract] OR "initiative*"[Title/Abstract] OR "strateg*"[Title/Abstract] OR "service*"[Title/Abstract] OR "health education"[MeSH Terms] OR "health promotion"[MeSH Terms] OR "primary prevention"[MeSH Terms] OR "primary prevention"[Title/Abstract] OR "health education"[Title/Abstract] OR "health

	promotion"[Title/Abstract] OR "primary prevention"[Title/Abstract] OR "treat*"[Title/Abstract] OR "therapy*"[Title/Abstract] OR "pilot projects"[MeSH Terms] OR "pilot projects"[Title/Abstract] OR "feasibility studies"[MeSH Terms] OR "feasibility studies"[Title/Abstract] OR "program evaluation"[MeSH Terms] OR "program evaluation"[Title/Abstract])
#6	("random*"[Title/Abstract] OR "cluster random*"[Title/Abstract] OR "trial*"[Title/Abstract] OR "evaluat*"[Title/Abstract] OR "effective*"[Title/Abstract] OR "mixed methods"[Title/Abstract] OR "impact*"[Title/Abstract] OR "non randomized controlled trial*"[Title/Abstract] OR "Pragmatic Clinical Trial"[Title/Abstract] OR "controlled clinical trial*"[Title/Abstract] OR "randomized controlled trial*"[Title/Abstract] OR "randomized"[Title/Abstract] OR "randomised"[Title/Abstract] OR "clinical trial*"[MeSH Terms] OR "clinical trial*"[Title/Abstract] OR "randomly"[Title/Abstract] OR "controlled study"[Title/Abstract] OR "controlled trial*"[Title/Abstract] OR "control group"[Title/Abstract] OR "intervention group"[Title/Abstract] OR "experimental group"[Title/Abstract] OR "comparison group"[Title/Abstract] OR "control school"[Title/Abstract] OR "intervention school"[Title/Abstract] OR "control community"[Title/Abstract] OR "intervention community"[Title/Abstract] OR "intervention condition"[Title/Abstract] OR "control condition"[Title/Abstract] OR "treatment group"[Title/Abstract] OR "control participant"[Title/Abstract] OR "quasi experimental design"[Title/Abstract] OR "experimental condition"[Title/Abstract] OR "treatment condition"[Title/Abstract] OR "pilot projects"[MeSH Terms] OR "feasibility studies"[MeSH Terms] OR "program evaluation"[MeSH Terms] OR "pre test"[Title/Abstract] OR "post test"[Title/Abstract] OR "quasi experiment*"[Title/Abstract] OR "blinded"[Title/Abstract] OR "double bind"[Title/Abstract] OR "fidelity"[Title/Abstract] OR "reach"[Title/Abstract] OR "uptake"[Title/Abstract] OR "implementation"[Title/Abstract] OR "intervention effect*"[Title/Abstract] OR "program effect*"[Title/Abstract] OR "programme effect*"[Title/Abstract] OR "proof of concept*"[Title/Abstract] OR "outcome study"[Title/Abstract])
#7	Search: #5 AND #6
#8	Search: #2 OR #3
#9	Search: #8 AND (#4 OR #7)
**#10**	("obesity"[MeSH Terms] OR "obesity"[Title/Abstract] OR "obes*"[Title/Abstract] OR "body mass index"[MeSH Terms] OR "body mass index"[Title/Abstract] OR "BMI"[Title/Abstract] OR "bmi z score"[Title/Abstract] OR "bmi z score"[Title/Abstract] OR "body mass index z-score"[Title/Abstract] OR "body mass index z-score"[Title/Abstract] OR "bmi percentile*"[Title/Abstract] OR "bmi centile*"[Title/Abstract] OR "body mass index percentile*"[Title/Abstract] OR "body mass index centile*"[Title/Abstract] OR "body weight"[MeSH Terms] OR "body size"[MeSH Terms] OR "adipos*"[Title/Abstract] OR "body composition"[MeSH Terms] OR "body weight changes"[MeSH Terms] OR "overweight"[MeSH Terms] OR "overweight"[Title/Abstract] OR "healthy weight"[Title/Abstract] OR "weight loss"[Title/Abstract] OR "weight gain"[Title/Abstract])
**#11**	Search: #1 AND #9 AND #10

CINAHL

#1<br>Population<br>AB	AB (preschool* OR pre-school* OR "pre-school*" OR kindergar?en OR prekinder* OR pre-Kinder* OR "pre Kinder*" OR headstart OR "head start" OR "surestart" OR "sure start" OR "family child care home" OR "family childcare home" OR "infant*" OR "toddler*" OR "child care" OR "child-care" OR childcare OR "early learning centre" OR "early child*" OR "early care and education" OR (child* AND "day care" OR "daycare" OR "day-care" OR playgroup* OR "nurser*" OR child day care centre*)) **OR** TI ((preschool* OR pre-school* OR "pre-school*" OR kindergar?en OR prekinder* OR pre-Kinder* OR "pre Kinder*" OR headstart OR "head start" OR "surestart" OR "sure start" OR "family child care home" OR "family childcare home" OR "infant*" OR "toddler*" OR "child care" OR "child-care" OR childcare OR "early learning centre" OR "early child*" OR "early care and education" OR (child* AND "day care" OR "daycare" OR "day-care" OR playgroup* OR "nurser*" OR child day care centre*))
#2<br>Diet<br>AB	AB (diet* OR dietary OR food* OR snack* OR fruit* OR drink* OR beverage* OR juice* OR vegetable* OR meal* OR nutrition* OR intake* OR consum* OR "food quality" OR "food preference*" OR "energy density" OR "lifestyle behavio#r" OR "eating behavio#r" OR "healthy eating" OR "feeding behavio#r" OR "health promotion" OR "eating habit*" OR "food habit*" OR "food choice*" OR "overeat*" OR "over eat*" OR "weight control" OR "weight management" OR "menu planning" OR "menu choice" OR "meal choice*" OR "meal*" OR "meal planning" OR (food* AND consum*) OR "eating habit*") **OR** TI (diet* OR dietary OR food* OR snack* OR fruit* OR drink* OR beverage* OR juice* OR vegetable* OR meal* OR nutrition* OR intake* OR consum* OR "food quality" OR "food preference*" OR "energy density" OR "lifestyle behavio#r" OR "eating behavio#r" OR "healthy eating" OR "feeding behavio#r" OR "health promotion" OR "eating habit*" OR "food habit*" OR "food choice*" OR "overeat*" OR "over eat*" OR "weight control" OR "weight management" OR "menu planning" OR "menu choice" OR "meal choice*" OR "meal*" OR "meal planning" OR (food* AND consum*) OR "eating habit*")
#3<br>PA<br>AB	AB ("physical* activ*" OR playtime OR sedentary OR exercise* OR play* OR "physical fitness" OR "movement" OR "playtime" OR "sport*") **OR** TI ("physical* activ*" OR playtime OR sedentary OR exercise* OR play* OR "physical fitness" OR "movement" OR "playtime" OR "sport*")
#4<br>Interventions<br>AB	AB ("intervention*" OR "weight reduction program*" "program*" OR "programme*" OR "project*" OR "therapeutics" OR "scheme" OR "initiative*" OR "strateg*" OR "life style" OR "service*" OR "pilot project*" OR "feasibility stud*" OR "program evaluation" OR "health education*" OR "health promotion" OR "primary prevention" OR "treat*" OR "random*" OR "cluster random*" OR "trial*" OR "evaluat*" OR "effective*" OR "mixed methods" OR "impact*") **OR** TI ("intervention*" OR "weight reduction program*" "program*" OR "programme*" OR "project*" OR "therapeutics" OR "scheme" OR "initiative*" OR "strateg*" OR "life style" OR "service*" OR "pilot project*" OR "feasibility stud*" OR "program evaluation" OR "health education*" OR "health promotion" OR "primary prevention" OR "treat*" OR "random*" OR "cluster random*" OR "trial*" OR "evaluat*" OR "effective*" OR "mixed methods" OR "impact*")
#5<br>Intervention terms	AB ("intervention*" OR "weight reduction programs" OR "program*" OR "programme*" OR "project*" OR "therapeutics" OR "scheme" OR "initiative*" OR "strateg*" OR "service*" OR "health education" OR "health promotion" OR "primary prevention" OR "health education" OR "health promotion" OR "primary prevention" or "treat*" OR therapy* or "pilot projects" OR "feasibility studies" OR "program evaluation") **OR** TI ("intervention*" OR "weight reduction programs" OR "program*" OR "programme*" OR "project*" OR "therapeutics" OR "scheme" OR "initiative*" OR "strateg*" OR "service*" OR "health education" OR "health promotion" OR "primary prevention" OR "health education" OR "health promotion" OR "primary prevention" or "treat*" OR therapy* or "pilot projects" OR "feasibility studies" OR "program evaluation")
#6<br>Evaluati	AB ("random*" OR "cluster random*" OR "trial*" OR "evaluat*" OR "effective*" OR "mixed methods" OR "impact*" OR "Non-Randomized Controlled Trial*" OR "Pragmatic Clinical Trial" OR "controlled Clinical Trial*" OR "randomized controlled trial*" OR "randomized" OR "randomised" OR "clinical trial*" OR "randomly" OR "controlled study" OR "controlled trial*" OR "control group" OR "intervention group" OR "experimental group" OR "comparison group" OR "control school" OR "intervention school" OR "control community" OR "intervention community" OR "intervention condition" OR "control condition" OR "treatment group" OR "control participant" OR "quasi experimental design" OR

on ter ms	"experimental condition" OR "treatment condition" OR "pilot projects" OR "feasibility studies" OR "program evaluation" OR "pre test" OR "post test" OR "quasi experiment*" OR blinded OR "double bind" OR fidelity OR reach OR uptake OR implementation OR "intervention effect*" OR "program effect*" OR "programme effect*" OR "proof of concept*" OR "outcome study") **OR** TI ("random*" OR "cluster random*" OR "trial*" OR "evaluat*" OR "effective*" OR "mixed methods" OR "impact*" OR "Non-Randomized Controlled Trial*" OR "Pragmatic Clinical Trial" OR "controlled Clinical Trial*" OR "randomized controlled trial*" OR “randomized” OR “randomised” OR "clinical trial*" OR “randomly” OR "controlled study" OR "controlled trial*" OR "control group" OR "intervention group" OR "experimental group" OR "comparison group" OR "control school" OR "intervention school" OR "control community" OR "intervention community" OR "intervention condition" OR "control condition" OR "treatment group" OR "control participant" OR "quasi experimental design" OR "experimental condition" OR "treatment condition" OR "pilot projects" OR "feasibility studies" OR "program evaluation" OR "pre test" OR "post test" OR "quasi experiment*" OR blinded OR "double bind" OR fidelity OR reach OR uptake OR implementation OR "intervention effect*" OR "program effect*" OR "programme effect*" OR "proof of concept*" OR "outcome study")
#7	S5 AND S6
#8	S2 OR S3
#9	S4 OR S7
#10	S8 AND S9<br><br>**Limiters** - Age Groups: Infant, 1-23 months, Child, Preschool 2-5 years
#11	AB ("obesity” OR “obes*” OR "body mass index" OR "BMI" OR "bmi z score" OR "bmi z score" OR "body mass index z-score" OR "body mass index z-score" OR "bmi percentile*" OR "bmi centile*" OR "body mass index percentile*" OR "body mass index centile*" OR "body weight" OR "body size" OR "weight*" OR "waist" OR "adipos*" OR "body composition" OR "overweight" OR “body weight changes” OR “healthy weight” OR “weight loss” OR “weight gain”) **OR** TI ("obesity” OR “obes*” OR "body mass index" OR "BMI" OR "bmi z score" OR "bmi z score" OR "body mass index z-score" OR "body mass index z-score" OR "bmi percentile*" OR "bmi centile*" OR "body mass index percentile*" OR "body mass index centile*" OR "body weight" OR "body size" OR "weight*" OR "waist" OR "adipos*" OR "body composition" OR "overweight" OR “body weight changes” OR “healthy weight” OR “weight loss” OR “weight gain”)<br><br>**Limiters** - Age Groups: Infant, 1-23 months, Child, Preschool 2-5 years
#12	#1 AND #10 AND #11<br><br>**Limiters** - Publication Year: 2011-2023

Cochrane

#1<br>Population<br>TI-AB-KW	(preschool* OR pre-school* OR "pre-school*" OR kindergar?en OR prekinder* OR pre-Kinder* OR "pre Kinder*" OR headstart OR "head start" OR "surestart" OR "sure start" OR "family child care home" OR "family childcare home" OR "infant*" OR "toddler*" OR "child care" OR "child-care" OR childcare OR "early learning centre" OR "early child*" OR "early care and education" OR (child* AND "day care" OR "daycare" OR "day-care" OR playgroup* OR "nurser*" OR child day care centre*))
#2<br>Diet<br>TI-AB-KW	(diet* OR dietary OR food* OR snack* OR fruit* OR drink* OR beverage* OR juice* OR vegetable* OR meal* OR nutrition* OR intake* OR consum* OR "food quality" OR "food preference*" OR "energy density" OR "lifestyle behavio*r" OR "eating behavio*r" OR "healthy eating" OR "feeding behavio*r" OR "health promotion" OR "eating habit*" OR "food habit*" OR "food choice*" OR "overeat*" OR "over eat*" OR "weight control" OR "weight management" OR "menu planning" OR "menu choice*" OR "meal choice*" OR "meal*" OR "meal planning" OR (food* AND consum*) OR "eating habit*")
#3<br>PA<br>TI-AB-KW	("physical* activ*" OR sport OR sports OR exercis* OR playtime OR sedentary OR exercise* OR play* OR "physical fitness" OR "movement" OR "playtime")
#4<br>Outcome terms<br>TI-AB-KW	(obesity OR obes* OR "body mass index" OR BMI OR "bmi z score" OR "bmi z score" OR "body mass index z-score" OR "body mass index z-score" OR "bmi percentile*" OR "bmi centile*" OR "body mass index percentile*" OR "body mass index centile*" OR "body weight" OR "body size" OR weight* OR waist OR "adipos*" OR "body composition" OR overweight OR "body weight changes" OR "healthy weight" OR "weight loss" OR "weight gain")
#5	#1 AND #6 AND #7
#6	("intervention*" OR "weight reduction programs" OR "program*" OR "programme*" OR "project*" OR "therapeutics" OR "scheme" OR "initiative*" OR "strateg*" OR "service*" OR "health education" OR "health promotion" OR "primary prevention" OR "health education" OR "health promotion" OR "primary prevention" or "treat*" OR therapy* or "pilot projects" OR "feasibility studies" OR "program evaluation")
#7	#5 AND #6

Web of Science

#1<br>Population<br>TS	TS=(preschool* OR pre-school* OR "pre-school*" OR kindergar$en OR prekinder* OR pre-Kinder* OR "pre Kinder*" OR headstart OR "head start" OR "surestart" OR "sure start" OR "family child care home" OR "family childcare home" OR "infant*" OR "toddler*" OR "child care" OR "child-care" OR childcare OR "early learning centre" OR "early child*" OR "early care and education" OR (child* AND "day care" OR "daycare" OR "day-care" OR playgroup* OR "nurser*" OR "child day care centre*"))
#2<br>Diet<br>TS	TS=((diet* OR dietary OR food* OR snack* OR fruit* OR drink* OR beverage* OR juice* OR vegetable* OR meal* OR nutrition* OR intake* OR consum* OR "food quality" OR "food preference*" OR "energy density" OR "lifestyle behavio$r" OR "eating behavio$r" OR "healthy eating" OR "feeding behavio$r" OR "health promotion" OR "eating habit*" OR "food habit*" OR "food choice*" OR "overeat*" OR "over eat*" OR "weight control" OR "menu planning" OR "menu choice*" OR "meal choice*" OR "meal*" OR "meal planning" OR (food* AND consum*) OR "eating habit*"))
#3<br>PA<br>TS	TS=(("physical* activ*" OR playtime OR sedentary OR exercise* OR play* OR "physical fitness" OR "movement" OR "playtime" OR sport*))
#4<br>Interventions<br>TS	TS=(("intervention*" OR "weight reduction program*" "program*" OR "programme*" OR "project*" OR "therapeutics" OR "scheme" OR "initiative*" OR "strateg*" OR "life style" OR "service*" OR "pilot project*" OR "feasibility stud*" OR "program evaluation" OR "health education*" OR "health promotion" OR "primary prevention" OR "treat*" OR "random*" OR "cluster random*" OR "trial*" OR "evaluat*" OR "effective*" OR "mixed methods" OR "impact*"))
#5	TS=(("intervention*" OR "weight reduction programs" OR "program*" OR "programme*" OR "project*" OR "therapeutics" OR "scheme" OR "initiative*" OR "strateg*" OR "service*" OR "health education" OR "health promotion" OR "primary prevention" OR "health education" OR "health promotion" OR "primary prevention" or "treat*" OR therapy* or "pilot projects" OR "feasibility studies" OR "program evaluation"))
#6	TS=(("random*" OR "cluster random*" OR "trial*" OR "evaluat*" OR "effective*" OR "mixed methods" OR "impact*" OR "Non-Randomized Controlled Trial*" OR "Pragmatic Clinical Trial" OR "controlled Clinical Trial*" OR "randomized controlled trial*" OR "randomized" OR "randomised" OR "clinical trial*" OR "randomly" OR "controlled study" OR "controlled trial*" OR "control group" OR "intervention group" OR "experimental group" OR "comparison group" OR "control school" OR "intervention school" OR "control community" OR "intervention community" OR "intervention condition" OR "control condition" OR "treatment group" OR "control participant" OR "quasi experimental design" OR "experimental condition" OR "treatment condition" OR "pilot projects" OR "feasibility studies" OR "program evaluation" OR "pre test" OR "post test" OR "quasi experiment*" OR blinded OR "double bind" OR fidelity OR reach OR uptake OR implementation OR "intervention effect*" OR "program effect*" OR "programme effect*" OR "proof of concept*" OR "outcome study"))
#7	#5 AND #6
#8	#2 OR #3
#9	#4 OR #7
#10	#8 AND #9

#11	TS=(("obesity" OR "obes*" OR "body mass index" OR "BMI" OR "bmi z score" OR "bmi z score" OR "body mass index z-score" OR "body mass index z-score" OR "bmi percentile*" OR "bmi centile*" OR "body mass index percentile*" OR "body mass index centile*" OR "body weight" OR "body size" OR "weight*" OR "waist" OR "adipos*" OR "body composition" OR "overweight" OR "body weight changes" OR "healthy weight" OR "weight loss" OR "weight gain"))
#12	#1 AND #10 AND #11<br>and 2023 and 2022<br>and 2021 or 2020 or 2019 or 2018 or 2017 or 2016 or 2015 or 2014 or 2013 or 2012 or 2011 (Publication Years)

SCOPUS

1	(TITLE-ABS ((preschool* OR pre-school* OR {pre-school*} OR kindergar?en OR prekinder* OR pre kinder* OR {pre Kinder} OR headstart OR {head start} OR surestart OR {sure start} OR {family child care home} OR {family childcare home} OR infant* OR toddler* OR {child care} OR {child-care} OR childcare OR {early learning centre} OR {early child} OR {early care and education} OR (child* AND {day care} OR daycare OR {day-care}) OR playgroup* OR nurser* OR {child day care centre*}))) AND (((TITLE-ABS ((diet* OR dietary OR food* OR snack* OR fruit* OR drink* OR beverage* OR juice* OR vegetable* OR m eal* OR nutrition* OR intake* OR consum* OR {food quality} OR {food preference} OR {energy density} OR {lifestyle behavio?r} OR {eating behavio?r} OR {healthy eating} OR {feeding behavio?r} OR {health promotion} OR {eating habit*} OR {food habit*} OR {food choice*} OR {overeat*} OR {over eat*} OR {weight control} OR {menu planning} OR {menu choice} OR {meal choice} OR {meal*} OR {meal planning} OR {eating habit*}))) OR (TITLE-ABS (({physical* activ*} OR playtime OR sedentary OR exercise* OR play* OR {physical fitness} OR movement OR playtime OR sport*)))) AND ((TITLE-ABS (intervention* OR {weight reduction program*} OR program* OR programme* OR project* OR therapeutics OR scheme OR initiative OR strateg* OR lifestyle OR service* OR {pilot project*} OR {feasibility stud*} OR {program evaluation} OR {health education*} OR {health promotion} OR "treat*" OR {primary prevention} OR "random*" OR {cluster random*} OR trial* OR evaluat* OR effective* OR {mixed methods} OR impact*)) OR ((TITLE-ABS (intervention* OR {weight reduction programs} OR program* OR programme* OR project* OR therapeutics OR scheme OR initiative* OR strateg* OR service* OR {health education} OR {health promotion} OR {primary prevention} OR {health education} OR treat* OR therapy* OR {pilot projects} OR {feasibility studies} OR {program evaluation})) AND (TITLE-ABS (random* OR {cluster random*} OR trial* OR evaluat* OR effective* OR {mixed methods} OR impact* OR {Non-Randomized Controlled Trial*} OR {Pragmatic Clinical Trial} OR {controlled Clinical Trial*} OR {randomized controlled trial*} OR randomized OR randomised OR {clinical trial*} OR randomly OR {controlled study} OR {controlled trial*} OR {control group} OR {intervention group} OR {experimental group} OR {comparison group} OR {control school} OR {intervention school} OR {control community} OR {intervention community} OR {intervention condition} OR {control condition} OR {treatment group} OR {control participant} OR {quasi experimental design} OR {experimental condition} OR {treatment condition} OR {pilot projects} OR {feasibility studies} OR {program evaluation} OR {pre test} OR {post test} OR {quasi experiment*} OR blinded OR {double blind} OR fidelity OR reach OR uptake OR implementation OR {intervention effect*} OR {program effect*} OR {programme effect*} OR {proof of concept*} OR {outcome study}))))) AND (TITLE-ABS ((obesity OR obes* OR {body mass index} OR {BMI} OR {bmi z score} OR {bmi z score} OR {body mass index z-score} OR {body mass index z-score} OR {bmi percentile*} OR {bmi centile*} OR {body mass index percentile*} OR {body mass index centile*} OR {body weight} OR {body size} OR weight* OR waist OR adipos* OR {body composition} OR overweight OR {body weight changes} OR {healthy weight} OR {weight loss} OR {weight gain}))) AND (LIMIT-TO (PUBYEAR , 2023) OR LIMIT-TO (PUBYEAR , 2022) OR LIMIT-TO (PUBYEAR , 2021) OR LIMIT-TO (PUBYEAR , 2020) OR LIMIT-TO (PUBYEAR , 2019) OR LIMIT-TO (PUBYEAR , 2018) OR LIMIT-TO (PUBYEAR , 2017) OR LIMIT-TO (PUBYEAR , 2016) OR LIMIT-TO (PUBYEAR , 2015) OR LIMIT-TO (PUBYEAR , 2014) OR LIMIT-TO (PUBYEAR , 2013) OR LIMIT-TO (PUBYEAR , 2012) OR LIMIT-TO (PUBYEAR , 2011))

PsychINFO

**1**	(((preschool* or pre-school* or pre-school* or kindergar$en or prekinder* or pre-Kinder* or pre Kinder* or headstart or head start or surestart or sure start or family child care home or family childcare home or infant* or toddler* or child care or child-care or childcare or early learning centre or early child* or early care) and education) or ((child* and day care) or daycare or day-care or playgroup* or nurser* or child day care centre*)).mp. [mp=title, abstract, heading word, table of contents, key concepts, original title, tests & measures, mesh]
**2**	(diet* or dietary or food* or snack* or fruit* or drink* or beverage* or juice* or vegetable* or meal* or nutrition* or intake* or consum* or food quality or food preference* or energy density or lifestyle behavio?r or eating behavio?r or healthy eating or feeding behaviour?r or health promotion or eating habit* or food habit* or food choice* or overeat* or over eat* or weight control or menu planning or menu choice or meal choice* or meal* or meal planning or (food* and consum*) or eating habit*).mp. [mp=title, abstract, heading word, table of contents, key concepts, original title, tests & measures, mesh]
**3**	(physical* activ* or playtime or sedentary or exercise* or play* or physical fitness or movement or playtime or sport*).mp. [mp=title, abstract, heading word, table of contents, key concepts, original title, tests & measures, mesh]
**4**	(intervention* or weight reduction program* or program* or programme* or project* or therapeutics or scheme or initiative* or strateg* or life style or service* or pilot project* or feasibility stud* or program evaluation or health education* or health promotion or primary prevention or treat* or random* or cluster random* or trial* or evaluat* or effective* or mixed methods or impact*).mp. [mp=title, abstract, heading word, table of contents, key concepts, original title, tests & measures, mesh]
**5**	(intervention* or weight reduction programs or program*OR programme* or project* or therapeutics or scheme or initiative* or strateg* or service* or health education or health promotion or primary prevention or health education or health promotion or primary prevention or treat* or therapy* or pilot projects or feasibility studies or program evaluation).mp. [mp=title, abstract, heading word, table of contents, key concepts, original title, tests & measures, mesh]
**6**	(random* or cluster random* or trial* or evaluat* or effective* or mixed methods or impact* or Non-Randomized Controlled Trial* or Pragmatic Clinical Trial or controlled Clinical Trial* or randomized controlled trial* or randomized or randomised or clinical trial* or randomly or controlled study or controlled trial* or control group or intervention group or experimental group or comparison group or control school or intervention school or control community or intervention community or intervention condition or control condition or treatment group or control participant or quasi experimental design or experimental condition or treatment condition or pilot projects or feasibility studies or program evaluation or pre test or post test or quasi experiment* or blinded or double blind or fidelity or reach or uptake or implementation or intervention effect* or program effect* or programme effect* or proof of concept* or outcome study).mp. [mp=title, abstract, heading word, table of contents, key concepts, original title, tests & measures, mesh]
**7**	5 and 6
**8**	2 or 3
**9**	4 or 7
**10**	8 and 9
**11**	(obesity or obes* or body mass index or BMI or bmi z score or bmi z score or body mass index z-score or body mass index z-score or bmi percentile* or bmi centile* or body mass index percentile* or body mass index centile* or body weight or body size or weight* or waist or adipos* or body composition or overweight or body weight changes or healthy weight or weight loss or weight gain).mp. [mp=title, abstract, heading word, table of contents, key concepts, original title, tests & measures, mesh]
**12**	1 and 10 and 11

**13**	limit 12 to last 12 years

Social Policy and Practice

**1**	(((preschool* or pre-school* or pre-school* or kindergar$en or prekinder* or pre-Kinder* or pre Kinder* or headstart or head start or surestart or sure start or family child care home or family childcare home or infant* or toddler* or child care or child-care or childcare or early learning centre or early child* or early care) and education) or ((child* and day care) or daycare or day-care or playgroup* or nurser* or child day care centre*)).mp. [mp=abstract, title, publication type, heading word, accession number]
**2**	(diet* or dietary or food* or snack* or fruit* or drink* or beverage* or juice* or vegetable* or meal* or nutrition* or intake* or consum* or food quality or food preference* or energy density or lifestyle behavio?r or eating behavio?r or healthy eating or feeding behaviour?r or health promotion or eating habit* or food habit* or food choice* or overeat* or over eat* or weight control or menu planning or menu choice or meal choice* or meal* or meal planning or (food* and consum*) or eating habit*).mp. [mp=abstract, title, publication type, heading word, accession number]
**3**	(physical* activ* or playtime or sedentary or exercise* or play* or physical fitness or movement or playtime or sport*).mp. [mp=abstract, title, publication type, heading word, accession number]
**4**	(intervention* or weight reduction program* or program* or programme* or project* or therapeutics or scheme or initiative* or strateg* or life style or service* or pilot project* or feasibility stud* or program evaluation or health education* or health promotion or primary prevention or treat* or random* or cluster random* or trial* or evaluat* or effective* or mixed methods or impact*).mp. [mp=abstract, title, publication type, heading word, accession number]
**5**	(intervention* or weight reduction programs or program*OR programme* or project* or therapeutics or scheme or initiative* or strateg* or service* or health education or health promotion or primary prevention or health education or health promotion or primary prevention or treat* or therapy* or pilot projects or feasibility studies or program evaluation).mp. [mp=abstract, title, publication type, heading word, accession number]
**6**	(random* or cluster random* or trial* or evaluat* or effective* or mixed methods or impact* or Non-Randomized Controlled Trial* or Pragmatic Clinical Trial or controlled Clinical Trial* or randomized controlled trial* or randomized or randomised or clinical trial* or randomly or controlled study or controlled trial* or control group or intervention group or experimental group or comparison group or control school or intervention school or control community or intervention community or intervention condition or control condition or treatment group or control participant or quasi experimental design or experimental condition or treatment condition or pilot projects or feasibility studies or program evaluation or pre test or post test or quasi experiment* or blinded or double blind or fidelity or reach or uptake or implementation or intervention effect* or program effect* or programme effect* or proof of concept* or outcome study).mp. [mp=abstract, title, publication type, heading word, accession number]
**7**	5 and 6
**8**	2 or 3
**9**	4 or 7
**10**	8 and 9
**11**	(obesity or obes* or body mass index or BMI or bmi z score or bmi z score or body mass index z-score or body mass index z-score or bmi percentile* or bmi centile* or body mass index percentile* or body mass index centile* or body weight or body size or weight* or waist or adipos* or body composition or overweight or body weight changes or healthy weight or weight loss or weight gain).mp. [mp=abstract, title, publication type, heading word, accession number]
**12**	1 and 10 and 11
**13**	limit 12 to last 12 years

HMIC Health Management Information Consortium

**1**	(((preschool* or pre-school* or pre-school* or kindergar$en or prekinder* or pre-Kinder* or pre Kinder* or headstart or head start or surestart or sure start or family child care home or family childcare home or infant* or toddler* or child care or child-care or childcare or early learning centre or early child* or early care) and education) or ((child* and day care) or daycare or day-care or playgroup* or nurser* or child day care centre*)).mp. [mp=title, other title, abstract, heading words]
**2**	(diet* or dietary or food* or snack* or fruit* or drink* or beverage* or juice* or vegetable* or meal* or nutrition* or intake* or consum* or food quality or food preference* or energy density or lifestyle behavio?r or eating behavio?r or healthy eating or feeding behaviour?r or health promotion or eating habit* or food habit* or food choice* or overeat* or over eat* or weight control or menu planning or menu choice or meal choice* or meal* or meal planning or (food* and consum*) or eating habit*).mp. [mp=title, other title, abstract, heading words]
**3**	(physical* activ* or playtime or sedentary or exercise* or play* or physical fitness or movement or playtime or sport*).mp. [mp=title, other title, abstract, heading words]
**4**	(intervention* or weight reduction program* or program* or programme* or project* or therapeutics or scheme or initiative* or strateg* or life style or service* or pilot project* or feasibility stud* or program evaluation or health education* or health promotion or primary prevention or treat* or random* or cluster random* or trial* or evaluat* or effective* or mixed methods or impact*).mp. [mp=title, other title, abstract, heading words]
**5**	(intervention* or weight reduction programs or program*OR programme* or project* or therapeutics or scheme or initiative* or strateg* or service* or health education or health promotion or primary prevention or health education or health promotion or primary prevention or treat* or therapy* or pilot projects or feasibility studies or program evaluation).mp. [mp=title, other title, abstract, heading words]
**6**	(random* or cluster random* or trial* or evaluat* or effective* or mixed methods or impact* or Non-Randomized Controlled Trial* or Pragmatic Clinical Trial or controlled Clinical Trial* or randomized controlled trial* or randomized or randomised or clinical trial* or randomly or controlled study or controlled trial* or control group or intervention group or experimental group or comparison group or control school or intervention school or control community or intervention community or intervention condition or control condition or treatment group or control participant or quasi experimental design or experimental condition or treatment condition or pilot projects or feasibility studies or program evaluation or pre test or post test or quasi experiment* or blinded or double blind or fidelity or reach or uptake or implementation or intervention effect* or program effect* or programme effect* or proof of concept* or outcome study).mp. [mp=title, other title, abstract, heading words]
**7**	5 and 6
**8**	2 or 3
**9**	4 or 7
**10**	8 and 9
**11**	(obesity or obes* or body mass index or BMI or bmi z score or bmi z score or body mass index z-score or body mass index z-score or bmi percentile* or bmi centile* or body mass index percentile* or body mass index centile* or body weight or body size or weight* or waist or adipos* or body composition or overweight or body weight changes or healthy weight or weight loss or weight gain).mp. [mp=title, other title, abstract, heading words]
**12**	1 and 10 and 11
**13**	limit 12 to last 12 years

Trials Register of Promoting Health Interventions (TRoPHI)

saved 551 records from the following searches:

Search 1:

2343: 2341 AND 2342 361 records (of which saved records from year 2011 onwards)

2342: Coded with: children (0-10 yrs) 1933

2341: Coded with: obesity 1194

Search 2:

2366: 2365 AND 2329 (unscreened records): 123 records

2365: 2364 AND 2347 1116

2347: "toddler* OR young child* OR preschool* OR infant* OR child*" (in Title and Abstract)

2364: Coded with: Weight Loss, OR Physical Activity

Search 3:

2344: AND 2347 AND 2355 AND 2338: 221 records

2344: Not coded with: EPPI-Centre Health promotion keywording strategy

2347: "toddler* OR young child* OR preschool* OR infant* OR child*" (in Title and Abstract)

2355: "obes* OR sedentar* OR physical* OR diet* OR exercise OR BMI OR "body weight" OR "body size" OR waist " (in Title and Abstract)

2338: Coded with: Include: Randomised controlled trial, OR Include: Non-Random 14391

Search 4: 136 records: (of which saved records from year 2010 onwards)

"obes* OR sedentar* OR physical* OR diet*" (in Title and Abstract)

AND

"exercise OR BMI OR "body weight" OR "body size" OR waist " (in Title and Abstract)

AND

"toddler* OR young child* OR preschool* OR infant* OR child*" (in Title and Abstract)

AND

Not coded with: EPPI-Centre Health promotion keywording strategy

AND

Not coded with: Ex 1: Not Health Promotion or Public Health, OR Ex 2: Not Human or Environment, OR Ex 3: NOT an evaluation of an intervention, OR Ex: 4 No control or comparison group, OR Ex 5: Item is a review or meta-analysis, OR MARKER: QUERY, OR Duplicate

Appendix 2

Depiction of the screening process

The graph below depicts the screening process on tittle and abstract. The prioritisation of records was refreshing regularly. The most relevant articles were screened first. The screening stopped when the graph reached a plateau.

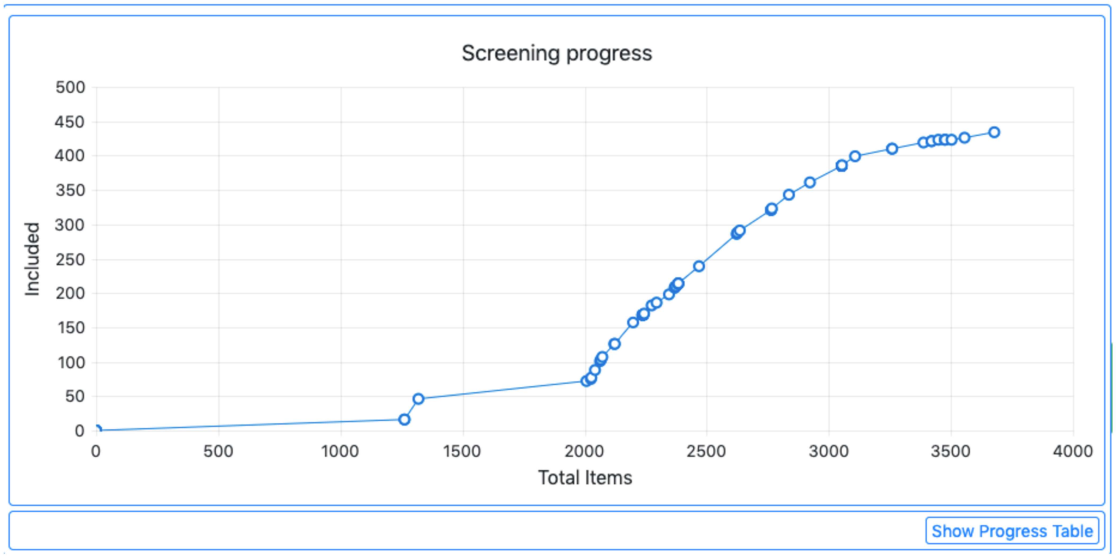

Appendix 3

Bias assessment

Table S2: Studies assessed using the ROB tool

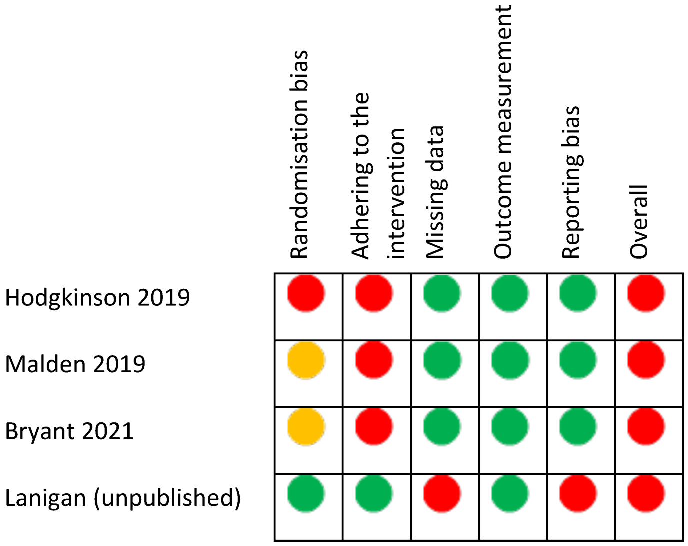

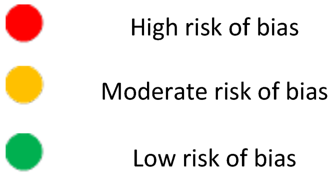

Table S3: Studies assessed using the ROBINS-I tool

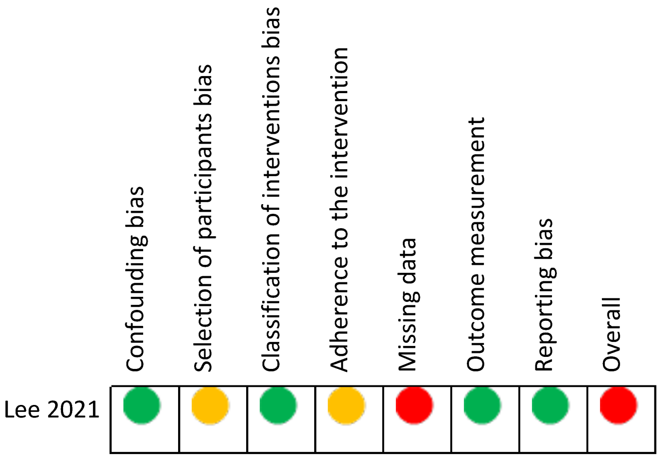

Supplement: Supplementary data [file bmjopen-2023-076479supp001.pdf]
