# Supplementary material for: Assessing the impact of obesity interventions in the early years: a systematic review of UK-based studies
Source: BMJ Open. 2024 May 13;14(5):e076479. doi: 10.1136/bmjopen-2023-076479 (PMC11097867; doi:10.1136/bmjopen-2023-076479)
Supplement: Author's manuscript [file bmjopen-2023-076479.draft_revisions.pdf]

BMJ Open

BMJ Open is committed to open peer review. As part of this commitment we make the peer review history of every article we publish publicly available.

When an article is published we post the peer reviewers' comments and the authors' responses online. We also post the versions of the paper that were used during peer review. These are the versions that the peer review comments apply to.

The versions of the paper that follow are the versions that were submitted during the peer review process. They are not the versions of record or the final published versions. They should not be cited or distributed as the published version of this manuscript.

BMJ Open is an open access journal and the full, final, typeset and author-corrected version of record of the manuscript is available on our site with no access controls, subscription charges or pay-per-view fees (http://bmjopen.bmj.com).

If you have any questions on BMJ Open's open peer review process please email info.bmjopen@bmj.com

Assessing the effectiveness of obesity interventions in the early years: A systematic review of UK-based studies

Journal:	*BMJ Open*
Manuscript ID	bmjopen-2023-076479
Article Type:	Original research
Date Submitted by the Author:	08-Jun-2023

Complete List of Authors:	Michalopoulou, Semina; University College London, Great Ormond Street Institute of Child Health<br>Sifaki, Maria; University College London, Great Ormond Street Institute of Child Health<br>Packer, Jessica; University College London, Great Ormond Street Institute of Child Health<br>Lanigan, Julie; University College London, Great Ormond Street Institute of Child Health<br>Stansfield, Claire; University College London, Institute of Education<br>Russell, Simon; University College London, Great Ormond Street Institute of Child Health<br>Viner, Russell; University College London, Great Ormond Street Institute of Child Health

Keywords:	Obesity, NUTRITION & DIETETICS, Community child health < PAEDIATRICS

SCHOLARONE™ Manuscripts

I, the Submitting Author has the right to grant and does grant on behalf of all authors of the Work (as defined in the below author licence), an exclusive licence and/or a non-exclusive licence for contributions from authors who are: i) UK Crown employees; ii) where BMJ has agreed a CC-BY licence shall apply, and/or iii) in accordance with the terms applicable for US Federal Government officers or employees acting as part of their official duties; on a worldwide, perpetual, irrevocable, royalty-free basis to BMJ Publishing Group Ltd ("BMJ") its licensees and where the relevant Journal is co-owned by BMJ to the co-owners of the Journal, to publish the Work in this journal and any other BMJ products and to exploit all rights, as set out in our licence.

The Submitting Author accepts and understands that any supply made under these terms is made by BMJ to the Submitting Author unless you are acting as an employee on behalf of your employer or a postgraduate student of an affiliated institution which is paying any applicable article publishing charge ("APC") for Open Access articles. Where the Submitting Author wishes to make the Work available on an Open Access basis (and intends to pay the relevant APC), the terms of reuse of such Open Access shall be governed by a Creative Commons licence – details of these licences and which Creative Commons licence will apply to this Work are set out in our licence referred to above.

Other than as permitted in any relevant BMJ Author's Self Archiving Policies, I confirm this Work has not been accepted for publication elsewhere, is not being considered for publication elsewhere and does not duplicate material already published. I confirm all authors consent to publication of this Work and authorise the granting of this licence.

For peer review only

Assessing the effectiveness of obesity interventions in the early years: A systematic review of UK-based studies

Semina Michalopoulou,[1] Maria Sifaki,[1] Jessica Packer,[1] Julie Lanigan,[1] Clare Stansfield,[2] Russell Viner[1]* and Simon Russell[1]*

*co senior authors

[1] UCL Great Ormond Street Institute of Child Health, University College London, London, UK,

[2] UCL Institute of Education, University College London, London, UK

Corresponding author:

Semina Michalopoulou

UCL Great Ormond Street Institute of Child Health, University College London, 30 Guilford Street, London, WC1N 1EH

Tel: 0207 905 2110

s.michalopoulou@ucl.ac.uk

Short version of the title: UK early childhood obesity interventions

Keywords: childhood obesity, interventions, diet, physical activity, early years, United Kingdom

For peer review only

Abstract

Background & Objectives: Childhood obesity rates in the UK are high. The early years of childhood are critical for establishing healthy behaviours and offer interventional opportunities. We aimed to assess the effectiveness of UK-based childhood obesity interventions.

Design: Systematic review

Setting: United Kingdom

Methods: In March 2023, nine databases were searched for UK based obesity intervention studies delivered to children younger than five years, that had diet and/or physical activity components and reported anthropometric outcomes. Studies evaluating the effects of breastfeeding or complementary feeding interventions were excluded, as these aim to support healthy growth development. Publication date was limited to the previous 12 years (2011-2023), as earlier reviews found few UK evaluations. Bias was assessed using Cochrane tools (ROB 2 or ROBINS-I).

Results: Six trials (five studies) were identified, including two randomised controlled trials (RCT), one cluster randomised trial (CRT), two feasibility CRTs, and one impact assessment. The total number of participants was 566. Three trials focused on disadvantaged families and two included high-risk children categorised as having overweight or obesity. The outcome of interest was zBMI change (within and between subjects). Compared to baseline, five interventions reported reductions in zBMI, three of which were statistically significant. Compared to control, five interventions showed zBMI reductions, one of which was significant. Only two trials were followed up beyond 12 months and only one complied with the National Institute's Health and Care Excellence guidelines.

For peer review only

Conclusion: Though interventions produced promising results in terms of reducing obesity risk, the evidence was limited, and further assessment is required before scaling out.

PROSPERO registration number: CRD42021290676

Strengths and limitations of this study:

- This is the first systematic review that focuses on childhood obesity interventions among reception-aged children evaluated in the UK.
- Only studies published after 2011 were included.
- Six trials (five studies) were identified after the searches on nine scientific databases. The evidence is limited, however promising effects were found.
- The risk of bias assessment was conducted with for all the included studies with tools suitable for the randomised and the non-randomised studies.
- A meta-analysis was not possible due to the heterogeneity of the interventions

Funding statement: This work was supported by the National Institute for Health Research Policy Research Programme (award number: 174868). National Institute for Health Research had no role in the design, analysis or writing of this article. The views expressed in this publication are those of the authors and not necessarily those of the NHS, the National Institute for Health Research, the Department of Health and Social Care or its arm's length bodies, and other Government Departments.

Competing interests statement: None

For peer review only

INTRODUCTION

In England, data from the National Child Measurement Programme (NCMP) show that the prevalence of obesity among reception-aged children (4-5 years), though plateaued, remains stably high. Disadvantaged children are the worst affected, as the inequalities in childhood obesity are increasing.(1,2) Once established, obesity tracks strongly through childhood, adolescence, and into adulthood where it is associated with increased risk of physical and mental health morbidities.(3–7) Prospective evidence indicates that among adolescents with obesity, most excessive weight gain occurred between 2 and 6 years.(8) Data from the NCMP suggest that the odds of children with overweight in reception developing obesity by 10 and 11 years were 13 times higher compared to children with healthy BMI. Longitudinal evidence shows that a child's weight status remains stable through primary school years, as 68% of children with obesity and 78% of children with healthy weight at reception maintained their weight status by 10 and 11 years.(9) The early years of life are therefore a critical period for the development of a healthy weight, representing a window of opportunity for the introduction of a healthy lifestyle to support healthy growth.

Diet(10,11) and physical activity(12) are the major determinants of weight in young children. The diets of many pre-schoolers in the UK do not adhere to government guidelines, exceeding intake of free sugar and saturated fat, and not reaching recommendations for fruit and vegetables.(13,14) Physical activity levels are reported to be below recommended guidelines(15) with most children not engaging in the 180 minutes of physical activity per day recommended by the National Health Service (NHS).(16) Lifestyle behaviours track through childhood, into adolescence, and adulthood, meaning that those children are likely to continue

For peer review only

following unhealthy practices, thereby being at even greater risk of overweight and obesity.(17,18)

Considering the significance of the preschool period for outcomes later in life, interventions to support the establishment of a healthy weight and a healthy lifestyle are valuable. In the UK, the National Institute for Health and Care Excellence (NICE)(19) gives guidance on the key interventional components to reduce the risk or manage overweight and obesity. Principally, these include the adoption of a healthy diet, addressing lifestyle risk factors within the family and social settings, i.e., preschools, reducing sedentary lifestyle, and supporting behavioural change. The UK Government's Tackling Obesity Policy Paper(20) aims to place prevention at the heart of the health agenda to proactively tackle the burden of obesity for adults and children. The Paper suggests that an integrated, whole systems approach is likely to be the most effective in addressing the complex pathways to obesity. It is acknowledged that controlling the problem of childhood obesity is a shared responsibility of the Government, local authorities, and the NHS, in addition to the food industry, schools, and local communities.

The high prevalence of childhood obesity in the UK supports the need for early interventions. To inform such interventions it is crucial to understand which interventions have been successful. It is also important to identify interventions with sustainable effects, i.e., have a long-term follow-up. The aim of this review was to synthesise evidence of interventions implemented and evaluated in the UK that aim to prevent or treat obesity in the early years of life. We restricted this review to UK interventions as early childhood environments can differ markedly across countries, and interventions implemented in the UK have prima facie evidence of feasibility.

For peer review only

MATERIALS AND METHODS

Design

This systematic review adhered to the Preferred Reporting Items for Systematic Reviews and Meta-Analyses (PRISMA) statement(21) and presents the UK findings of the PROSPERO-registered protocol CRD42021290676.

Search Strategy

In November 2021, we searched nine databases for obesity interventions among children under five years old (selected databases and search terms used are shown in Appendix 1). The searches were updated in March 2023. In addition to database searches, the authors searched reference lists of similar reviews. No language restrictions were applied. Our searches included international literature; however, here we report findings from UK studies only. Applicability and transferability of the interventions conducted elsewhere might be limited due to variability in obesity prevalence, resource availability, policy landscape, and delivery of the programmes.(22)

Inclusion and exclusion criteria

Primary research studies were included if they fulfilled the following criteria:

Population: children aged 0-5 years.

Intervention: Pre- and post-intervention studies including randomised controlled trials, quasi-experimental studies, (individual, family- or community-based).

Comparison: pre- vs post-intervention and/or intervention vs control group.

Outcomes: at least one anthropometric outcome (e.g., BMI, BMI z-score, BMI percentile, waist circumference, skinfold measurements). Anthropometric measurements were chosen as an

For peer review only

outcome because these are the most robust way of assessing effectiveness, compared to other potential outcomes (e.g., diet or physical activity).

The search was limited to studies published during the previous 12 years (2011-2023) to reflect current or recent interventions and programmes, to understand what works in the current context. Previous reviews found no studies conducted in the UK, meaning it is unlikely that there were UK interventions robustly evaluated prior to 2011.(23,24)

Selection of studies and data extraction

Search results were imported into Endnote, where duplicates were removed, before being imported into EPPI Reviewer Web,(25) in which further duplicate searches were undertaken. Two reviewers (SM and MS) independently double screened on title and abstract. Using EPPI-Reviewer software, we applied an ‘active learning approach’, where the prioritisation of records was periodically refreshed during screening so that the most relevant articles were screened first.(26) A graphical output was used to indicate when to stop screening, i.e., when the number of relevant studies had plateaued (Appendix 2). A classifier model was then built using the machine learning algorithm and applied to unscreened items, which generates a score indicating relevance; this process reduces the likelihood that relevant studies would be missed. While searches included children aged 0-5, interventions that targeted infant feeding were excluded, as their scope was predominantly infant development rather than obesity prevention/treatment. The reviewers developed a data extraction tool and data were doubly extracted. The data extracted included information on trial identification (name of study, authors, year of publication), country (country the trial took place and country of research), trial description, trial duration and follow-up, trial characteristics (preventative/treatment,

For peer review only

intervention/programme), participants' characteristics (numbers, ages), and results regarding changes on zBMI. The reviewers double screened independently, and any disagreements were resolved through discussion.

Data synthesis

We performed a narrative synthesis of findings, summarising the effect estimates; meta-analysis was not possible owing to heterogeneity in intervention approaches, i.e., components of the interventions, participants targeted, site of delivery, duration, intensity, and length of follow-up. The included studies are presented in two sections: "preventative" or "treatment", based on their inclusion criteria. Universal interventions with children of any weight status are included in the "preventative" category, while interventions that recruited children with overweight or obesity only are classified as "treatment" interventions. We report the mean difference of any anthropometric outcomes both between-group (i.e., intervention vs. control), and within-group (i.e., before vs. after). Measurements obtained at any time point (e.g., mid-intervention, right after the intervention, after the end of the intervention) were considered for this review. If studies provided the mean value of anthropometric outcomes before and after the intervention or in the control and the intervention group, two reviewers (SM &MS) calculated the mean difference and the standard deviation of the change using formulas that are suggested by the Cochrane Handbook.(27)

Risk of bias assessment

Two reviewers (SM and MS) independently assessed the bias using the Cochrane risk of bias assessment tool (RoB 2) to evaluate the included randomised trials.(28) For one study that was

For peer review only

an impact assessment, the ROBINS-I tool was used which is suitable for non-randomised studies.(29) Detailed bias assessment can be found in Appendix 3.

Patient and Public Involvement

An initial patient and public involvement (PPI) session was conducted at the outset of the project. We discussed with a Family Research Advisory Group, facilitated by the National's Children's Bureau (NCB),(30) the evidence of current childhood obesity interventions in the UK and what parents/public contributors perceived to be the elements or components of interventions that made them effective. A second session was conducted at the end of the project, where review findings were discussed. Parents/public contributors discussed the challenges of implementing effective interventions, how to effectively evaluate interventions in the early years, and the importance of parental involvement in effective interventions.

RESULTS

Description of the studies

In total, the literature search yielded 34,572 potentially relevant articles. After duplicates removal, 21,547 articles were eligible for screening. Two independent reviewers (SM & MS) manually screened 9,538 records, in title and abstract, of which 6,217 were manually removed (Figure 1). Any differences were resolved by reconciliation and, if necessary, in consultations with the other authors. An additional 12,004 records were removed based on title and abstract, as indicated by the machine learning algorithm. A total of 3,326 records were included for full-text screening, two of which could not be retrieved, as we could not gain access to them. Out of the 3,324 remaining records, 3,108 were manually excluded (reasons for exclusion presented in

For peer review only

Figure 1), leaving 217 relevant studies. Out of the 217 international studies, five studies (six trials) were conducted in the UK and were included in the final review.(31–35)

One study was conducted in Lancashire,(31) one in Glasgow,(32) one in two areas in North and Central England, (33) one in Cornwall,(34) and one in Hertfordshire(35). There were two randomised controlled trials,(35) one cluster randomised trial (CRT),(31) two feasibility CRTs,(32,33) and one before/after impact assessment of a weight management programme.(34) Sample sizes ranged from 42 to 117. Intervention duration ranged from eight weeks to 12 months. The follow-up measurements were obtained during the intervention for one study(32) and after the intervention for the rest, ranging from 6 to 30 months from the start of the intervention. Two trials targeted children living with overweight, obesity, or at high risk, (34),(35) while the remaining four were preventative interventions,(31–33,35) and targeted children of any weight status (children in the general population, not necessarily living with overweight or obesity). Detailed information for each of the included trials can be found in Table 1.

[Figure 1]

Intervention characteristics and effects

Preventative interventions

The four preventative trials include the *Be active, Eat healthy/Healthy Heroes*(31) intervention, the *ToyBox*(32) intervention, the *Health Exercise Nutrition for the Really Young (HENRY)*(33) programme, and the *Planet Munch* programme, trial 2.(35)

Three out of four trials (Be Active, Eat healthy/Healthy Heroes, ToyBox, HENRY) focused on disadvantaged families or deprived areas.(31–33) Most (31–33)(Be Active, Eat healthy/Healthy

For peer review only

209 Heroes, ToyBox, HENRY) were administered by preschool staff, which had been appropriately trained, while one (Planet Munch)(35) was delivered by a trained multidisciplinary team including dietitians, nutritionists, community artists, and children's centre staff. Two interventions targeted and made changes in preschools' policies; for example, by informing the food policy(31) (Be Active, Eat healthy/Healthy Heroes) or by increasing the time allocated to physical activity (ToyBox).(32) Interventions also targeted the home environment, for example by providing healthy cooking sessions(31,35) (Be Active, Eat healthy/Healthy Heroes, Planet Munch) or sending materials for home-based physical activities. (ToyBox, Planet Munch).(32,35) One study(33) (HENRY) was delivered to parents through workshops organized in early years centres, with recommendations provided on nutrition, physical activity, and parenting. Planet Munch(35) included workshops for parents and children, that aimed to help them improve their dietary and physical activity practices and adopt a healthier lifestyle.

All studies involved a control group; in the majority of cases, the control group received standard care (Be Active, Eat healthy/Healthy Heroes, ToyBox, HENRY),(31–33) while in one study (Planet Munch),(35) the control group was a delayed intervention group. The risk assessment revealed that *Be active, eat healthy/Healthy Heroes*, *ToyBox*, *HENRY and Planet Munch* had a high-risk of bias.

Only one trial (Be Active, Eat healthy/Healthy Heroes)(31) found the intervention group to have significantly decreased their zBMI after the intervention, compared to the control group [-0.74, (-1.10 to -0.38)]. Two studies(33,35) also found a lower zBMI in the intervention compared to the control group; however, there was either no statistical comparison between them (HENRY),(33) or the difference was not statistically significant (Planet Munch).(35) In one

For peer review only

trial(32) (ToyBox) the zBMI had been raised for both groups; nonetheless, this increase was larger for the control group (Table 1).

When looking at zBMI changes within the intervention group, three studies showed reductions, ranging from -0.02 (-0.2 to 0.2) to -0.9 (95% CIs not given) (Be Active, Eat healthy/Healthy Heroes, HENRY, Planet Munch).(31,33,35) In one study, this reduction was statistically significant (Be Active, Eat healthy/Healthy Heroes),(31) in one no statistical comparison was reported (HENRY),(33) and one study found the reduction not to be significant (Planet Munch).(35) Finally, one trial (Toy Box)(32) reported zBMI to have increased by 0.02 (-0.11 to 0.15). A second follow-up measurement took place in one of the studies (Planet Munch);(35) while there was a decrease compared to baseline, this was not significant (Table 1).

[Table 1]

Overweight and obesity treatment interventions

We identified two treatment trials. The first was *The Lifestyles, Eating, and Activity for Families (LEAF)*(34) programme, which was an impact assessment of a childhood obesity service and the treatment trial of *Planet Munch*.(35) *The LEAF programme* targeted children with severe obesity or severe obesity with a relevant comorbidity. The programme consisted of an initial home visit, workshops, and follow-up clinic appointments. A community paediatrician, a specialist dietitian, and a physical activity advisor were involved for each child. There was no control group in LEAF. In terms of within-subject changes, the *LEAF* programme found a significant reduction of zBMI, following programme participation (Table 1). The trial was assessed to have a high risk of bias mainly due to missing data.

The treatment trial of *Planet Munch* targeted children with overweight/obesity, or who were high-risk. As with the prevention trial, it was delivered by a multi-disciplinary team through healthy lifestyle workshops. Planet Munch included a control group (delayed intervention group). At the end of the intervention, after accounting for age and gender, the intervention group had lower mean zBMI score than the control group. For Planet Munch, there was a significant reduction in the zBMI of the intervention group, from baseline to the end of the intervention, as well as from baseline to the 2-year follow-up (Table 1)

DISCUSSION

The aim of this systematic review was to identify and evaluate interventions and programmes designed to prevent or treat obesity among children aged 0-5 years, implemented and evaluated in the UK. Six studies met the inclusion criteria, all of which included a combination of diet and physical activity components. Compared to baseline, three interventions reported a reduction in zBMI immediately after completing the intervention(34,35) and four interventions a while after the intervention end,(31,33,35) with the effect ranging from -0.02 to -0.9 for the preventative interventions and from -0.3 to -0.5 for the treatment ones. Three of these reductions were significant(31,34,35) (two of which targeted children with overweight/obesity).(34,35) Compared to the control conditions, five interventions showed a reduction in zBMI,(31–33,35) of which one was significant.(31)

This is the first systematic review that focuses on childhood obesity interventions among reception-aged children evaluated in the UK. Our findings support existing evidence from international reviews that multicomponent interventions, with both dietary behaviours and physical activity elements, are effective in reducing zBMI among pre-schoolers.(24,36,37) Small

effects were found at individual level; however, if the interventions are delivered at population level, the effects can be larger and meaningful.(38) The present work contributes to the research and policy by synthesizing the evidence of recent interventions and assessing their effects on children of any weight status or on children with overweight and obesity.

The current review highlights a lack of UK interventions with robust evaluation. Only Planet Munch was evaluated through a randomised controlled trial. ToyBox and HENRY were evaluated with feasibility cluster randomised trials without a follow-on full trial being published, and these feasibility trials were not adequately powered to detect zBMI changes. Methodological limitations across the studies identified, such as unclear adherence to the intervention, limit our ability to draw firm conclusions about their effectiveness. Evaluating interventions is complex; it is important not only to ascertain effectiveness, but also when, how, and in which conditions and for whom interventions work. A theoretical framework, a process to identify failures in implementation, an assessment of the sample, and a range of outcomes, in addition to adaptation to local settings, are important contributory factors to the effectiveness and applicability of an intervention.(39,40)

Of the studies identified in this review, only two were followed up beyond 12-months(31,35) meaning there is very little evidence for longer-term effects. All the studies included in this review were small-scale evaluations and not easily generalisable. While effect sizes give some measure of clinical importance (if adequately powered), statistical significance is affected by the sample size, and so has limited importance.(41) A reduction in zBMI may be clinically relevant when setting a child on a healthier growth trajectory and improving health outcomes.(42)

For peer review only

296 Overall, we found a lack of evaluations of interventions that recorded anthropometric
297 outcomes. There were evaluations that reported indirect indicators of intervention
298 effectiveness including reductions in calorie intake or increases in physical activity; however,
299 these indicators can be prone to bias. Children and parents often underreport their energy
300 intake,(43),(44) with greater underreporting often observed among children living with
301 obesity.(45) Subjective measures of physical activity also often have low validity and may be
302 biased due to poor recall or social desirability.(46) There are evaluations of interventions (e.g.,
303 the HENRY programme) that show effectiveness in changing energy balance behaviours(47) but
304 have not been included in this review.
305 Within a whole-systems approach, local authorities can play a leading role in influencing health
306 behaviour. However, it is important to recognize that financial constraints can limit local action
307 to predominantly statutory services. Owing to these and other limitations, many local
308 authorities have developed interventions relevant to the early years which have not been
309 subject to robust assessment. These are often brief interventions (e.g., cooking classes or baby
310 yoga), for which there is no evidence of meaningful and sustained lifestyle changes.
311 Published studies also had a high risk of bias, limiting the confidence in their results.
312 Additionally, findings were difficult to compare owing to heterogeneity in study designs,
313 intervention approaches, and outcome reporting. Some of the reported reductions in zBMI
314 should be interpreted with caution. For example, children that received the *HENRY* intervention
315 started the programme with higher mean zBMI than those in the control group, which may
316 have influenced the effect size. Furthermore, the control group gained weight during the
317 intervention, leading the effect size to be amplified further. This highlights the need for

For peer review only

appropriately designed RCTs with stated primary outcomes, in order to evaluate obesity interventions.

Findings from this review do suggest that UK weight management and prevention interventions for the early years can be feasible and effective in reducing the risk of obesity. However, all studies would benefit from evaluation using large-scale RCTs before firm conclusions can be drawn. Unpublished data were provided for two trials relating to the *Planet Munch* (formerly Trim Tots) intervention, which found reductions in zBMI for children living with overweight or obesity or who were at high risk. Importantly, reductions were sustained at longer-term follow-up, up to 24 months after completing the intervention. *Planet Munch* was the only preschool obesity intervention in the UK that complied with all NICE recommendations i.e., provided advice on how to achieve a healthy diet, encouraged physical activity and included behaviour change strategies in the whole family.

The *LEAF* Programme was also effective in reducing zBMI but was only delivered to children with severe obesity and was not evaluated in an RCT. Our findings are consistent with previous work,(24) which found obesity management interventions to be more effective compared to preventative interventions in young children. This is an expected outcome, considering the different populations in treatment and prevention trials. Treatment interventions aim to slow weight gain and normalise zBMI while preventative interventions aim to maintain a healthy growth trajectory. Both aim to promote healthy lifestyle behaviours.

This systematic review has limitations. The initial search strategy included international evidence without applying limits on geographical location or language and thus yielded a high number of records. We used EPPI-Reviewer software to apply an active learning approach,

For peer review only

which greatly reduced screening time but resulted in 12,004 records being excluded without being screened. Though we also conducted a hand search, there may be relevant studies that have not been included in this review. We also excluded studies published before 2011; however, previous systematic reviews have not included any UK studies conducted prior to that point, so it is unlikely that any robustly evaluated interventions had been conducted.

Only a small number of preschool obesity interventions have been implemented and evaluated in the UK. An even smaller number have been robustly evaluated in randomised controlled trials. The outcomes of some interventions suggest that they are feasible, acceptable, and have the potential to be trialled at a larger scale. However, scaling-up interventions can be challenging, as adaptations are often required, to meet real-world contexts. Intervention effects can also decrease when applied to the general population, so adaptations should consider the context, implementation and setting of the intervention.(47,48) Therefore, more research is required to understand what support might be most useful to UK pre-schoolers and their families.

CONCLUSIONS

This review found that there are very few evaluations of childhood obesity interventions in preschool children in the UK. Some interventions reported effect sizes that could be clinically significant if replicated in larger sample sizes. Effect sizes were smaller in preventative interventions where the potential for zBMI change is not as large compared to children living with excess weight at the outset. However, improvements in lifestyle behaviours were reported that may reduce the risk of excess weight gain in childhood. Interventions that aim to prevent and treat obesity in preschool children are urgently needed in the UK. Robust evaluation,

For peer review only

For peer review only - http://bmjopen.bmj.com/site/about/guidelines.xhtml

ideally in RCTs will be crucial in order to guide policy makers and inform evidence-based practice in families, in communities and local authorities in the UK.

For peer review only

Acknowledgements: We thank Dr. Julie Lanigan and all the Planet Munch team that agreed to share with us findings from their unpublished work, entitled: ‘The TrimTots Healthy Lifestyle Programme for prevention of obesity in preschool children: evidence from 2 randomised controlled trials’. Also, we would like to thank Dr. Steven Hope for commenting on the final draft of the manuscript.

Authorship confirmation: SR and RV conceived and designed the project. SM, SR and CS designed the search strategy, and SM, MS and CS conducted the searches. SM and MS conducted screening, data extraction, and assessment of bias. JL provided data from one unpublished study (two trials). SM, MS and SR analysed the data. All authors contributed to the interpretation of findings. SM drafted the paper and all authors revised and edited the manuscript, before approving the final draft.

For peer review only

For peer review only - http://bmjopen.bmj.com/site/about/guidelines.xhtml

References

1. National Child Measurement Programme, England 2020/21 School Year [Internet]. NHS Digital. [cited 2022 Nov 15]. Available from: https://digital.nhs.uk/data-and-information/publications/statistical/national-child-measurement-programme/2020-21-school-year

2. National Child Measurement Programme, England, Provisional 2021/22 School Year Outputs [Internet]. NHS Digital. [cited 2022 Nov 15]. Available from: https://digital.nhs.uk/data-and-information/publications/statistical/national-child-measurement-programme/england-provisional-2021-22-school-year-outputs

3. Umer A, Kelley GA, Cottrell LE, et al. Childhood obesity and adult cardiovascular disease risk factors: A systematic review with meta-analysis. BMC Public Health. 2017;17(1):1–24.

4. Rankin J, Matthews L, Cobley S, et al. Psychological consequences of childhood obesity: psychiatric comorbidity and prevention. Adolesc Health Med Ther. 2016;Volume 7:125–46.

5. Van Geel M, Vedder P, Tanilon J. Are overweight and obese youths more often bullied by their peers? A meta-analysis on the relation between weight status and bullying. Int J Obes. 2014;38(10):1263–7.

6. Abdelaal M, le Roux CW, Docherty NG. Morbidity and mortality associated with obesity. Ann Transl Med. 2017;5(7):1–12.

7. Simmonds M, Llewellyn A, Owen CG, et al. Predicting adult obesity from childhood obesity: A systematic review and meta-analysis. Obes Rev. 2016;17(2):95–107.

8. Geserick M, Vogel M, Gausche R, et al. Acceleration of BMI in early childhood and risk of sustained obesity. N Engl J Med. 2018;

9. Pearce M, Webb-Phillips S, Bray I. Changes in objectively measured BMI in children aged 4-11 years: Data from the National Child Measurement Programme. J Public Health U K. 2016;38(3):459–66.

10. Anderson EL, Tilling K, Fraser A, et al. Estimating trajectories of energy intake through childhood and adolescence using linear-spline multilevel models. Epidemiology. 2013;24(4):507–15.

11. Hebestreit A, Börnhorst C, Pala V, et al. Dietary energy density in young children across Europe. Int J Obes. 2014;38:S124–34.

12. Niederer I, Zahner L, Bürgi F, et al. BMI group-related differences in physical fitness and physical activity in preschool-Age children: A cross-sectional analysis. Res Q Exerc Sport. 2012;83(1):12–9.

For peer review only

For peer review only - http://bmjopen.bmj.com/site/about/guidelines.xhtml

13. Public Health England. Government Dietary Recommendations. Gov Diet Recomm. 2016;1–12.

14. NHS Digital. Health Survey for England, Fruit and Vegetable Consumption, 2018. 2019.

15. O'Dwyer M V., Foweather L, Stratton G, et al. Physical activity in non-overweight and overweight UK preschool children: Preliminary findings and methods of the Active Play Project. Sci Sports. 2011;26(6):345–9.

16. NHS England. Physical activity guidelines for children (under 5 years).

17. Ambrosini GL, Emmett PM, Northstone K, et al. Identification of a dietary pattern prospectively associated with increased adiposity during childhood and adolescence. World Rev Nutr Diet. 2014;109(2):13.

18. Jones RA, Hinkley T, Okely AD, et al. Tracking physical activity and sedentary behavior in childhood: A systematic review. Am J Prev Med. 2013;44(6):651–8.

19. National Institute for Health and Care Excellence. Obesity in children and young people : prevention and lifestyle weight management programmes. (July 2015).

20. Department of Health and Social Care. Tackling obesity: empowering adults and children to live healthier lives. 2020.

21. Page MJ, McKenzie JE, Bossuyt PM, et al. The PRISMA 2020 statement: an updated guideline for reporting systematic reviews. BMJ. 2021 Mar 29;372:n71.

22. Wang S, Moss JR, Hiller JE. Applicability and transferability of interventions in evidence-based public health. Health Promot Int. 2006;21(1):76–83.

23. Flynn MAT, McNeil DA, Maloff B, et al. Reducing obesity and related chronic disease risk in children and youth: A synthesis of evidence with 'best practice' recommendations. Vol. 7, Obesity Reviews. 2006. 7–66 p.

24. Ling J, Robbins LB, Wen F. Interventions to prevent and manage overweight or obesity in preschool children: A systematic review. Int J Nurs Stud. 2016;53:270–89.

25. Institute of Education (IOE). EPPI-Reviewer Web (Beta) [Internet]. Available from: https://eppi.ioe.ac.uk/EPPIReviewer-Web/home

26. EPPI Reviewer. Priority Screening.

27. Julian, Higgins, James T. Cochrane Handbook for Systematic Reviews of Interventions [Internet]. 6.3. 2022. Available from: https://training.cochrane.org/handbook/current

For peer review only

For peer review only - http://bmjopen.bmj.com/site/about/guidelines.xhtml

28. Sterne JAC, Savović J, Page MJ, et al. RoB 2: A revised tool for assessing risk of bias in randomised trials. The BMJ. 2019;366:1–8.

29. Sterne JA, Hernán MA, Reeves BC, et al. ROBINS-I: A tool for assessing risk of bias in non-randomised studies of interventions. BMJ Online. 2016;355:4–10.

30. National Children's Bureau [Internet]. [cited 2023 Jun 8]. Available from: https://www.ncb.org.uk/?gclid=CjwKCAjw-IWkBhBTEiwA2exyOxMt3Nt-lRUH02OtQOQLwvo-N4Oq5ZXR1Z34FpsJ6YynbaykHczGshoCUkAQAvD_BwE

31. Hodgkinson A, Abbott J, Hurley MA, et al. An educational intervention to prevent overweight in pre-school years: a cluster randomised trial with a focus on disadvantaged families. BMC Public Health. 2019 Nov 1;19(1):1430.

32. Malden S, Reilly JJ, Gibson AM, et al. A feasibility cluster randomised controlled trial of a preschool obesity prevention intervention: ToyBox-Scotland. Pilot Feasibility Stud. 2019;5(1):1–11.

33. Bryant M, Collinson M, Burton W, et al. Cluster randomised controlled feasibility study of HENRY: a community-based intervention aimed at reducing obesity rates in preschool children. Pilot Feasibility Stud. 2021;7(1):59.

34. Tee LP, Brandreth RA, Sauven N, et al. Successful outcomes in childrens specialist weight management: Impact assessment of a novel early years weight management programme. J Hum Nutr Diet. 2021;34(5):819–26.

35. Lanigan J, Collins S, Coyte K, et al. The TrimTots Healthy Lifestyle Programme for prevention of obesity in preschool children: evidence from 2 randomised controlled trials. Unpublished.

36. Brown T, Moore TH, Hooper L, et al. Interventions for preventing obesity in children. Cochrane Database Syst Rev. 2019;2019(7).

37. Colquitt JL, Loveman E, O'Malley C, et al. Diet, physical activity, and behavioural interventions for the treatment of overweight or obesity in preschool children up to the age of 6 years (Review). Cochrane Database Syst Rev. 2016;

38. Matthay EC, Hagan E, Gottlieb LM, et al. Powering population health research: Considerations for plausible and actionable effect sizes. SSM - Popul Health. 2021;14:100789.

39. Mills A, Gilson L, Hanson K, et al. What do we mean by rigorous health-systems research? The Lancet. 2008;372(9649):1527–9.

40. Craig P, Dieppe P, Macintyre S, et al. Developing and evaluating complex interventions: The new Medical Research Council guidance. Bmj. 2008;337(7676):979–83.

For peer review only - http://bmjopen.bmj.com/site/about/guidelines.xhtml

41. Dunkler D, Haller M, Oberbauer R, et al. To test or to estimate? P-values versus effect sizes. Transpl Int. 2020;33(1):50–5.

42. Ryan DH, Yockey SR. Weight Loss and Improvement in Comorbidity: Differences at 5%, 10%, 15%, and Over. Curr Obes Rep. 2017;6(2):187–94.

43. Livingstone MBE, Robson PJ, Wallace JMW. Issues in dietary intake assessment of children and adolescents. Br J Nutr. 2004;92(S2):S213–22.

44. Foster E, Hawkins A, Barton KL, et al. Development of food photographs for use with children aged 18 months to 16 years: Comparison against weighed food diaries-The Young Person's Food Atlas (UK). PLoS ONE. 2017;12(2):1–18.

45. Swinburn B, Sacks G, Ravussin E. Increased food energy supply is more than sufficient to explain the US epidemic of obesity. Am J Clin Nutr. 2009;90(6):1453–6.

46. Strath SJ, Kaminsky LA, Ainsworth BE, et al. Guide to the assessment of physical activity: Clinical and research applications: A scientific statement from the American Heart association. Circulation. 2013;128(20):2259–79.

47. Pfadenhauer LM, Gerhardus A, Mozygemba K, et al. Making sense of complexity in context and implementation: The Context and Implementation of Complex Interventions (CICI) framework. Implement Sci. 2017;12(1):1–17.

48. McCrabb S, Lane C, Hall A, et al. Scaling-up evidence-based obesity interventions: A systematic review assessing intervention adaptations and effectiveness and quantifying the scale-up penalty. Obes Rev. 2019;(December 2018):964–82.

For peer review only

For peer review only - http://bmjopen.bmj.com/site/about/guidelines.xhtml

Tables included in the text

Table 1 Characteristics of the included studies

Author, year, study type	Participants eligible / targeted	Participants randomized (intervention, control)[a]	Intervention duration	Time of the follow-up measurements	Participants with valid data in the measurements	Change in mean zBMI 1st or 2nd measurement vs baseline (SD / CI 95%)	Change in mean zBMI vs control (SD / CI 95%)	When evaluated
Hodgkinson et al., (2019), Pragmatic CRT	2-4 years, Mean age=NR	87 (48, 39)	6 months	2-years post baseline	81 (47, 34)	-0.18* (SD 0.99)	-0.74* (-1.10 to -0.38)	NR (Trial published in 2019)
Malden et al., (2019), Feasibility CRT	3-5 years, Mean age=4.4	42 (26, 16)	18 weeks	15th-17th week of the intervention	36 (22, 14)	+0.02 (-0.11 to 0.15)	-0.04 (SD 0.13)	2018
Bryant et al., (2021), Feasibility CRT	6 months - 5 years Mean age=NR	117 (47, 70)	8 weeks	11-months post baseline	99 (39, 60)	-0.9 (SD 0.46)	-1.4 (SD 0.08)	NR (Trial published in 2021)
Lanigan et al., (Unpublished) RCT – trial 1	1-5 years, with OW/OB/high risk Mean age=2.4	88 (49, 39)	6 months	6-months post baseline	64 (36, 28)	-0.2 (-0.3 to -0.03)	-0.2 (-0.5 to 0.05)	(Trial not yet published)
				24 months after the end of the intervention	42	-0.3 * (-0.5, -0.1),	NR	(Trial not yet published)
Lanigan et al., (Unpublished) RCT -trial 2	1-5 years, of any weight status. Mean age=2.1	85 (38, 47)	6 months	6-months post baseline	64 (27, 37)	-0.02 (-0.2 to 0.2),	-0.09 (-0.4 to 0.2)	(Trial not yet published)
				12 months after the end of the intervention	39	-0.3 (-0.7, 0.1),	NR	(Trial not yet published)

For peer review only - http://bmjopen.bmj.com/site/about/guidelines.xhtml

Tee et al., (2021), Impact Assessment	Children aged ≤6 years with severe OB Mean age=4.1	147	12 months	12 months post baseline	41	-0.5*	NR	2012-17

CRT, cluster randomised trial, NR, not reported, NA, not applicable, RCT, randomised controlled trial, OW, overweight, OB, obesity
[a]: Numbers at randomisation, *: significant change in zBMI

For peer review only

For peer review only - http://bmjopen.bmj.com/site/about/guidelines.xhtml

Figure legends

Figure 1 Flowchart of the included studies

For peer review only

For peer review only - http://bmjopen.bmj.com/site/about/guidelines.xhtml

For peer review only

For peer review only - http://bmjopen.bmj.com/site/about/guidelines.xhtml

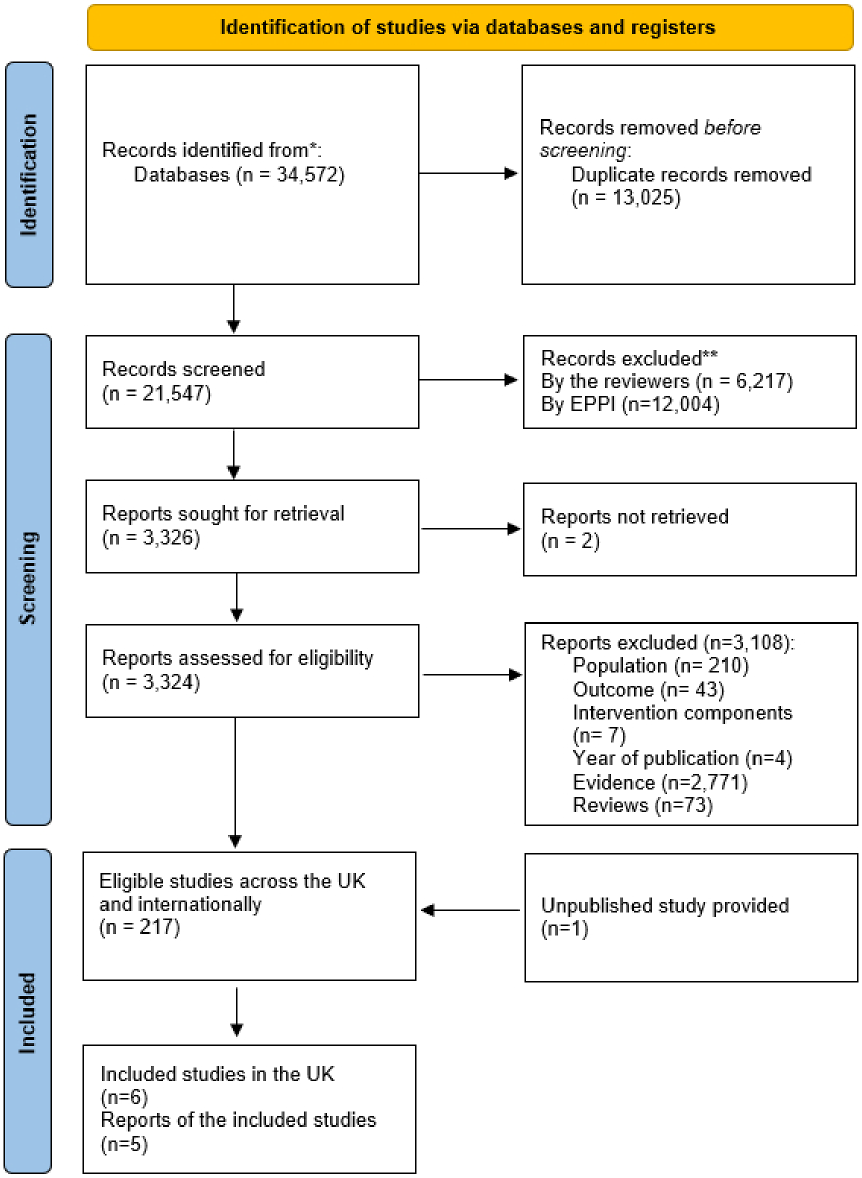

Figure 1 Flowchart of the included studies

408x528mm (38 x 38 DPI)

Supplementary Material

Appendix 1 – Search terms and search strategy

The following databases were searched in November 2021. The searches were updated in March 2023.

1. Pubmed/Medline
2. Cochrane Library Web
3. CINAHL
4. Scopus
5. PsycINFO
6. Web of Science
7. Social Policy and Practice
8. Health Management Information Consortium (HMIC)
9. Trials Register of Promoting Health Interventions (TRoPHI)

The indicative search terms that were used are presented on the table below:

Table S1: Search terms

Concept 1<br>Participants	Concept 2<br>Exposure	Concept 3<br>Outcomes
Subject headings:<br>"Child, preschool" OR<br>"Schools, nursery" OR<br>"Child day care centers"	(("diet" OR "food" OR "beverages" OR "food quality" OR "food preferences" OR "feeding behavior*")<br>OR<br>("exercise" OR "sedentary behavior" " OR "physical fitness"))<br>AND<br>("weight reduction programs" OR "health education" OR "health promotion" OR "primary prevention" OR "pilot projects" OR "feasibility studies" OR "program evaluation")	("obesity" OR "body mass index" OR "body weight" OR "body size" OR "body composition" OR "body weight changes" OR

For peer review only

Keywords<br><br>"child preschool" OR "preschool*" OR "preschool*" OR "pre school*" OR "kindergarten" OR "kindergarden" OR "prekinder*" OR "pre kinder*" OR "pre kinder*" OR "headstart" OR "head start" OR "sure start" OR "surestart" OR "family child care home*" OR "family childcare home*" OR "infant*" OR "toddler*" OR "childcare" OR "child-care" OR "child-care" OR "early learning center*" OR "early learning centre*" OR "early child*" OR "early care and education" OR ("child*" AND ("day-care" OR "day-care" OR "daycare")) OR "playgroup*" OR "play group*" OR "schools nursery" OR (("center" OR "center s" OR "centers"OR "centre" OR "centre s" OR "centres") AND "child, preschool") OR "child day care centers" OR "child day care centers")	(("diet" OR "dietary" OR "food" OR "beverages"OR "snack*" OR "fruit*" OR "vegetable*" OR OR "food quality" OR "drink*" OR "juice*" OR "nutrition*" OR "food habit*" OR "food choice*" OR "overeat*" OR "over eat*" OR "weight control" OR "weight management" OR "menu planning" OR "menu choice*" OR "meal choice*" OR "meal*" OR "meal planning" OR ("food" AND "consum*") OR "food preferences" OR "feeding behavior*" OR "feeding behaviour*" OR "energy density" OR "lifestyle behaviour" OR "eating behaviour" OR "healthy eating" OR "health promotion" OR "eating habit*")<br><br>OR<br><br>("exercise" OR "sedentary behaviour OR "physical fitness" OR "movement" OR "playtime"OR "play*" OR "sport*"))<br><br>AND<br><br>("intervention*" OR "program*" OR "programme*" OR "project*" OR "therapeutics" OR "scheme" OR "initiative*" OR "strateg*" OR "service*" OR "primary prevention" OR "health education" OR "health promotion" OR "primary prevention" OR "treat*" OR "therapy*" OR "pilot projects" OR "feasibility studies" OR "program evaluation")	"overweight")("obesity" OR "obes*" OR "body mass index" OR "BMI" OR "bmi z score" OR "bmi z score" OR "body mass index z-score" OR "body mass index z-score" OR "bmi percentile*" OR "bmi centile*" OR "body mass index percentile*" OR "body mass index centile*" OR "adipos*" OR "overweight" OR "healthy weight" OR "weight loss" OR "weight gain")

For peer review only

Search strategy in all databases:

PubMed

#1 Child/setting terms	("child, preschool"[MeSH Terms] OR "child preschool"[Title/Abstract] OR "preschool*"[Title/Abstract] OR "preschool*"[Title/Abstract] OR "pre school*"[Title/Abstract] OR "kindergarten"[Title/Abstract] OR "kindergarden"[Title/Abstract] OR "prekinder*"[Title/Abstract] OR "pre kinder*"[Title/Abstract] OR "pre kinder*"[Title/Abstract] OR "headstart"[Title/Abstract] OR "head start"[Title/Abstract] OR "sure start"[Title/Abstract] OR "surestart"[Title/Abstract] OR "family child care home*"[Title/Abstract] OR "family childcare home*"[Title/Abstract] OR "infant*"[Title/Abstract] OR "toddler*"[Title/Abstract] OR "childcare"[Title/Abstract] OR "child-care"[Title/Abstract] OR "child-care"[Title/Abstract] OR "early learning center*"[Title/Abstract] OR "early learning centre*"[Title/Abstract] OR "early child*"[Title/Abstract] OR "early care and education"[Title/Abstract] OR ("child*"[Title/Abstract] AND ("day-care"[Title/Abstract] OR "day-care"[Title/Abstract] OR "daycare"[Title/Abstract])) OR "playgroup*"[Title/Abstract] OR "play group*"[Title/Abstract] OR "schools, nursery"[MeSH Terms] OR "schools nursery"[Title/Abstract] OR (("center"[Title/Abstract] OR "center s"[Title/Abstract] OR "centers"[Title/Abstract] OR "centre"[Title/Abstract] OR "centre s"[Title/Abstract] OR "centres"[Title/Abstract]) AND "child, preschool"[MeSH Terms]) OR "child day care centers"[MeSH Terms] OR "child day care centers"[Title/Abstract])
#2 Diet terms	("diet"[MeSH Terms] OR "diet"[Title/Abstract] OR "dietary"[Title/Abstract] OR "food"[MeSH Terms] OR "food"[Title/Abstract] OR "beverages"[MeSH Terms] OR "beverages"[Title/Abstract] OR "snack*"[Title/Abstract] OR "fruit*"[Title/Abstract] OR "vegetable*"[Title/Abstract] OR "food quality"[MeSH Terms] OR "food quality"[Title/Abstract] OR "drink*"[Title/Abstract] OR "juice*"[Title/Abstract] OR "nutrition*"[Title/Abstract] OR "food habit*"[Title/Abstract] OR "food choice*"[Title/Abstract] OR "overeat*"[Title/Abstract] OR "over eat*"[Title/Abstract] OR "weight control"[Title/Abstract] OR "weight management"[Title/Abstract] OR "menu planning"[Title/Abstract] OR "menu choice*"[Title/Abstract] OR "meal choice*"[Title/Abstract] OR "meal*"[Title/Abstract] OR "meal planning"[Title/Abstract] OR ("food"[Title/Abstract] AND "consum*"[Title/Abstract]) OR "food preferences"[MeSH Terms] OR "food preferences"[Title/Abstract] OR "feeding behavior*"[MeSH Terms] OR "feeding behavior*"[Title/Abstract] OR "feeding behaviour*"[Title/Abstract] OR "energy density"[Title/Abstract] OR "lifestyle behaviour"[Title/Abstract] OR "eating behaviour"[Title/Abstract] OR "healthy eating"[Title/Abstract] OR "health promotion"[Title/Abstract] OR "eating habit*"[Title/Abstract])
#3 **PA terms**	("exercise"[MeSH Terms] OR "exercise"[Title/Abstract] OR "sedentary behavior"[MeSH Terms] OR "sedentary behavior"[Title/Abstract] OR "sedentary behaviour"[Title/Abstract] OR "physical fitness"[MeSH Terms] OR "physical fitness"[Title/Abstract] OR "movement"[Title/Abstract] OR "playtime"[Title/Abstract] OR "play*"[Title/Abstract] OR "sport*"[Title/Abstract])
#4 Intervention terms	("intervention*"[Title/Abstract] OR "weight reduction programs"[MeSH Terms] OR "program*"[Title/Abstract] OR "programme*"[Title/Abstract] OR "project*"[Title/Abstract] OR "therapeutics"[Title/Abstract] OR "scheme"[Title/Abstract] OR "initiatives"[Title/Abstract] OR "strateg*"[Title/Abstract] OR "service*"[Title/Abstract] OR "pilot projects"[MeSH Terms] OR "pilot projects"[Title/Abstract] OR "feasibility studies"[MeSH Terms] OR "feasibility studies"[Title/Abstract] OR "program evaluation"[MeSH Terms] OR "program evaluation"[Title/Abstract] OR "health education"[MeSH Terms] OR "health education"[Title/Abstract] OR "health promotion"[MeSH Terms] OR "health promotion"[Title/Abstract] OR "primary prevention"[Title/Abstract] OR "treat*"[Title/Abstract] OR "random*"[Title/Abstract] OR "cluster random*"[Title/Abstract] OR "trial*"[Title/Abstract] OR "evaluat*"[Title/Abstract] OR "effective*"[Title/Abstract] OR "mixed methods"[Title/Abstract] OR "impact*"[Title/Abstract])
#5	("intervention*"[Title/Abstract] OR "weight reduction programs"[MeSH Terms] OR "program*"[Title/Abstract] OR "programme*"[Title/Abstract] OR "project*"[Title/Abstract] OR "therapeutics"[Title/Abstract] OR "scheme"[Title/Abstract] OR "initiative*"[Title/Abstract] OR "strateg*"[Title/Abstract] OR "service*"[Title/Abstract] OR "health education"[MeSH Terms] OR "health promotion"[MeSH Terms] OR "primary prevention"[MeSH Terms] OR "primary prevention"[Title/Abstract] OR "health education"[Title/Abstract] OR "health

For peer review only

	promotion"[Title/Abstract] OR "primary prevention"[Title/Abstract] OR "treat*"[Title/Abstract] OR "therapy*"[Title/Abstract] OR "pilot projects"[MeSH Terms] OR "pilot projects"[Title/Abstract] OR "feasibility studies"[MeSH Terms] OR "feasibility studies"[Title/Abstract] OR "program evaluation"[MeSH Terms] OR "program evaluation"[Title/Abstract])
#6	("random*"[Title/Abstract] OR "cluster random*"[Title/Abstract] OR "trial*"[Title/Abstract] OR "evaluat*"[Title/Abstract] OR "effective*"[Title/Abstract] OR "mixed methods"[Title/Abstract] OR "impact*"[Title/Abstract] OR "non randomized controlled trial*"[Title/Abstract] OR "Pragmatic Clinical Trial"[Title/Abstract] OR "controlled clinical trial*"[Title/Abstract] OR "randomized controlled trial*"[Title/Abstract] OR "randomized"[Title/Abstract] OR "randomised"[Title/Abstract] OR "clinical trial*"[MeSH Terms] OR "clinical trial*"[Title/Abstract] OR "randomly"[Title/Abstract] OR "controlled study"[Title/Abstract] OR "controlled trial*"[Title/Abstract] OR "control group"[Title/Abstract] OR "intervention group"[Title/Abstract] OR "experimental group"[Title/Abstract] OR "comparison group"[Title/Abstract] OR "control school"[Title/Abstract] OR "intervention school"[Title/Abstract] OR "control community"[Title/Abstract] OR "intervention community"[Title/Abstract] OR "intervention condition"[Title/Abstract] OR "control condition"[Title/Abstract] OR "treatment group"[Title/Abstract] OR "control participant"[Title/Abstract] OR "quasi experimental design"[Title/Abstract] OR "experimental condition"[Title/Abstract] OR "treatment condition"[Title/Abstract] OR "pilot projects"[MeSH Terms] OR "feasibility studies"[MeSH Terms] OR "program evaluation"[MeSH Terms] OR "pre test"[Title/Abstract] OR "post test"[Title/Abstract] OR "quasi experiment*"[Title/Abstract] OR "blinded"[Title/Abstract] OR "double bind"[Title/Abstract] OR "fidelity"[Title/Abstract] OR "reach"[Title/Abstract] OR "uptake"[Title/Abstract] OR "implementation"[Title/Abstract] OR "intervention effect*"[Title/Abstract] OR "program effect*"[Title/Abstract] OR "programme effect*"[Title/Abstract] OR "proof of concept*"[Title/Abstract] OR "outcome study"[Title/Abstract])
#7	Search: #5 AND #6
#8	Search: #2 OR #3
#9	Search: #8 AND (#4 OR #7)
**#10**	("obesity"[MeSH Terms] OR "obesity"[Title/Abstract] OR "obes*"[Title/Abstract] OR "body mass index"[MeSH Terms] OR "body mass index"[Title/Abstract] OR "BMI"[Title/Abstract] OR "bmi z score"[Title/Abstract] OR "bmi z score"[Title/Abstract] OR "body mass index z-score"[Title/Abstract] OR "body mass index z-score"[Title/Abstract] OR "bmi percentile*"[Title/Abstract] OR "bmi centile*"[Title/Abstract] OR "body mass index percentile*"[Title/Abstract] OR "body mass index centile*"[Title/Abstract] OR "body weight"[MeSH Terms] OR "body size"[MeSH Terms] OR "adipos*"[Title/Abstract] OR "body composition"[MeSH Terms] OR "body weight changes"[MeSH Terms] OR "overweight"[MeSH Terms] OR "overweight"[Title/Abstract] OR "healthy weight"[Title/Abstract] OR "weight loss"[Title/Abstract] OR "weight gain"[Title/Abstract])
**#11**	Search: #1 AND #9 AND #10

For peer review only

CINAHL

#1<br>Population<br>AB	AB (preschool* OR pre-school* OR “pre-school*” OR kindergar?en OR prekinder* OR pre-Kinder* OR “pre Kinder*” OR headstart OR “head start” OR “surestart” OR “sure start” OR “family child care home” OR “family childcare home” OR “infant*” OR “toddler*” OR “child care” OR “child-care” OR childcare OR “early learning centre” OR “early child*” OR “early care and education” OR (child* AND “day care” OR “daycare” OR “day-care” OR playgroup* OR “nurser*” OR child day care centre*)) **OR** TI ((preschool* OR pre-school* OR “pre-school*” OR kindergar?en OR prekinder* OR pre-Kinder* OR “pre Kinder*” OR headstart OR “head start” OR “surestart” OR “sure start” OR “family child care home” OR “family childcare home” OR “infant*” OR “toddler*” OR “child care” OR “child-care” OR childcare OR “early learning centre” OR “early child*” OR “early care and education” OR (child* AND “day care” OR “daycare” OR “day-care” OR playgroup* OR “nurser*” OR child day care centre*))
#2<br>Diet<br>AB	AB (diet* OR dietary OR food* OR snack* OR fruit* OR drink* OR beverage* OR juice* OR vegetable* OR meal* OR nutrition* OR intake* OR consum* OR “food quality” OR “food preference*” OR “energy density” OR “lifestyle behavio#r” OR “eating behavio#r” OR “healthy eating” OR “feeding behavio#r” OR “health promotion” OR “eating habit*” OR “food habit*” OR “food choice*” OR “overeat*” OR “over eat*” OR “weight control” OR “weight management” OR “menu planning” OR “menu choice” OR “meal choice*” OR “meal*” OR “meal planning” OR (food* AND consum*) OR “eating habit*”) **OR** TI (diet* OR dietary OR food* OR snack* OR fruit* OR drink* OR beverage* OR juice* OR vegetable* OR meal* OR nutrition* OR intake* OR consum* OR “food quality” OR “food preference*” OR “energy density” OR “lifestyle behavio#r” OR “eating behavio#r” OR “healthy eating” OR “feeding behavio#r” OR “health promotion” OR “eating habit*” OR “food habit*” OR “food choice*” OR “overeat*” OR “over eat*” OR “weight control” OR “weight management” OR “menu planning” OR “menu choice” OR “meal choice*” OR “meal*” OR “meal planning” OR (food* AND consum*) OR “eating habit*”)
#3<br>PA<br>AB	AB (“physical* activ*” OR playtime OR sedentary OR exercise* OR play* OR “physical fitness” OR “movement” OR “playtime” OR “sport*”) **OR** TI (“physical* activ*” OR playtime OR sedentary OR exercise* OR play* OR “physical fitness” OR “movement” OR “playtime” OR “sport*”)
#4<br>Interventions<br>AB	AB (“intervention*” OR “weight reduction program*” "program*" OR "programme*" OR "project*" OR "therapeutics" OR "scheme" OR "initiative*" OR "strateg*" OR "life style" OR "service*" OR “pilot project*” OR “feasibility stud*” OR “program evaluation” OR “health education*” OR “health promotion” OR “primary prevention” OR “treat*” OR “random*” OR “cluster random*” OR “trial*” OR “evaluat*” OR “effective*” OR “mixed methods” OR “impact*”) **OR** TI (“intervention*” OR “weight reduction program*” "program*" OR "programme*" OR "project*" OR "therapeutics" OR "scheme" OR "initiative*" OR "strateg*" OR "life style" OR "service*" OR “pilot project*” OR “feasibility stud*” OR “program evaluation” OR “health education*” OR “health promotion” OR “primary prevention” OR “treat*” OR “random*” OR “cluster random*” OR “trial*” OR “evaluat*” OR “effective*” OR “mixed methods” OR “impact*”)
#5<br>Intervention terms	AB ("intervention*" OR "weight reduction programs" OR "program*" OR "programme*" OR "project*" OR "therapeutics" OR "scheme" OR "initiative*" OR "strateg*" OR "service*" OR "health education" OR "health promotion" OR "primary prevention" OR "health education" OR "health promotion" OR "primary prevention" or "treat*" OR therapy* or "pilot projects" OR "feasibility studies" OR "program evaluation") **OR** TI ("intervention*" OR "weight reduction programs" OR "program*" OR "programme*" OR "project*" OR "therapeutics" OR "scheme" OR "initiative*" OR "strateg*" OR "service*" OR "health education" OR "health promotion" OR "primary prevention" OR "health education" OR "health promotion" OR "primary prevention" or "treat*" OR therapy* or "pilot projects" OR "feasibility studies" OR "program evaluation")
#6<br>Evaluati	AB ("random*" OR "cluster random*" OR "trial*" OR "evaluat*" OR "effective*" OR "mixed methods" OR "impact*" OR "Non-Randomized Controlled Trial*" OR "Pragmatic Clinical Trial" OR "controlled Clinical Trial*" OR "randomized controlled trial*" OR “randomized” OR “randomised” OR "clinical trial*" OR “randomly” OR "controlled study" OR "controlled trial*" OR "control group" OR "intervention group" OR "experimental group" OR "comparison group" OR "control school" OR "intervention school" OR "control community" OR "intervention community" OR "intervention condition" OR "control condition" OR "treatment group" OR "control participant" OR "quasi experimental design" OR

on ter ms	"experimental condition" OR "treatment condition" OR "pilot projects" OR "feasibility studies" OR "program evaluation" OR "pre test" OR "post test" OR "quasi experiment*" OR blinded OR "double bind" OR fidelity OR reach OR uptake OR implementation OR "intervention effect*" OR "program effect*" OR "programme effect*" OR "proof of concept*" OR "outcome study") **OR** TI ("random*" OR "cluster random*" OR "trial*" OR "evaluat*" OR "effective*" OR "mixed methods" OR "impact*" OR "Non-Randomized Controlled Trial*" OR "Pragmatic Clinical Trial" OR "controlled Clinical Trial*" OR "randomized controlled trial*" OR “randomized” OR “randomised” OR "clinical trial*" OR “randomly” OR "controlled study" OR "controlled trial*" OR "control group" OR "intervention group" OR "experimental group" OR "comparison group" OR "control school" OR "intervention school" OR "control community" OR "intervention community" OR "intervention condition" OR "control condition" OR "treatment group" OR "control participant" OR "quasi experimental design" OR "experimental condition" OR "treatment condition" OR "pilot projects" OR "feasibility studies" OR "program evaluation" OR "pre test" OR "post test" OR "quasi experiment*" OR blinded OR "double bind" OR fidelity OR reach OR uptake OR implementation OR "intervention effect*" OR "program effect*" OR "programme effect*" OR "proof of concept*" OR "outcome study")
#7	S5 AND S6
#8	S2 OR S3
#9	S4 OR S7
#10	S8 AND S9<br><br>**Limiters** - Age Groups: Infant, 1-23 months, Child, Preschool 2-5 years
#11	AB ("obesity” OR “obes*” OR "body mass index" OR "BMI" OR "bmi z score" OR "bmi z score" OR "body mass index z-score" OR "body mass index z-score" OR "bmi percentile*" OR "bmi centile*" OR "body mass index percentile*" OR "body mass index centile*" OR "body weight" OR "body size" OR "weight*" OR "waist" OR "adipos*" OR "body composition" OR "overweight" OR “body weight changes” OR “healthy weight” OR “weight loss” OR “weight gain”) **OR** TI ("obesity” OR “obes*” OR "body mass index" OR "BMI" OR "bmi z score" OR "bmi z score" OR "body mass index z-score" OR "body mass index z-score" OR "bmi percentile*" OR "bmi centile*" OR "body mass index percentile*" OR "body mass index centile*" OR "body weight" OR "body size" OR "weight*" OR "waist" OR "adipos*" OR "body composition" OR "overweight" OR “body weight changes” OR “healthy weight” OR “weight loss” OR “weight gain”)<br><br>**Limiters** - Age Groups: Infant, 1-23 months, Child, Preschool 2-5 years
#12	#1 AND #10 AND #11<br><br>**Limiters** - Publication Year: 2011-2023

For peer review only

Cochrane

#1<br><br>Population<br><br>TI-AB-KW	(preschool* OR pre-school* OR "pre-school*" OR kindergar?en OR prekinder* OR pre-Kinder* OR "pre Kinder*" OR headstart OR "head start" OR "surestart" OR "sure start" OR "family child care home" OR "family childcare home" OR "infant*" OR "toddler*" OR "child care" OR "child-care" OR childcare OR "early learning centre" OR "early child*" OR "early care and education" OR (child* AND "day care" OR "daycare" OR "day-care" OR playgroup* OR "nurser*" OR child day care centre*))
#2<br><br>Diet<br><br>TI-AB-KW	(diet* OR dietary OR food* OR snack* OR fruit* OR drink* OR beverage* OR juice* OR vegetable* OR meal* OR nutrition* OR intake* OR consum* OR "food quality" OR "food preference*" OR "energy density" OR "lifestyle behavio*r" OR "eating behavio*r" OR "healthy eating" OR "feeding behavio*r" OR "health promotion" OR "eating habit*" OR "food habit*" OR "food choice*" OR "overeat*" OR "over eat*" OR "weight control" OR "weight management" OR "menu planning" OR "menu choice*" OR "meal choice*" OR "meal*" OR "meal planning" OR (food* AND consum*) OR "eating habit*")
#3<br><br>PA<br><br>TI-AB-KW	("physical* activ*" OR sport OR sports OR exercis* OR playtime OR sedentary OR exercise* OR play* OR "physical fitness" OR "movement" OR "playtime")
#4<br><br>Outcome terms<br><br>TI-AB-KW	(obesity OR obes* OR "body mass index" OR BMI OR "bmi z score" OR "bmi z score" OR "body mass index z-score" OR "body mass index z-score" OR "bmi percentile*" OR "bmi centile*" OR "body mass index percentile*" OR "body mass index centile*" OR "body weight" OR "body size" OR weight* OR waist OR "adipos*" OR "body composition" OR overweight OR "body weight changes" OR "healthy weight" OR "weight loss" OR "weight gain")
#5	#1 AND #6 AND #7
#6	("intervention*" OR "weight reduction programs" OR "program*" OR "programme*" OR "project*" OR "therapeutics" OR "scheme" OR "initiative*" OR "strateg*" OR "service*" OR "health education" OR "health promotion" OR "primary prevention" OR "health education" OR "health promotion" OR "primary prevention" or "treat*" OR therapy* or "pilot projects" OR "feasibility studies" OR "program evaluation")
#7	#5 AND #6

For peer review only

Web of Science

#1<br>Population<br>TS	TS=(preschool* OR pre-school* OR “pre-school*” OR kindergar$en OR prekinder* OR pre-Kinder* OR “pre Kinder*” OR headstart OR “head start” OR “surestart” OR “sure start” OR “family child care home” OR “family childcare home” OR “infant*” OR “toddler*” OR “child care” OR “child-care” OR childcare OR “early learning centre” OR “early child*” OR “early care and education” OR (child* AND “day care” OR “daycare” OR “day-care” OR playgroup* OR “nurser*” OR “child day care centre*”))
#2<br>Diet<br>TS	TS=((diet* OR dietary OR food* OR snack* OR fruit* OR drink* OR beverage* OR juice* OR vegetable* OR meal* OR nutrition* OR intake* OR consum* OR “food quality” OR “food preference*” OR “energy density” OR “lifestyle behavio$r” OR “eating behavio$r” OR “healthy eating” OR “feeding behavio$r” OR “health promotion” OR “eating habit*” OR “food habit*” OR “food choice*” OR “overeat*” OR “over eat*” OR “weight control” OR “menu planning” OR “menu choice*” OR “meal choice*” OR “meal*” OR “meal planning” OR (food* AND consum*) OR “eating habit*”))
#3<br>PA<br>TS	TS=((“physical* activ*” OR playtime OR sedentary OR exercise* OR play* OR “physical fitness” OR “movement” OR “playtime” OR sport*))
#4<br>Interventio<br>ns<br>TS	TS=((“intervention*” OR “weight reduction program*” "program*" OR "programme*" OR "project*" OR "therapeutics" OR "scheme" OR "initiative*" OR "strateg*" OR "life style" OR "service*" OR “pilot project*” OR “feasibility stud*” OR “program evaluation” OR “health education*” OR “health promotion” OR “primary prevention” OR “treat*” OR “random*” OR “cluster random*” OR “trial*” OR “evaluat*” OR “effective*” OR “mixed methods” OR “impact*”))
#5	TS=(("intervention*" OR "weight reduction programs" OR "program*" OR "programme*" OR "project*" OR "therapeutics" OR "scheme" OR "initiative*" OR "strateg*" OR "service*" OR "health education" OR "health promotion" OR "primary prevention" OR "health education" OR "health promotion" OR "primary prevention" or "treat*" OR therapy* or "pilot projects" OR "feasibility studies" OR "program evaluation"))
#6	TS=(("random*" OR "cluster random*" OR "trial*" OR "evaluat*" OR "effective*" OR "mixed methods" OR "impact*" OR "Non-Randomized Controlled Trial*" OR "Pragmatic Clinical Trial" OR "controlled Clinical Trial*" OR "randomized controlled trial*" OR “randomized” OR “randomised” OR "clinical trial*" OR “randomly” OR "controlled study" OR "controlled trial*" OR "control group" OR "intervention group" OR "experimental group" OR "comparison group" OR "control school" OR "intervention school" OR "control community" OR "intervention community" OR "intervention condition" OR "control condition" OR "treatment group" OR "control participant" OR "quasi experimental design" OR "experimental condition" OR "treatment condition" OR "pilot projects" OR "feasibility studies" OR "program evaluation" OR "pre test" OR "post test" OR "quasi experiment*" OR blinded OR "double bind" OR fidelity OR reach OR uptake OR implementation OR "intervention effect*" OR "program effect*" OR "programme effect*" OR "proof of concept*" OR "outcome study"))
#7	#5 AND #6
#8	#2 OR #3
#9	#4 OR #7
#10	#8 AND #9

For peer review only

#11	TS=(("obesity" OR "obes*" OR "body mass index" OR "BMI" OR "bmi z score" OR "bmi z score" OR "body mass index z-score" OR "body mass index z-score" OR "bmi percentile*" OR "bmi centile*" OR "body mass index percentile*" OR "body mass index centile*" OR "body weight" OR "body size" OR "weight*" OR "waist" OR "adipos*" OR "body composition" OR "overweight" OR "body weight changes" OR "healthy weight" OR "weight loss" OR "weight gain"))
#12	#1 AND #10 AND #11<br>and 2023 and 2022<br>and 2021 or 2020 or 2019 or 2018 or 2017 or 2016 or 2015 or 2014 or 2013 or 2012 or 2011 (Publication Years)

For peer review only

SCOPUS

1	(TITLE-ABS ((preschool* OR pre-school* OR {pre-school*} OR kindergar?en OR prekinder* OR pre kinder* OR {pre Kinder} OR headstart OR {head start} OR surestart OR {sure start} OR {family child care home} OR {family childcare home} OR infant* OR toddler* OR {child care} OR {child-care} OR childcare OR {early learning centre} OR {early child} OR {early care and education} OR (child* AND {day care} OR daycare OR {day-care}) OR playgroup* OR nurser* OR {child day care centre*}))) AND (((TITLE-ABS ((diet* OR dietary OR food* OR snack* OR fruit* OR drink* OR beverage* OR juice* OR vegetable* OR m eal* OR nutrition* OR intake* OR consum* OR {food quality} OR {food preference} OR {energy density} OR {lifestyle behavio?r} OR {eating behavio?r} OR {healthy eating} OR {feeding behavio?r} OR {health promotion} OR {eating habit*} OR {food habit*} OR {food choice*} OR {overeat*} OR {over eat*} OR {weight control} OR {menu planning} OR {menu choice} OR {meal choice} OR {meal*} OR {meal planning} OR {eating habit*}))) OR (TITLE-ABS (({physical* activ*} OR playtime OR sedentary OR exercise* OR play* OR {physical fitness} OR movement OR playtime OR sport*)))) AND ((TITLE-ABS (intervention* OR {weight reduction program*} OR program* OR programme* OR project* OR therapeutics OR scheme OR initiative OR strateg* OR lifestyle OR service* OR {pilot project*} OR {feasibility stud*} OR {program evaluation} OR {health education*} OR {health promotion} OR "treat*" OR {primary prevention} OR "random*" OR {cluster random*} OR trial* OR evaluat* OR effective* OR {mixed methods} OR impact*)) OR ((TITLE-ABS (intervention* OR {weight reduction programs} OR program* OR programme* OR project* OR therapeutics OR scheme OR initiative* OR strateg* OR service* OR {health education} OR {health promotion} OR {primary prevention} OR {health education} OR treat* OR therapy* OR {pilot projects} OR {feasibility studies} OR {program evaluation})) AND (TITLE-ABS (random* OR {cluster random*} OR trial* OR evaluat* OR effective* OR {mixed methods} OR impact* OR {Non-Randomized Controlled Trial*} OR {Pragmatic Clinical Trial} OR {controlled Clinical Trial*} OR {randomized controlled trial*} OR randomized OR randomised OR {clinical trial*} OR randomly OR {controlled study} OR {controlled trial*} OR {control group} OR {intervention group} OR {experimental group} OR {comparison group} OR {control school} OR {intervention school} OR {control community} OR {intervention community} OR {intervention condition} OR {control condition} OR {treatment group} OR {control participant} OR {quasi experimental design} OR {experimental condition} OR {treatment condition} OR {pilot projects} OR {feasibility studies} OR {program evaluation} OR {pre test} OR {post test} OR {quasi experiment*} OR blinded OR {double blind} OR fidelity OR reach OR uptake OR implementation OR {intervention effect*} OR {program effect*} OR {programme effect*} OR {proof of concept*} OR {outcome study}))))) AND (TITLE-ABS ((obesity OR obes* OR {body mass index} OR {BMI} OR {bmi z score} OR {bmi z score} OR {body mass index z-score} OR {body mass index z-score} OR {bmi percentile*} OR {bmi centile*} OR {body mass index percentile*} OR {body mass index centile*} OR {body weight} OR {body size} OR weight* OR waist OR adipos* OR {body composition} OR overweight OR {body weight changes} OR {healthy weight} OR {weight loss} OR {weight gain}))) AND (LIMIT-TO (PUBYEAR , 2023) OR LIMIT-TO (PUBYEAR , 2022) OR LIMIT-TO (PUBYEAR , 2021) OR LIMIT-TO (PUBYEAR , 2020) OR LIMIT-TO (PUBYEAR , 2019) OR LIMIT-TO (PUBYEAR , 2018) OR LIMIT-TO (PUBYEAR , 2017) OR LIMIT-TO (PUBYEAR , 2016) OR LIMIT-TO (PUBYEAR , 2015) OR LIMIT-TO (PUBYEAR , 2014) OR LIMIT-TO (PUBYEAR , 2013) OR LIMIT-TO (PUBYEAR , 2012) OR LIMIT-TO (PUBYEAR , 2011))

PsychINFO

**1**	(((preschool* or pre-school* or pre-school* or kindergar$en or prekinder* or pre-Kinder* or pre Kinder* or headstart or head start or surestart or sure start or family child care home or family childcare home or infant* or toddler* or child care or child-care or childcare or early learning centre or early child* or early care) and education) or ((child* and day care) or daycare or day-care or playgroup* or nurser* or child day care centre*)).mp. [mp=title, abstract, heading word, table of contents, key concepts, original title, tests & measures, mesh]
**2**	(diet* or dietary or food* or snack* or fruit* or drink* or beverage* or juice* or vegetable* or meal* or nutrition* or intake* or consum* or food quality or food preference* or energy density or lifestyle behavio?r or eating behavio?r or healthy eating or feeding behaviour?r or health promotion or eating habit* or food habit* or food choice* or overeat* or over eat* or weight control or menu planning or menu choice or meal choice* or meal* or meal planning or (food* and consum*) or eating habit*).mp. [mp=title, abstract, heading word, table of contents, key concepts, original title, tests & measures, mesh]
**3**	(physical* activ* or playtime or sedentary or exercise* or play* or physical fitness or movement or playtime or sport*).mp. [mp=title, abstract, heading word, table of contents, key concepts, original title, tests & measures, mesh]
**4**	(intervention* or weight reduction program* or program* or programme* or project* or therapeutics or scheme or initiative* or strateg* or life style or service* or pilot project* or feasibility stud* or program evaluation or health education* or health promotion or primary prevention or treat* or random* or cluster random* or trial* or evaluat* or effective* or mixed methods or impact*).mp. [mp=title, abstract, heading word, table of contents, key concepts, original title, tests & measures, mesh]
**5**	(intervention* or weight reduction programs or program*OR programme* or project* or therapeutics or scheme or initiative* or strateg* or service* or health education or health promotion or primary prevention or health education or health promotion or primary prevention or treat* or therapy* or pilot projects or feasibility studies or program evaluation).mp. [mp=title, abstract, heading word, table of contents, key concepts, original title, tests & measures, mesh]
**6**	(random* or cluster random* or trial* or evaluat* or effective* or mixed methods or impact* or Non-Randomized Controlled Trial* or Pragmatic Clinical Trial or controlled Clinical Trial* or randomized controlled trial* or randomized or randomised or clinical trial* or randomly or controlled study or controlled trial* or control group or intervention group or experimental group or comparison group or control school or intervention school or control community or intervention community or intervention condition or control condition or treatment group or control participant or quasi experimental design or experimental condition or treatment condition or pilot projects or feasibility studies or program evaluation or pre test or post test or quasi experiment* or blinded or double blind or fidelity or reach or uptake or implementation or intervention effect* or program effect* or programme effect* or proof of concept* or outcome study).mp. [mp=title, abstract, heading word, table of contents, key concepts, original title, tests & measures, mesh]
**7**	5 and 6
**8**	2 or 3
**9**	4 or 7
**10**	8 and 9
**11**	(obesity or obes* or body mass index or BMI or bmi z score or bmi z score or body mass index z-score or body mass index z-score or bmi percentile* or bmi centile* or body mass index percentile* or body mass index centile* or body weight or body size or weight* or waist or adipos* or body composition or overweight or body weight changes or healthy weight or weight loss or weight gain).mp. [mp=title, abstract, heading word, table of contents, key concepts, original title, tests & measures, mesh]
**12**	1 and 10 and 11

For peer review only

**13**	limit 12 to last 12 years

For peer review only

Social Policy and Practice

**1**	(((preschool* or pre-school* or pre-school* or kindergar$en or prekinder* or pre-Kinder* or pre Kinder* or headstart or head start or surestart or sure start or family child care home or family childcare home or infant* or toddler* or child care or child-care or childcare or early learning centre or early child* or early care) and education) or ((child* and day care) or daycare or day-care or playgroup* or nurser* or child day care centre*)).mp. [mp=abstract, title, publication type, heading word, accession number]
**2**	(diet* or dietary or food* or snack* or fruit* or drink* or beverage* or juice* or vegetable* or meal* or nutrition* or intake* or consum* or food quality or food preference* or energy density or lifestyle behavio?r or eating behavio?r or healthy eating or feeding behaviour?r or health promotion or eating habit* or food habit* or food choice* or overeat* or over eat* or weight control or menu planning or menu choice or meal choice* or meal* or meal planning or (food* and consum*) or eating habit*).mp. [mp=abstract, title, publication type, heading word, accession number]
**3**	(physical* activ* or playtime or sedentary or exercise* or play* or physical fitness or movement or playtime or sport*).mp. [mp=abstract, title, publication type, heading word, accession number]
**4**	(intervention* or weight reduction program* or program* or programme* or project* or therapeutics or scheme or initiative* or strateg* or life style or service* or pilot project* or feasibility stud* or program evaluation or health education* or health promotion or primary prevention or treat* or random* or cluster random* or trial* or evaluat* or effective* or mixed methods or impact*).mp. [mp=abstract, title, publication type, heading word, accession number]
**5**	(intervention* or weight reduction programs or program*OR programme* or project* or therapeutics or scheme or initiative* or strateg* or service* or health education or health promotion or primary prevention or health education or health promotion or primary prevention or treat* or therapy* or pilot projects or feasibility studies or program evaluation).mp. [mp=abstract, title, publication type, heading word, accession number]
**6**	(random* or cluster random* or trial* or evaluat* or effective* or mixed methods or impact* or Non-Randomized Controlled Trial* or Pragmatic Clinical Trial or controlled Clinical Trial* or randomized controlled trial* or randomized or randomised or clinical trial* or randomly or controlled study or controlled trial* or control group or intervention group or experimental group or comparison group or control school or intervention school or control community or intervention community or intervention condition or control condition or treatment group or control participant or quasi experimental design or experimental condition or treatment condition or pilot projects or feasibility studies or program evaluation or pre test or post test or quasi experiment* or blinded or double blind or fidelity or reach or uptake or implementation or intervention effect* or program effect* or programme effect* or proof of concept* or outcome study).mp. [mp=abstract, title, publication type, heading word, accession number]
**7**	5 and 6
**8**	2 or 3
**9**	4 or 7
**10**	8 and 9
**11**	(obesity or obes* or body mass index or BMI or bmi z score or bmi z score or body mass index z-score or body mass index z-score or bmi percentile* or bmi centile* or body mass index percentile* or body mass index centile* or body weight or body size or weight* or waist or adipos* or body composition or overweight or body weight changes or healthy weight or weight loss or weight gain).mp. [mp=abstract, title, publication type, heading word, accession number]
**12**	1 and 10 and 11
**13**	limit 12 to last 12 years

HMIC Health Management Information Consortium

**1**	(((preschool* or pre-school* or pre-school* or kindergar$en or prekinder* or pre-Kinder* or pre Kinder* or headstart or head start or surestart or sure start or family child care home or family childcare home or infant* or toddler* or child care or child-care or childcare or early learning centre or early child* or early care) and education) or ((child* and day care) or daycare or day-care or playgroup* or nurser* or child day care centre*)).mp. [mp=title, other title, abstract, heading words]
**2**	(diet* or dietary or food* or snack* or fruit* or drink* or beverage* or juice* or vegetable* or meal* or nutrition* or intake* or consum* or food quality or food preference* or energy density or lifestyle behavio?r or eating behavio?r or healthy eating or feeding behaviour?r or health promotion or eating habit* or food habit* or food choice* or overeat* or over eat* or weight control or menu planning or menu choice or meal choice* or meal* or meal planning or (food* and consum*) or eating habit*).mp. [mp=title, other title, abstract, heading words]
**3**	(physical* activ* or playtime or sedentary or exercise* or play* or physical fitness or movement or playtime or sport*).mp. [mp=title, other title, abstract, heading words]
**4**	(intervention* or weight reduction program* or program* or programme* or project* or therapeutics or scheme or initiative* or strateg* or life style or service* or pilot project* or feasibility stud* or program evaluation or health education* or health promotion or primary prevention or treat* or random* or cluster random* or trial* or evaluat* or effective* or mixed methods or impact*).mp. [mp=title, other title, abstract, heading words]
**5**	(intervention* or weight reduction programs or program*OR programme* or project* or therapeutics or scheme or initiative* or strateg* or service* or health education or health promotion or primary prevention or health education or health promotion or primary prevention or treat* or therapy* or pilot projects or feasibility studies or program evaluation).mp. [mp=title, other title, abstract, heading words]
**6**	(random* or cluster random* or trial* or evaluat* or effective* or mixed methods or impact* or Non-Randomized Controlled Trial* or Pragmatic Clinical Trial or controlled Clinical Trial* or randomized controlled trial* or randomized or randomised or clinical trial* or randomly or controlled study or controlled trial* or control group or intervention group or experimental group or comparison group or control school or intervention school or control community or intervention community or intervention condition or control condition or treatment group or control participant or quasi experimental design or experimental condition or treatment condition or pilot projects or feasibility studies or program evaluation or pre test or post test or quasi experiment* or blinded or double blind or fidelity or reach or uptake or implementation or intervention effect* or program effect* or programme effect* or proof of concept* or outcome study).mp. [mp=title, other title, abstract, heading words]
**7**	5 and 6
**8**	2 or 3
**9**	4 or 7
**10**	8 and 9
**11**	(obesity or obes* or body mass index or BMI or bmi z score or bmi z score or body mass index z-score or body mass index z-score or bmi percentile* or bmi centile* or body mass index percentile* or body mass index centile* or body weight or body size or weight* or waist or adipos* or body composition or overweight or body weight changes or healthy weight or weight loss or weight gain).mp. [mp=title, other title, abstract, heading words]
**12**	1 and 10 and 11
**13**	limit 12 to last 12 years

For peer review only

Trials Register of Promoting Health Interventions (TRoPHI)

saved 551 records from the following searches:

Search 1:

2343: 2341 AND 2342 361 records (of which saved records from year 2011 onwards)

2342: Coded with: children (0-10 yrs) 1933

2341: Coded with: obesity 1194

Search 2:

2366: 2365 AND 2329 (unscreened records): 123 records

2365: 2364 AND 2347 1116

2347: "toddler* OR young child* OR preschool* OR infant* OR child*" (in Title and Abstract)

2364: Coded with: Weight Loss, OR Physical Activity

Search 3:

2344: AND 2347 AND 2355 AND 2338: 221 records

2344: Not coded with: EPPI-Centre Health promotion keywording strategy

2347: "toddler* OR young child* OR preschool* OR infant* OR child*" (in Title and Abstract)

2355: "obes* OR sedentar* OR physical* OR diet* OR exercise OR BMI OR "body weight" OR "body size" OR waist " (in Title and Abstract)

2338: Coded with: Include: Randomised controlled trial, OR Include: Non-Random 14391

Search 4: 136 records: (of which saved records from year 2010 onwards)

"obes* OR sedentar* OR physical* OR diet*" (in Title and Abstract)

AND

"exercise OR BMI OR "body weight" OR "body size" OR waist " (in Title and Abstract)

AND

"toddler* OR young child* OR preschool* OR infant* OR child*" (in Title and Abstract)

AND

Not coded with: EPPI-Centre Health promotion keywording strategy

AND

Not coded with: Ex 1: Not Health Promotion or Public Health, OR Ex 2: Not Human or Environment, OR Ex 3: NOT an evaluation of an intervention, OR Ex: 4 No control or comparison group, OR Ex 5: Item is a review or meta-analysis, OR MARKER: QUERY, OR Duplicate

For peer review only

Appendix 2

Depiction of the screening process

The graph below depicts the screening process on tittle and abstract. The prioritisation of records was refreshing regularly. The most relevant articles were screened first. The screening stopped when the graph reached a plateau.

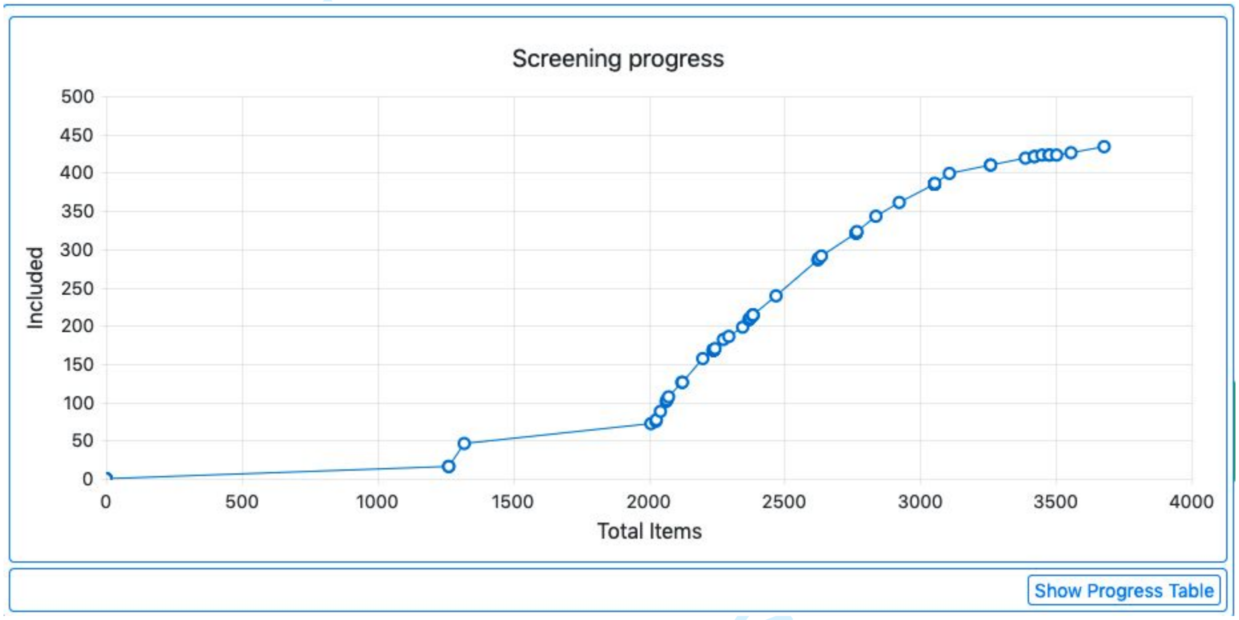

Appendix 3

Bias assessment

Table S2: Studies assessed using the ROB tool

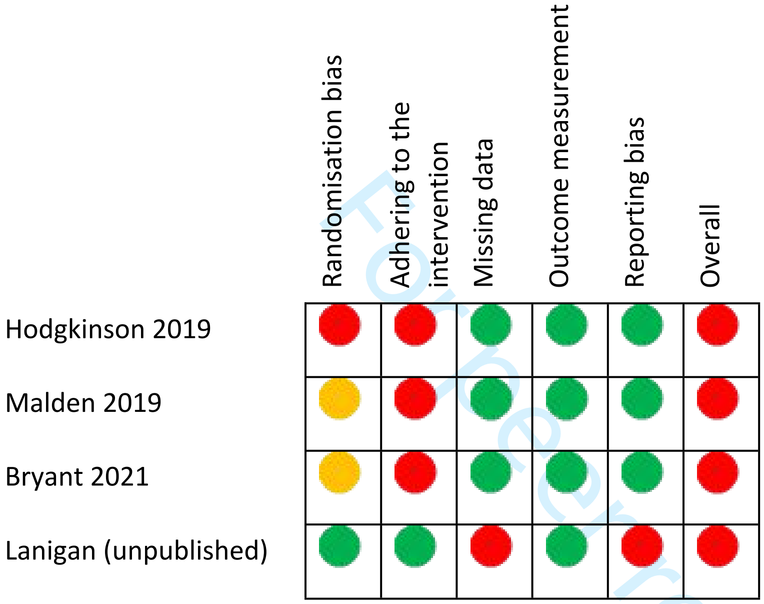

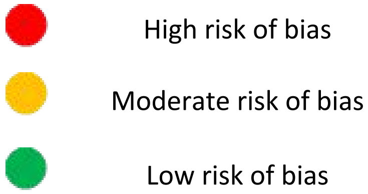

Table S3: Studies assessed using the ROBINS-I tool

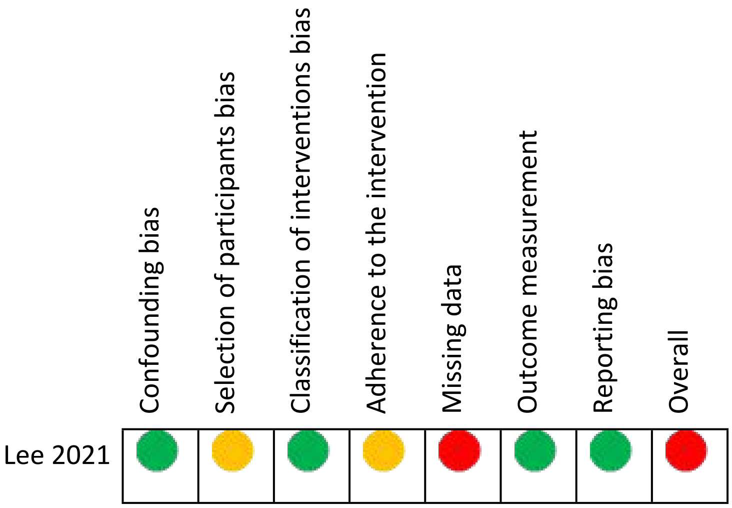

For peer review only

PRISMA 2020 Checklist

Section and Topic	Item #	Checklist item	Location where item is reported
**TITLE**			
Title	1	Identify the report as a systematic review.	Lines 1-2
**ABSTRACT**			
Abstract	2	See the PRISMA 2020 for Abstracts checklist.	Lines 16-40
**INTRODUCTION**			
Rationale	3	Describe the rationale for the review in the context of existing knowledge.	Lines 93-100
Objectives	4	Provide an explicit statement of the objective(s) or question(s) the review addresses.	Lines 72-79
**METHODS**			
Eligibility criteria	5	Specify the inclusion and exclusion criteria for the review and how studies were grouped for the syntheses.	Lines 115-128 & Lines 152-155
Information sources	6	Specify all databases, registers, websites, organisations, reference lists and other sources searched or consulted to identify studies. Specify the date when each source was last searched or consulted.	Lines 106-114 & Appendix 1
Search strategy	7	Present the full search strategies for all databases, registers and websites, including any filters and limits used.	Appendix 1
Selection process	8	Specify the methods used to decide whether a study met the inclusion criteria of the review, including how many reviewers screened each record and each report retrieved, whether they worked independently, and if applicable, details of automation tools used in the process.	Lines 129-147
Data collection process	9	Specify the methods used to collect data from reports, including how many reviewers collected data from each report, whether they worked independently, any processes for obtaining or confirming data from study investigators, and if applicable, details of automation tools used in the process.	Lines 141-147
Data items	10a	List and define all outcomes for which data were sought. Specify whether all results that were compatible with each outcome domain in each study were sought (e.g. for all measures, time points, analyses), and if not, the methods used to decide which results to collect.	Lines 155-162
	10b	List and define all other variables for which data were sought (e.g. participant and intervention characteristics, funding sources). Describe any assumptions made about any missing or unclear information.	Lines 142-147
Study risk of bias assessment	11	Specify the methods used to assess risk of bias in the included studies, including details of the tool(s) used, how many reviewers assessed each study and whether they worked independently, and if applicable, details of automation tools used in the process.	Lines 163-167
Effect measures	12	Specify for each outcome the effect measure(s) (e.g. risk ratio, mean difference) used in the synthesis or presentation of results.	Lines 155-157
Synthesis methods	13a	Describe the processes used to decide which studies were eligible for each synthesis (e.g. tabulating the study intervention characteristics and comparing against the planned groups for each synthesis (item #5)).	Lines 152-157
	13b	Describe any methods required to prepare the data for presentation or synthesis, such as handling of missing summary statistics, or data conversions.	NA
	13c	Describe any methods used to tabulate or visually display results of individual studies and syntheses.	NA
	13d	Describe any methods used to synthesize results and provide a rationale for the choice(s). If meta-analysis was performed, describe the model(s), method(s) to identify the presence and extent of statistical heterogeneity, and software package(s) used.	Lines 152-155
	13e	Describe any methods used to explore possible causes of heterogeneity among study results (e.g. subgroup analysis, meta-regression).	NA
	13f	Describe any sensitivity analyses conducted to assess robustness of the synthesized results.	NA
Reporting bias assessment	14	Describe any methods used to assess risk of bias due to missing results in a synthesis (arising from reporting biases).	NA

For peer review only - http://bmjopen.bmj.com/site/about/guidelines.xhtml

PRISMA 2020 Checklist

Section and Topic	Item #	Checklist item	Location where item is reported
Certainty assessment	15	Describe any methods used to assess certainty (or confidence) in the body of evidence for an outcome.	NA
**RESULTS**			
Study selection	16a	Describe the results of the search and selection process, from the number of records identified in the search to the number of studies included in the review, ideally using a flow diagram.	Lines 177-188 & Figure 1
	16b	Cite studies that might appear to meet the inclusion criteria, but which were excluded, and explain why they were excluded.	Figure 1
Study characteristics	17	Cite each included study and present its characteristics.	Lines 187-200 & Table 1
Risk of bias in studies	18	Present assessments of risk of bias for each included study.	Appendix 3
Results of individual studies	19	For all outcomes, present, for each study: (a) summary statistics for each group (where appropriate) and (b) an effect estimate and its precision (e.g. confidence/credible interval), ideally using structured tables or plots.	Table 1
Results of syntheses	20a	For each synthesis, briefly summarise the characteristics and risk of bias among contributing studies.	Lines 202-258
	20b	Present results of all statistical syntheses conducted. If meta-analysis was done, present for each the summary estimate and its precision (e.g. confidence/credible interval) and measures of statistical heterogeneity. If comparing groups, describe the direction of the effect.	NA
	20c	Present results of all investigations of possible causes of heterogeneity among study results.	NA
	20d	Present results of all sensitivity analyses conducted to assess the robustness of the synthesized results.	NA
Reporting biases	21	Present assessments of risk of bias due to missing results (arising from reporting biases) for each synthesis assessed.	NA
Certainty of evidence	22	Present assessments of certainty (or confidence) in the body of evidence for each outcome assessed.	NA
**DISCUSSION**			
Discussion	23a	Provide a general interpretation of the results in the context of other evidence.	Lines 270-277
	23b	Discuss any limitations of the evidence included in the review.	Lines 282-288 & Lines 311-319
	23c	Discuss any limitations of the review processes used.	Lines 337-344
	23d	Discuss implications of the results for practice, policy, and future research.	Lines 345-353
**OTHER INFORMATION**			
Registration and protocol	24a	Provide registration information for the review, including register name and registration number, or state that the review was not registered.	Lines 102-105
	24b	Indicate where the review protocol can be accessed, or state that a protocol was not prepared.	Lines 102-105
	24c	Describe and explain any amendments to information provided at registration or in the protocol.	NA
Support	25	Describe sources of financial or non-financial support for the review, and the role of the funders or sponsors in the review.	Lines 50-55
Competing interests	26	Declare any competing interests of review authors.	Line 56
Availability of data, code and	27	Report which of the following are publicly available and where they can be found: template data collection forms; data extracted from included studies; data used for all analyses; analytic code; any other materials used in the review.	NA

For peer review only

For peer review only - http://bmjopen.bmj.com/site/about/guidelines.xhtml

PRISMA 2020 Checklist

Section and Topic	Item #	Checklist item	Location where item is reported
other materials			

From: Page MJ, McKenzie JE, Bossuyt PM, Boutron I, Hoffmann TC, Mulrow CD, et al. The PRISMA 2020 statement: an updated guideline for reporting systematic reviews. BMJ 2021;372:n71. doi: 10.1136/bmj.n71

For more information, visit: http://www.prisma-statement.org/

For peer review only

BMJ Open

Assessing the impact of obesity interventions in the early years: A systematic review of UK-based studies

Journal:	*BMJ Open*
Manuscript ID	bmjopen-2023-076479.R1
Article Type:	Original research
Date Submitted by the Author:	05-Dec-2023
Complete List of Authors:	Michalopoulou, Semina; University College London, Great Ormond Street Institute of Child Health<br>Sifaki, Maria; University College London, Great Ormond Street Institute of Child Health<br>Packer, Jessica; University College London, Great Ormond Street Institute of Child Health<br>Lanigan, Julie; University College London, Great Ormond Street Institute of Child Health<br>Stansfield, Claire; University College London, Institute of Education<br>Viner, Russell; University College London, Great Ormond Street Institute of Child Health<br>Russell, Simon; University College London, Great Ormond Street Institute of Child Health
**Primary Subject Heading**:	Paediatrics
Secondary Subject Heading:	Health policy
Keywords:	Obesity, NUTRITION & DIETETICS, Community child health < PAEDIATRICS

SCHOLARONE™
Manuscripts

I, the Submitting Author has the right to grant and does grant on behalf of all authors of the Work (as defined in the below author licence), an exclusive licence and/or a non-exclusive licence for contributions from authors who are: i) UK Crown employees; ii) where BMJ has agreed a CC-BY licence shall apply, and/or iii) in accordance with the terms applicable for US Federal Government officers or employees acting as part of their official duties; on a worldwide, perpetual, irrevocable, royalty-free basis to BMJ Publishing Group Ltd ("BMJ") its licensees and where the relevant Journal is co-owned by BMJ to the co-owners of the Journal, to publish the Work in this journal and any other BMJ products and to exploit all rights, as set out in our licence.

The Submitting Author accepts and understands that any supply made under these terms is made by BMJ to the Submitting Author unless you are acting as an employee on behalf of your employer or a postgraduate student of an affiliated institution which is paying any applicable article publishing charge ("APC") for Open Access articles. Where the Submitting Author wishes to make the Work available on an Open Access basis (and intends to pay the relevant APC), the terms of reuse of such Open Access shall be governed by a Creative Commons licence – details of these licences and which Creative Commons licence will apply to this Work are set out in our licence referred to above.

Other than as permitted in any relevant BMJ Author's Self Archiving Policies, I confirm this Work has not been accepted for publication elsewhere, is not being considered for publication elsewhere and does not duplicate material already published. I confirm all authors consent to publication of this Work and authorise the granting of this licence.

For peer review only

Assessing the impact of obesity interventions in the early years: A systematic review of UK-based studies

Semina Michalopoulou,[1] Maria Sifaki,[1] Jessica Packer,[1] Julie Lanigan,[1] Clare Stansfield,[2] Russell Viner[1] and Simon Russell[1]

[1] UCL Great Ormond Street Institute of Child Health, University College London, London, UK,

[2] UCL Institute of Education, University College London, London, UK

Corresponding author:

Simon Russell

UCL Great Ormond Street Institute of Child Health, University College London, 30 Guilford Street, London, WC1N 1EH

Tel: 0207 905 2110

s.russell@ucl.ac.uk

Short version of the title: UK early childhood obesity interventions

Keywords: childhood obesity, interventions, diet, physical activity, early years, United Kingdom

For peer review only

Abstract

Objectives: Childhood obesity rates in the UK are high. The early years of childhood are critical for establishing healthy behaviours and offer interventional opportunities. We aimed to identify studies evaluating the impact of UK-based obesity interventions in early childhood.

Design: Systematic review using the PRISMA guidelines

Data sources: Nine databases were searched through March 2023 Eligibility criteria: We included UK based obesity intervention studies delivered to children aged 6 months to five years, that had diet and/or physical activity components and reported anthropometric outcomes. The primary outcome of interest was zBMI change (within and between subjects). Studies evaluating the effects of breastfeeding interventions were not included as obesity prevention interventions, given that best practice formula feeding is also likely to encourage healthy growth. The publication date for studies was limited to the previous 12 years (2011-2023), as earlier reviews found few evaluations of interventions in the UK.

Data extraction and synthesis: The reviewers worked independently using standardised approach to search, screen and code the included studies. Risk of bias was assessed using Cochrane tools (ROB 2 or ROBINS-I).

Results: Six trials (five studies) were identified, including two randomised controlled trials (RCT), one cluster randomised trial (CRT), two feasibility CRTs, and one impact assessment. The total number of participants was 566. Three trials focused on disadvantaged families and two included high-risk children categorised as having overweight or obesity. Compared to baseline, five interventions reported reductions in zBMI, three of which were statistically significant ($p<0.05$). Compared to control, five interventions showed zBMI reductions, one of which was

For peer review only

significant. Only two trials were followed up beyond 12 months. All studies were found to have a high risk of bias. Meta-analysis was not possible due to the heterogeneity of studies. Conclusion: UK evidence was limited but some interventions showed promising results in promoting healthy growth. As part of a programme of policies, interventions in the early years may have an important role in reducing the risk of childhood obesity.

PROSPERO registration number: CRD42021290676

Strengths and limitations of this study:

- This is the first systematic review that focuses on childhood obesity interventions among children aged 6 months to 5 years evaluated in the UK.
- Only studies published after 2011 were included.
- Six trials (five studies) were identified after the searches on nine scientific databases. The evidence is limited, however promising effects were found.
- The risk of bias assessment was conducted for all the included studies with tools suitable for the randomised and the non-randomised studies.
- A meta-analysis was not possible due to the heterogeneity of the interventions

Funding statement: This work was supported by the National Institute for Health Research Policy Research Programme (award number: 174868). National Institute for Health Research had no role in the design, analysis or writing of this article. The views expressed in this publication are those of the authors and not necessarily those of the NHS, the National Institute for Health Research, the Department of Health and Social Care or its arm's length bodies, and other Government Departments.

Competing interests statement: None

For peer review only

INTRODUCTION

In England, data from the National Child Measurement Programme (NCMP) show that the prevalence of obesity among children aged 4-5 years, though plateaued, remains stably high. Disadvantaged children are the worst affected, as the inequalities in childhood obesity are increasing.(1,2) Once established, obesity tracks strongly through childhood, adolescence, and into adulthood where it is associated with increased risk of physical and mental health morbidities.(3–7) Prospective evidence indicates that half of the adolescents with obesity had being living with overweight or obesity since the age of five. (8) Data from the NCMP suggest that the odds of children with overweight in the age of 4-5 years developing obesity by 10 and 11 years were 13 times higher compared to children with healthy BMI.(9) Longitudinal evidence shows that a child's weight status remains stable through primary school years, as 68% of children with obesity and 78% of children with healthy weight at 4 and 5 years maintained their weight status by 10 and 11 years.(9) The early years of life are therefore a critical period for the development of a healthy weight, representing a window of opportunity for the introduction of a healthy lifestyle to support healthy growth.

Diet(10,11) and physical activity(12) are the major determinants of weight in young children. The diets of many pre-schoolers in the UK do not adhere to government dietary guidelines, exceeding intake of free sugar and saturated fat, and not reaching recommendations for fruit and vegetables.(13,14) Physical activity levels are reported to be below recommended guidelines(15) with most children not engaging in the 180 minutes of physical activity per day recommended by the National Health Service (NHS).(16) Lifestyle behaviours track through childhood, into adolescence, and adulthood, meaning that those children are likely to continue

For peer review only

following unhealthy practices, thereby being at even greater risk of overweight and obesity.(17,18)

Considering the significance of the first five years for outcomes later in life, interventions to support the establishment of a healthy weight and a healthy lifestyle are valuable. In the UK, the National Institute for Health and Care Excellence (NICE)(19) gives guidance on the key interventional components to reduce the risk or manage overweight and obesity. Principally, these include the adoption of a healthy diet, addressing lifestyle risk factors within the family and social settings, i.e., preschools, reducing sedentary lifestyle, and supporting behavioural change. The UK Government's Tackling Obesity Policy Paper(20) aims to place prevention at the heart of the health agenda to proactively tackle the burden of obesity for adults and children. The Paper suggests that an integrated, whole systems approach is likely to be the most effective in addressing the complex pathways to obesity. It is acknowledged that controlling the problem of childhood obesity is a shared responsibility of the Government, local authorities, and the NHS, in addition to the food industry, schools, and local communities.(20) Preventative and treatment interventions for obesity in early childhood can occur in various settings, typically family homes, nurseries, pre-school, or healthcare settings. Interventions in community settings, such as nurseries or pre-schools, are likely to be preventative and universal (for all children), and have the potential to reach more children.(21,22)

The high prevalence of childhood obesity in the UK supports the need for early interventions. To inform such interventions it is crucial to understand which interventions have been successful. It is also important to identify interventions with longer follow-up periods. The aim of this review was to synthesise evidence of interventions implemented and evaluated in the

For peer review only

UK that aim to prevent or treat obesity in children aged 6 months to five years. We restricted this review to UK interventions as early childhood environments can differ markedly across countries. Patient and public involvement workshops were conducted to help inform our approach and interpret findings.

MATERIALS AND METHODS

Design

This systematic review adhered to the Preferred Reporting Items for Systematic Reviews and Meta-Analyses (PRISMA) statement(23) and presents the UK findings of the PROSPERO-registered protocol CRD42021290676.

Search Strategy

In November 2021, we searched nine databases for obesity interventions among children under five years old published in peer reviewed journals (selected databases and search terms used are shown in Appendix 1). The searches were updated in March 2023. In addition to database searches, the authors searched reference lists of similar reviews. No language restrictions were applied. Our searches included international literature; however, here we report findings from UK studies only. Applicability and transferability of the interventions conducted elsewhere might be limited due to variability in obesity prevalence, resource availability, policy landscape, and delivery of the programmes.(24)

Inclusion and exclusion criteria

Primary research studies were included if they fulfilled the following criteria:

Population: children aged 6 months-5 years.

For peer review only

Intervention: Pre- and post-intervention studies including randomised controlled trials, quasi-experimental studies, (individual, family- or community-based).

Comparison: pre- vs post-intervention and/or intervention vs control group.

Outcomes: at least one anthropometric outcome (e.g., BMI, BMI z-score, BMI percentile, waist circumference, skinfold measurements). Anthropometric measurements were chosen as an outcome because these are the most robust way of assessing impact, compared to other potential outcomes (e.g., diet or physical activity).

The search was limited to studies published during the previous 12 years (2011-2023) to reflect current or recent interventions and programmes, to understand what works in the current context. Previous reviews found no studies conducted in the UK, meaning it is unlikely that there were UK interventions robustly evaluated prior to 2011.(25,26)

Selection of studies and data extraction

Search results were imported into Endnote, where duplicates were removed, before being imported into EPPI Reviewer Web,(27) in which further duplicate searches were undertaken. Two reviewers (SM and MS) independently double screened on title and abstract. Using EPPI-Reviewer software, we applied an ‘active learning approach’, where the prioritisation of records was periodically refreshed during screening so that the most relevant articles were screened first.(28) A graphical output was used to indicate when to stop screening, i.e., when the number of relevant studies had plateaued (Appendix 2). A classifier model was then built using the machine learning algorithm and applied to unscreened items, which generates a score (0-100) indicating relevance; this process reduces the likelihood that relevant studies would be missed. Reviewers independently screened all records with a score higher than 30.

For peer review only

Searches included children aged 0-5 years, interventions that targeted infant feeding in the first 6 months of life were excluded. Evidence suggests that breastfeeding is optimal in terms of growth, development, maturation of the immune system and programming of the metabolic system, and that formula feeding can be associated with rapid weight gain and later risk of obesity.(29) However, the association is complex and there is research that suggests best practice formula feeding can reduce rapid weight gain.(30) For this reason and owing to the complexity of the evidence, we did not include interventions that promote breastfeeding as obesity prevention interventions. The aim of this work was to assess the evidence of interventions promoting health growth in the early years for both breast and formula fed children. Reviewers developed a data extraction tool and data were doubly extracted. The data extracted included information on trial identification (name of study, authors, year of publication), country (country the trial took place and country of research), trial description, trial duration and follow-up, trial characteristics (preventative/treatment, intervention/programme), participants' characteristics (numbers, ages), and results regarding changes on zBMI. The reviewers double screened independently, and any disagreements were resolved through discussion.

Data synthesis

We performed a narrative synthesis of findings, summarising the effect estimates; meta-analysis was not possible owing to heterogeneity in intervention approaches, i.e., components of the interventions, participants targeted, site of delivery, duration, intensity, and length of follow-up. The included studies are presented in two sections: "preventative" or "treatment", based on their inclusion criteria. Universal interventions with children of any weight status are

For peer review only

included in the “preventative” category, while interventions that recruited children with overweight or obesity only are classified as “treatment” interventions. We report the mean difference of any anthropometric outcomes both between-group (i.e., intervention vs. control), and within-group (i.e., before vs. after). Measurements obtained at any time point (e.g., mid-intervention, right after the intervention, after the end of the intervention) were considered for this review. If studies provided the mean value of anthropometric outcomes before and after the intervention or in the control and the intervention group, two reviewers (SM &MS) calculated the mean difference and the standard deviation of the change using formulas that are suggested by the Cochrane Handbook.(31) The alpha level of significance was $p<0.05$.

Risk of bias assessment

Two reviewers (SM and MS) independently assessed the bias using the Cochrane risk of bias assessment tool (RoB 2) to evaluate the included randomised trials.(32) For one study that was an impact assessment, the ROBINS-I tool was used which is suitable for non-randomised studies.(33) Detailed bias assessment can be found in Appendix 3.

Patient and Public Involvement

This approach for this project was developed in collaboration with the Family Research Advisory Group, facilitated by the National’s Children’s Bureau (NCB) (30). An initial patient and public involvement (PPI) session was conducted at the outset of the project with the Family Research Advisory Group, which consisted of a diverse group of six parents. We discussed the current evidence of obesity interventions for young children in the UK and what parents/public contributors perceived to be the elements or components of interventions that made them effective. A second session was conducted at the end of the project, where review findings

For peer review only

were discussed. Parents/public contributors were invited to discuss the challenges of implementing effective interventions, how to effectively evaluate interventions in the early years, and the importance of parental involvement in effective interventions.

RESULTS

Description of the studies

In total, the literature search yielded 34,572 potentially relevant articles. After duplicates removal, 21,547 articles were eligible for screening. Two independent reviewers (SM & MS) manually screened 9,538 records, in title and abstract, of which 6,217 were manually removed (Figure 1). Any differences were resolved by reconciliation and, if necessary, in consultations with the other authors. An additional 12,004 records were removed based on title and abstract, as indicated by the machine learning algorithm. A total of 3,326 records were included for full-text screening, two of which could not be retrieved, as we could not gain access to them through the University of London Library. Out of the 3,324 remaining records, 3,108 were manually excluded (reasons for exclusion presented in Figure 1), leaving 217 relevant studies. Out of the 217 international studies, five studies (six trials) were conducted in the UK and were included in the final review.(34–38)

One study was conducted in Lancashire,(34) one in Glasgow,(35) one in two areas in North and Central England, (36) one in Cornwall,(37) and one in Hertfordshire(38). There were two randomised controlled trials,(38) one cluster randomised trial (CRT),(34) two feasibility CRTs,(35,36) and one before/after impact assessment of a weight management programme.(37) All studies described interventions with both diet and physical activity outcomes. Sample sizes ranged from 42 to 117. Intervention duration ranged from eight weeks

For peer review only

to 12 months. The follow-up measurements were obtained during the intervention for one study(35) and after the intervention for the rest, ranging from 6 to 30 months from the start of the intervention. Two trials targeted children living with overweight, obesity, or at high risk, (37),(38) while the remaining four were preventative interventions,(34–36,38) and targeted children of any weight status (children in the general population, not necessarily living with overweight or obesity). Detailed information for each of the included trials can be found in Table 1.

[Figure 1]

Intervention characteristics and effects

Preventative interventions

The four preventative trials include the *Be active, Eat healthy/Healthy Heroes*(34) intervention, the *ToyBox*(35) intervention, the *Health Exercise Nutrition for the Really Young (HENRY)*(36) programme, and the *Planet Munch* programme, trial 2.(38)

Three out of four trials (Be Active, Eat healthy/Healthy Heroes, ToyBox, HENRY) focused on disadvantaged families or deprived areas.(34–36) Most (34–36)(Be Active, Eat healthy/Healthy Heroes, ToyBox, HENRY) were administered by preschool staff, which had been appropriately trained, while one (Planet Munch)(38) was delivered by a trained multidisciplinary team including dietitians, nutritionists, community artists, and children’s centre staff. Two interventions targeted and made changes in preschools’ policies; for example, by informing the food policy(34) (Be Active, Eat healthy/Healthy Heroes) or by increasing the time allocated to physical activity (ToyBox).(35) Interventions also targeted the home environment, for example by providing healthy cooking sessions(34,38) (Be Active, Eat healthy/Healthy Heroes, Planet

For peer review only

Munch) or sending materials for home-based physical activities. (ToyBox, Planet Munch).(35,38)

One study(36) (HENRY) was delivered to parents through workshops organized in early years centres, with recommendations provided on nutrition, physical activity, and parenting. Planet Munch(38) included workshops for parents and children, that aimed to help them improve their dietary and physical activity practices and adopt a healthier lifestyle.

All studies involved a control group; in the majority of cases, the control group received standard care (Be Active, Eat healthy/Healthy Heroes, ToyBox, HENRY),(34–36) while in one study (Planet Munch),(38) the control group was a delayed intervention group. The risk assessment revealed that *Be active, eat healthy/Healthy Heroes*, *ToyBox, HENRY and Planet Munch* had a high-risk of bias, with the most studies having high or moderate risk of bias in the randomisation process and the intervention adherence (Appendix 3).

Only one trial (Be Active, Eat healthy/Healthy Heroes)(34) found the intervention group to have significantly decreased their zBMI after the intervention, compared to the control group [-0.74, (-1.10 to -0.38)]. Two studies(36,38) also found a lower zBMI in the intervention compared to the control group; however, there was either no statistical comparison between them (HENRY),(36) or the difference was not statistically significant (Planet Munch).(38) In one trial(35) (ToyBox) the zBMI had been raised for both groups; nonetheless, this increase was larger for the control group (Table 1).

When looking at zBMI changes within the intervention group, three studies showed reductions, ranging from -0.02 (-0.2 to 0.2) to -0.9 (95% CIs not given) (Be Active, Eat healthy/Healthy Heroes, HENRY, Planet Munch).(34,36,38) In one study, this reduction was statistically significant (Be Active, Eat healthy/Healthy Heroes),(34) in one no statistical comparison was

For peer review only

reported (HENRY),(36) and one study found the reduction not to be significant (Planet Munch).(38) Finally, one trial (Toy Box)(35) reported zBMI to have increased by 0.02 (-0.11 to 0.15). A second follow-up measurement took place in one of the studies (Planet Munch);(38) while there was a decrease compared to baseline, this was not significant (Table 1).

[Table 1]

Overweight and obesity treatment interventions

We identified two treatment trials. The first was *The Lifestyles, Eating, and Activity for Families (LEAF)*(37) programme, which was an impact assessment of a childhood obesity service and the treatment trial of *Planet Munch*.(38) *The LEAF programme* targeted children with severe obesity or severe obesity with a relevant comorbidity. The programme consisted of an initial home visit, workshops, and follow-up clinic appointments. A community paediatrician, a specialist dietitian, and a physical activity advisor were involved for each child. There was no control group in LEAF. In terms of within-subject changes, the *LEAF* programme found a significant reduction of zBMI, following programme participation (Table 1). The trial was assessed to have a high risk of bias mainly due to missing data.

The treatment trial of *Planet Munch* targeted children with overweight/obesity, or who were high-risk. As with the prevention trial, it was delivered by a multi-disciplinary team through healthy lifestyle workshops. Planet Munch included a control group (delayed intervention group). At the end of the intervention, after accounting for age and gender, the intervention group had lower mean zBMI score than the control group. For Planet Munch, there was a significant reduction in the zBMI of the intervention group, from baseline to the end of the intervention, as well as from baseline to the 2-year follow-up (Table 1)

For peer review only - http://bmjopen.bmj.com/site/about/guidelines.xhtml

Patient and Public Involvement

Parents, acting as public advisors, were mixed in their views, around the potential impact of obesity interventions in early childhood; half (n=3) were positive that interventions in the early years could be effective, while half were sceptical. All parents perceived preventative interventions to be more likely to have impact and the vast majority (n=5) were in favour of multi component interventions incorporating of dietary and physical activity components. Parents reported mixed views in relation to intervention approach; three parents favoured educational interventions around living healthier lifestyles, two favoured motivational approaches, and one favoured changing environments to provide positive effect on obesity. In terms of the interpretation of findings, all parents were surprised at how few interventions and robust evaluations there were in the UK. All parents reported that the cost of living a healthy lifestyle (e.g., buying healthy fresh food) was a crucial factor and that there should be more financial help for parents of young children. Parents also agreed that evaluations of interventions should be better funded, to ensure they are robust; for example, by following participants up over longer time frames. All parent agreed that people living with obesity should be consulted and involved in developing interventions, in addition to families and early years practitioners, in order to improve effectiveness.

DISCUSSION

The aim of this systematic review was to identify and evaluate interventions and programmes designed to prevent or treat obesity among children aged 6 months-5 years, implemented and evaluated in the UK. Six studies met the inclusion criteria, all of which included a combination of diet and physical activity components. Compared to baseline, three interventions reported a

For peer review only

reduction in zBMI immediately after completing the intervention(37,38) and four interventions a while after the intervention end,(34,36,38) with the effect ranging from -0.02 to -0.9 for the preventative interventions and from -0.3 to -0.5 for the treatment ones. Three of these reductions were significant(34,37,38) (two of which targeted children with overweight/obesity).(37,38) Compared to the control conditions, five interventions showed a reduction in zBMI,(34–36,38) of which one was significant.(34)

This is the first systematic review that focuses on childhood obesity interventions among children aged 6 months-5 years evaluated in the UK. Our findings support existing evidence from international reviews that multicomponent interventions, with both dietary behaviours and physical activity elements, are effective in reducing zBMI among pre-schoolers.(26,39,40) Small effects were found at individual level; however, if the interventions are delivered at population level, the effects can be larger and meaningful.(41) The present work contributes to the research by synthesizing the evidence of recent interventions and assessing their effects on children of any weight status or on children with overweight and obesity.

The current review highlights a lack of UK interventions with robust evaluation. Only Planet Munch was evaluated through a randomised controlled trial. ToyBox and HENRY were evaluated with feasibility cluster randomised trials without a follow-on full trial being published, and these feasibility trials were not adequately powered to detect zBMI changes. Methodological limitations across the studies identified, such as unclear adherence to the intervention, limit our ability to draw firm conclusions about their impact. Evaluating interventions is complex; it is important not only to ascertain impact , but also when, how, and in which conditions and for whom interventions work. A theoretical framework, a process to

For peer review only

identify failures in implementation, an assessment of the sample, and a range of outcomes, in addition to adaptation to local settings, are important contributory factors to the effectiveness and applicability of an intervention.(42,43)

Of the studies identified in this review, only two were followed up beyond 12-months(34,38) meaning there is very little evidence for longer-term effects. All the studies included in this review were small-scale evaluations and not easily generalisable. While effect sizes give some measure of clinical importance (if adequately powered), statistical significance is affected by the sample size, and so has limited importance.(44) A reduction in zBMI may be clinically relevant when setting a child on a healthier growth trajectory and improving health outcomes.(45)

Overall, we found a lack of evaluations of interventions that recorded anthropometric outcomes. There were evaluations that reported indirect indicators of intervention impact including reductions in calorie intake or increases in physical activity; however, these indicators can be prone to bias. Children and parents often underreport their energy intake,(46),(47) with greater underreporting often observed among children living with obesity.(48) Subjective measures of physical activity also often have low validity and may be biased due to poor recall or social desirability.(49) There are evaluations of interventions (e.g., the HENRY programme) that show effectiveness in changing energy balance behaviours(50) but have not been included in this review.

Within a whole-systems approach, local authorities can play a leading role in influencing health behaviour. However, it is important to recognize that financial constraints can limit local action to predominantly statutory services. Owing to these and other limitations, many local

For peer review only

authorities have developed interventions relevant to the early years which have not been subject to robust assessment. These are often brief interventions (e.g., cooking classes or baby yoga), for which there is no evidence of meaningful and sustained lifestyle changes. Published studies also had a high risk of bias, limiting the confidence in their results. Additionally, findings were difficult to compare owing to heterogeneity in study designs, intervention approaches, and outcome reporting. Some of the reported reductions in zBMI should be interpreted with caution. For example, children that received the *HENRY* intervention started the programme with higher mean zBMI than those in the control group, which may have influenced the effect size. This highlights the need for appropriately designed RCTs with stated primary outcomes, in order to evaluate obesity interventions. Indicators of eating behaviour, physical activity and other energy balance behaviours are important, anthropometric measures remain the most robust and reliable measure of intervention effectiveness.

Findings from this review suggest that interventions that promote healthy growth in the early years could be effective in reducing the risk of obesity as part of a programme of policies and interventions for young children and families. However, all studies would benefit from evaluation using large-scale RCTs before firm conclusions can be drawn. Unpublished data were provided for two trials relating to the *Planet Munch* (formerly Trim Tots) intervention, which found reductions in zBMI for children living with overweight or obesity or who were at high risk. Importantly, reductions were maintained at longer-term follow-up, up to 24 months after completing the intervention. *Planet Munch* was the only preschool obesity intervention in the UK that complied with all NICE recommendations i.e., provided advice on how to achieve a

For peer review only

healthy diet, encouraged physical activity and included behaviour change strategies in the whole family.

The *LEAF* Programme was also effective in reducing zBMI but was only delivered to children with severe obesity and was not evaluated in an RCT. Our findings are consistent with previous work,(26) which found obesity management interventions to be more effective compared to preventative interventions in young children. This is an expected outcome, considering the different populations in treatment and prevention trials. Treatment interventions aim to slow weight gain and normalise zBMI while preventative interventions aim to maintain a healthy growth trajectory. Both aim to promote healthy lifestyle behaviours.

This systematic review has limitations. The initial search strategy included international evidence without applying limits on geographical location or language and thus yielded a high number of records. We used EPPI-Reviewer software to apply an active learning approach, which greatly reduced screening time but resulted in 12,004 records being excluded without being screened. Though we also conducted a hand search, there may be relevant studies that have not been included in this review. We found but did not include a child-care self-assessment intervention that aimed to improved physical activity, oral health and nutrition for children aged 2-4 years and was delivered in nurseries.(51) The study was published but not formally peer reviewed, meaning it did not fulfil our criteria for inclusion. We also excluded studies published before 2011; however, previous systematic reviews have not included any UK studies conducted prior to that point, so it is unlikely that any robustly evaluated interventions had been conducted.

For peer review only

Only a small number of preschool obesity interventions have been implemented and evaluated in the UK and a smaller number have been robustly evaluated in randomised controlled trials. Meanwhile obesity prevalence and the health inequalities remain high in the UK. Obesity policies and interventions are not always implemented or delivered at scale, and implemented interventions are rarely evaluated robustly. Interventions often target individual behaviour changes without addressing the structural and systemic determinants of obesity.(52)The evaluation outcomes of some interventions are encouraging, but more evidence is needed via larger scale trials. However, scaling-up interventions can be challenging, as adaptations are often required, to meet real-world contexts. Intervention effects can also decrease when applied to the general population, so adaptations should consider the context, implementation and setting of the intervention.(50,53) More research is required to understand what support might be most useful to UK pre-schoolers and their families.

CONCLUSIONS

This review found that there are very few evaluations of childhood obesity interventions in preschool children in the UK. Some interventions reported effect sizes that could be clinically significant if replicated in larger sample sizes. Effect sizes were smaller in preventative interventions where the potential for zBMI change is smaller compared to children living with excess weight at the outset. However, improvements in lifestyle behaviours were reported that may reduce the risk of excess weight gain in childhood. More interventions that aim to prevent and treat obesity in preschool children are needed in the UK. Robust evaluation, ideally in RCTs will be crucial in order to guide policy makers and inform evidence-based practice in families, in communities and local authorities in the UK.

For peer review only

Acknowledgements: We thank Dr. Julie Lanigan and all the Planet Munch team that agreed to share with us findings from their unpublished work, entitled: 'The TrimTots Healthy Lifestyle Programme for prevention of obesity in preschool children: evidence from 2 randomised controlled trials'. Also, we would like to thank Dr. Steven Hope for commenting on the final draft of the manuscript. Finally, we would like to thank the Family Research Advisory Group for their contribution in our research.

Authorship confirmation: SR and RV conceived and designed the project. SM, SR and CS designed the search strategy, and SM, MS and CS conducted the searches. SM, MS and JP conducted screening, data extraction, and assessment of bias. JL provided data from one unpublished study (two trials). SM, MS and SR analysed the data. All authors contributed to the interpretation of findings. SM drafted the paper and all authors revised and edited the manuscript, before approving the final draft.

Data availability statement: All data relevant to the study are included in the article or uploaded as supplementary information.

Ethics statement: This study was exempt from the requirement of approval by the UCL Ethics Committee as it used secondary data available in the public domain and it is not possible to identify individuals from the information provided.

For peer review only - http://bmjopen.bmj.com/site/about/guidelines.xhtml

References

1. NHS Digital [Internet]. [cited 2022 Nov 15]. National Child Measurement Programme, England 2020/21 School Year. Available from: https://digital.nhs.uk/data-and-information/publications/statistical/national-child-measurement-programme/2020-21-school-year

2. NHS Digital [Internet]. [cited 2022 Nov 15]. National Child Measurement Programme, England, Provisional 2021/22 School Year Outputs. Available from: https://digital.nhs.uk/data-and-information/publications/statistical/national-child-measurement-programme/england-provisional-2021-22-school-year-outputs

3. Umer A, Kelley GA, Cottrell LE, Giacobbi P, Innes KE, Lilly CL. Childhood obesity and adult cardiovascular disease risk factors: A systematic review with meta-analysis. BMC Public Health. 2017;17(1):1–24.

4. Rankin J, Matthews L, Cobley S, Han A, Sanders R, Wiltshire HD, et al. Psychological consequences of childhood obesity: psychiatric comorbidity and prevention. Adolesc Health Med Ther. 2016;Volume 7:125–46.

5. Van Geel M, Vedder P, Tanilon J. Are overweight and obese youths more often bullied by their peers? A meta-analysis on the relation between weight status and bullying. Int J Obes. 2014;38(10):1263–7.

6. Abdelaal M, le Roux CW, Docherty NG. Morbidity and mortality associated with obesity. Ann Transl Med. 2017;5(7):1–12.

7. Simmonds M, Llewellyn A, Owen CG, Woolacott N. Predicting adult obesity from childhood obesity: A systematic review and meta-analysis. Obes Rev. 2016;17(2):95–107.

8. Mandy Geserick, M.Sc., Mandy Vogel, Ph.D., Ruth Gausche, M.B.A., Tobias Lipek, M.D., Ulrike Spielau, M.Sc., Eberhard Keller, M.D., Roland Pfäffle, M.D., Wieland Kiess, M.D., and Antje Körner MD, M V, R G, T L, U S, E K, et al. Acceleration of BMI in early childhood and risk of sustained obesity. N Engl J Med. 2018;

9. Pearce M, Webb-Phillips S, Bray I. Changes in objectively measured BMI in children aged 4-11 years: Data from the National Child Measurement Programme. J Public Health U K. 2016;38(3):459–66.

10. Anderson EL, Tilling K, Fraser A, Macdonald-Wallis C, Emmett P, Cribb V, et al. Estimating trajectories of energy intake through childhood and adolescence using linear-spline multilevel models. Epidemiology. 2013;24(4):507–15.

11. Hebestreit A, Börnhorst C, Pala V, Barba G, Eiben G, Veidebaum T, et al. Dietary energy density in young children across Europe. Int J Obes. 2014;38:S124–34.

For peer review only - http://bmjopen.bmj.com/site/about/guidelines.xhtml

12. Niederer I, Zahner L, Bürgi F, Kriemler S, Ebenegger V, Marques-Vidal P, et al. BMI group-related differences in physical fitness and physical activity in preschool-Age children: A cross-sectional analysis. Res Q Exerc Sport. 2012;83(1):12–9.

13. Public Health England. Government Dietary Recommendations. Gov Diet Recomm. 2016;1–12.

14. NHS Digital. Health Survey for England, Fruit and Vegetable Consumption, 2018. 2019.

15. O'Dwyer M V., Foweather L, Stratton G, Ridgers ND. Physical activity in non-overweight and overweight UK preschool children: Preliminary findings and methods of the Active Play Project. Sci Sports. 2011;26(6):345–9.

16. NHS England. Physical activity guidelines for children (under 5 years).

17. Ambrosini GL, Emmett PM, Northstone K, Howe LD, Tilling K, Jebb SA. Identification of a dietary pattern prospectively associated with increased adiposity during childhood and adolescence. World Rev Nutr Diet. 2014;109(2):13.

18. Jones RA, Hinkley T, Okely AD, Salmon J. Tracking physical activity and sedentary behavior in childhood: A systematic review. Am J Prev Med. 2013;44(6):651–8.

19. National Institute for Health and Care Excellence. Obesity in children and young people : prevention and lifestyle weight management programmes. (July 2015).

20. Department of Health and Social Care. Tackling obesity: empowering adults and children to live healthier lives. 2020.

21. Smith JD, Fu E, Kobayashi M. Prevention and Management of Childhood Obesity and its Psychological and Health Comorbidities. Annu Rev Clin Psychol [Internet]. 2020 May 7 [cited 2023 Dec 1];16:351–78. Available from: https://www.ncbi.nlm.nih.gov/pmc/articles/PMC7259820/

22. Ickes MJ, McMullen J, Haider T, Sharma M. Global School-Based Childhood Obesity Interventions: A Review. Int J Environ Res Public Health [Internet]. 2014 Sep [cited 2023 Dec 1];11(9):8940–61. Available from: https://www.mdpi.com/1660-4601/11/9/8940

23. Page MJ, McKenzie JE, Bossuyt PM, Boutron I, Hoffmann TC, Mulrow CD, et al. The PRISMA 2020 statement: an updated guideline for reporting systematic reviews. BMJ [Internet]. 2021 Mar 29 [cited 2023 Jun 8];372:n71. Available from: https://www.bmj.com/content/372/bmj.n71

24. Wang S, Moss JR, Hiller JE. Applicability and transferability of interventions in evidence-based public health. Health Promot Int. 2006;21(1):76–83.

25. Flynn MAT, McNeil DA, Maloff B, Mutasingwa D, Wu M, Ford C, et al. Reducing obesity and related chronic disease risk in children and youth: A synthesis of evidence with 'best practice' recommendations. Vol. 7, Obesity Reviews. 2006. 7–66 p.

For peer review only - http://bmjopen.bmj.com/site/about/guidelines.xhtml

26. Ling J, Robbins LB, Wen F. Interventions to prevent and manage overweight or obesity in preschool children: A systematic review. Int J Nurs Stud. 2016;53:270–89.

27. Institute of Education (IOE). EPPI-Reviewer Web (Beta) [Internet]. Available from: https://eppi.ioe.ac.uk/EPPIReviewer-Web/home

28. EPPI Reviewer. Priority Screening.

29. Kouwenhoven SMP, Muts J, Finken MJJ, van Goudoever JB. Low-Protein Infant Formula and Obesity Risk. Nutrients [Internet]. 2022 Jun 30 [cited 2023 Nov 30];14(13):2728. Available from: https://www.ncbi.nlm.nih.gov/pmc/articles/PMC9268498/

30. Appleton J, Russell CG, Laws R, Fowler C, Campbell K, Denney-Wilson E. Infant formula feeding practices associated with rapid weight gain: A systematic review. Matern Child Nutr [Internet]. 2018 Apr 14 [cited 2023 Nov 30];14(3):e12602. Available from: https://www.ncbi.nlm.nih.gov/pmc/articles/PMC6866175/

31. Julian, Higgins, James T. 6.3. 2022. Cochrane Handbook for Systematic Reviews of Interventions. Available from: https://training.cochrane.org/handbook/current

32. Sterne JAC, Savović J, Page MJ, Elbers RG, Blencowe NS, Boutron I, et al. RoB 2: A revised tool for assessing risk of bias in randomised trials. The BMJ. 2019;366:1–8.

33. Sterne JA, Hernán MA, Reeves BC, Savović J, Berkman ND, Viswanathan M, et al. ROBINS-I: A tool for assessing risk of bias in non-randomised studies of interventions. BMJ Online. 2016;355:4–10.

34. Hodgkinson A, Abbott J, Hurley MA, Lowe N, Qualter P. An educational intervention to prevent overweight in pre-school years: a cluster randomised trial with a focus on disadvantaged families. BMC Public Health [Internet]. 2019 Nov 1 [cited 2022 Nov 24];19(1):1430. Available from: https://doi.org/10.1186/s12889-019-7595-2

35. Malden S, Reilly JJ, Gibson AM, Bardid F, Summerbell C, De Craemer M, et al. A feasibility cluster randomised controlled trial of a preschool obesity prevention intervention: ToyBox-Scotland. Pilot Feasibility Stud. 2019;5(1):1–11.

36. Bryant M, Collinson M, Burton W, Stamp E, Schofield H, Copsey B, et al. Cluster randomised controlled feasibility study of HENRY: a community-based intervention aimed at reducing obesity rates in preschool children. Pilot Feasibility Stud. 2021;7(1):59.

37. Tee LP, Brandreth RA, Sauven N, Clarke L, Frampton I. Successful outcomes in childrens specialist weight management: Impact assessment of a novel early years weight management programme. J Hum Nutr Diet. 2021;34(5):819–26.

38. Julie Lanigan, Sarah Collins, Kerry Coyte, Toni Birbara, Maria Kokoreli, Tim Cole, et al. The TrimTots Healthy Lifestyle Programme for prevention of obesity in preschool children: evidence from 2 randomised controlled trials. Unpublished.

For peer review only - http://bmjopen.bmj.com/site/about/guidelines.xhtml

39. Brown T, Moore TH, Hooper L, Gao Y, Zayegh A, Ijaz S, et al. Interventions for preventing obesity in children. Cochrane Database Syst Rev. 2019;2019(7).

40. Colquitt JL, Loveman E, O'Malley C, Azevedo LB, Mead E, Al-Khudairy L, Ells LJ, Metzendorf MI RK. Diet, physical activity, and behavioural interventions for the treatment of overweight or obesity in preschool children up to the age of 6 years (Review). Cochrane Database Syst Rev. 2016;

41. Matthay EC, Hagan E, Gottlieb LM, Tan ML, Vlahov D, Adler N, et al. Powering population health research: Considerations for plausible and actionable effect sizes. SSM - Popul Health. 2021;14:100789.

42. Mills A, Gilson L, Hanson K, Palmer N, Lagarde M. What do we mean by rigorous health-systems research? The Lancet. 2008;372(9649):1527–9.

43. Craig P, Dieppe P, Macintyre S, Mitchie S, Nazareth I, Petticrew M. Developing and evaluating complex interventions: The new Medical Research Council guidance. Bmj. 2008;337(7676):979–83.

44. Dunkler D, Haller M, Oberbauer R, Heinze G. To test or to estimate? P-values versus effect sizes. Transpl Int. 2020;33(1):50–5.

45. Ryan DH, Yockey SR. Weight Loss and Improvement in Comorbidity: Differences at 5%, 10%, 15%, and Over. Curr Obes Rep. 2017;6(2):187–94.

46. Livingstone MBE, Robson PJ, Wallace JMW. Issues in dietary intake assessment of children and adolescents. Br J Nutr. 2004;92(S2):S213–22.

47. Foster E, Hawkins A, Barton KL, Stamp E, Matthews JNS, Adamson AJ. Development of food photographs for use with children aged 18 months to 16 years: Comparison against weighed food diaries-The Young Person's Food Atlas (UK). PLoS ONE. 2017;12(2):1–18.

48. Swinburn B, Sacks G, Ravussin E. Increased food energy supply is more than sufficient to explain the US epidemic of obesity. Am J Clin Nutr. 2009;90(6):1453–6.

49. Strath SJ, Kaminsky LA, Ainsworth BE, Ekelund U, Freedson PS, Gary RA, et al. Guide to the assessment of physical activity: Clinical and research applications: A scientific statement from the American Heart association. Circulation. 2013;128(20):2259–79.

50. Pfadenhauer LM, Gerhardus A, Mozygemba K, Lysdahl KB, Booth A, Hofmann B, et al. Making sense of complexity in context and implementation: The Context and Implementation of Complex Interventions (CICI) framework. Implement Sci. 2017;12(1):1–17.

51. Kipping R, Langford R, Brockman R, Wells S, Metcalfe C, Papadaki A, et al. Child-care self-assessment to improve physical activity, oral health and nutrition for 2- to 4-year-olds: a feasibility cluster RCT. Public Health Res [Internet]. 2019 Jul [cited 2023 Nov 30];7(13):1–164. Available from: https://www.journalslibrary.nihr.ac.uk/phr/phr07130

For peer review only - http://bmjopen.bmj.com/site/about/guidelines.xhtml

52. THEIS DRZ, WHITE M. Is Obesity Policy in England Fit for Purpose? Analysis of Government Strategies and Policies, 1992–2020. Milbank Q [Internet]. 2021 Mar [cited 2023 Nov 27];99(1):126–70. Available from: https://www.ncbi.nlm.nih.gov/pmc/articles/PMC7984668/

53. McCrabb S, Lane C, Hall A, Milat A, Bauman A, Sutherland R, et al. Scaling-up evidence-based obesity interventions: A systematic review assessing intervention adaptations and effectiveness and quantifying the scale-up penalty. Obes Rev. 2019;(December 2018):964–82.

For peer review only

Tables included in the text

Table 1 Characteristics of the included studies

Author, year, study type	Participants eligible / targeted	Participants randomized (intervention, control)[a]	Intervention duration	Time of the follow-up measurements	Participants with valid data in the measurements	Change in mean zBMI 1st or 2nd measurement vs baseline (SD / CI 95%)	Change in mean zBMI vs control (SD / CI 95%)	When evaluated
Hodgkinson et al., (2019), Pragmatic CRT	2-4 years, Mean age=NR	87 (48, 39)	6 months	2-years post baseline	81 (47, 34)	-0.18* (SD 0.99)	-0.74* (-1.10 to -0.38)	NR (Trial published in 2019)
Malden et al., (2019), Feasibility CRT	3-5 years, Mean age=4.4	42 (26, 16)	18 weeks	15th-17th week of the intervention	36 (22, 14)	+0.02 (-0.11 to 0.15)	-0.04 (SD 0.13)	2018
Bryant et al., (2021), Feasibility CRT	6 months - 5 years Mean age=NR	117 (47, 70)	8 weeks	11-months post baseline	99 (39, 60)	-0.9 (SD 0.46)	-1.4 (SD 0.08)	NR (Trial published in 2021)
Lanigan et al., (Unpublished) RCT – trial 1	1-5 years, with OW/OB/high risk Mean age=2.4	88 (49, 39)	6 months	6-months post baseline	64 (36, 28)	-0.2 (-0.3 to -0.03)	-0.2 (-0.5 to 0.05)	(Trial not yet published)
				24 months after the end of the intervention	42	-0.3 * (-0.5, -0.1),	NR	(Trial not yet published)
Lanigan et al., (Unpublished) RCT -trial 2	1-5 years, of any weight status. Mean age=2.1	85 (38, 47)	6 months	6-months post baseline	64 (27, 37)	-0.02 (-0.2 to 0.2),	-0.09 (-0.4 to 0.2)	(Trial not yet published)
				12 months after the end of the intervention	39	-0.3 (-0.7, 0.1),	NR	(Trial not yet published)

For peer review only - http://bmjopen.bmj.com/site/about/guidelines.xhtml

Tee et al., (2021), Impact Assessment	Children aged ≤6 years with severe OB Mean age=4.1	147	12 months	12 months post baseline	41	-0.5*	NR	2012-17

CRT, cluster randomised trial, NR, not reported, NA, not applicable, RCT, randomised controlled trial, OW, overweight, OB, obesity
[a]: Numbers at randomisation, *: significant change in zBMI

For peer review only

For peer review only - http://bmjopen.bmj.com/site/about/guidelines.xhtml

Figure legends

Figure 1 Flowchart of the included studies

For peer review only

For peer review only - http://bmjopen.bmj.com/site/about/guidelines.xhtml

For peer review only

For peer review only - http://bmjopen.bmj.com/site/about/guidelines.xhtml

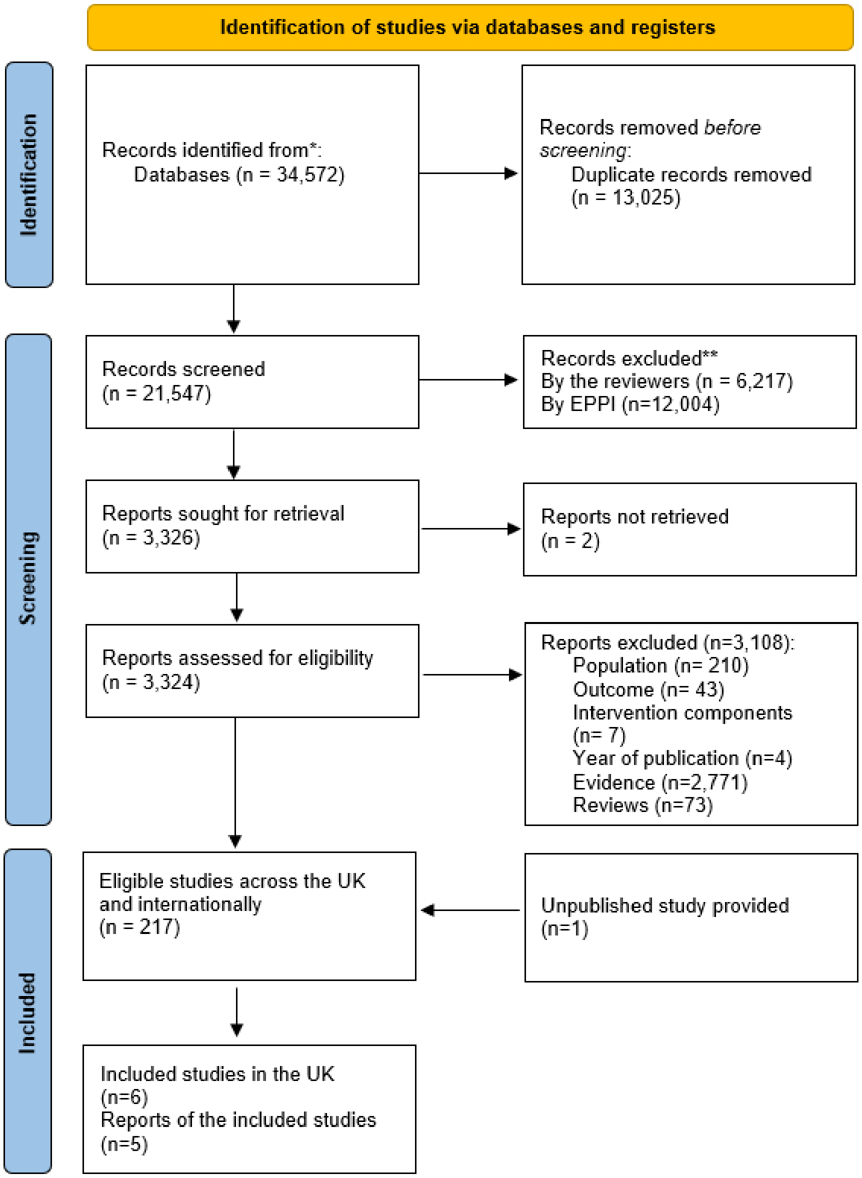

Figure 1 Flowchart of the included studies

408x528mm (38 x 38 DPI)

Supplementary Material

Appendix 1 – Search terms and search strategy

The following databases were searched in November 2021. The searches were updated in March 2023.

1. Pubmed/Medline
2. Cochrane Library Web
3. CINAHL
4. Scopus
5. PsycINFO
6. Web of Science
7. Social Policy and Practice
8. Health Management Information Consortium (HMIC)
9. Trials Register of Promoting Health Interventions (TRoPHI)

The indicative search terms that were used are presented on the table below:

Table S1: Search terms

Concept 1<br><br>Participants	Concept 2<br><br>Exposure	Concept 3<br><br>Outcomes
Subject headings:<br><br>"Child, preschool" OR<br><br>"Schools, nursery" OR<br><br>"Child day care centers"	(("diet" OR "food" OR "beverages" OR "food quality" OR "food preferences" OR "feeding behavior*")<br><br>OR<br><br>("exercise" OR "sedentary behavior" " OR "physical fitness"))<br><br>AND<br><br>("weight reduction programs" OR "health education" OR "health promotion" OR "primary prevention" OR "pilot projects" OR "feasibility studies" OR "program evaluation")	("obesity" OR "body mass index" OR "body weight" OR "body size" OR "body composition" OR "body weight changes" OR

For peer review only

Keywords<br><br>"child preschool" OR "preschool*" OR "preschool*" OR "pre school*" OR "kindergarten" OR "kindergarden" OR "prekinder*" OR "pre kinder*" OR "pre kinder*" OR "headstart" OR "head start" OR "sure start" OR "surestart" OR "family child care home*" OR "family childcare home*" OR "infant*" OR "toddler*" OR "childcare" OR "child-care" OR "child-care" OR "early learning center*" OR "early learning centre*" OR "early child*" OR "early care and education" OR ("child*" AND ("day-care" OR "day-care" OR "daycare")) OR "playgroup*" OR "play group*" OR "schools nursery" OR (("center" OR "center s" OR "centers"OR "centre" OR "centre s" OR "centres") AND "child, preschool") OR "child day care centers" OR "child day care centers")	(("diet" OR "dietary" OR "food" OR "beverages"OR "snack*" OR "fruit*" OR "vegetable*" OR OR "food quality" OR "drink*" OR "juice*" OR "nutrition*" OR "food habit*" OR "food choice*" OR "overeat*" OR "over eat*" OR "weight control" OR "weight management" OR "menu planning" OR "menu choice*" OR "meal choice*" OR "meal*" OR "meal planning" OR ("food" AND "consum*") OR "food preferences" OR "feeding behavior*" OR "feeding behaviour*" OR "energy density" OR "lifestyle behaviour" OR "eating behaviour" OR "healthy eating" OR "health promotion" OR "eating habit*")<br><br>OR<br><br>("exercise" OR "sedentary behaviour OR "physical fitness" OR "movement" OR "playtime"OR "play*" OR "sport*"))<br><br>AND<br><br>("intervention*" OR "program*" OR "programme*" OR "project*" OR "therapeutics" OR "scheme" OR "initiative*" OR "strateg*" OR "service*" OR "primary prevention" OR "health education" OR "health promotion" OR "primary prevention" OR "treat*" OR "therapy*" OR "pilot projects" OR "feasibility studies" OR "program evaluation")	"overweight")("obesity" OR "obes*" OR "body mass index" OR "BMI" OR "bmi z score" OR "bmi z score" OR "body mass index z-score" OR "body mass index z-score" OR "bmi percentile*" OR "bmi centile*" OR "body mass index percentile*" OR "body mass index centile*" OR "adipos*" OR "overweight" OR "healthy weight" OR "weight loss" OR "weight gain")

For peer review only

Search strategy in all databases:

PubMed

#1 Child/setting terms	("child, preschool"[MeSH Terms] OR "child preschool"[Title/Abstract] OR "preschool*"[Title/Abstract] OR "preschool*"[Title/Abstract] OR "pre school*"[Title/Abstract] OR "kindergarten"[Title/Abstract] OR "kindergarden"[Title/Abstract] OR "prekinder*"[Title/Abstract] OR "pre kinder*"[Title/Abstract] OR "pre kinder*"[Title/Abstract] OR "headstart"[Title/Abstract] OR "head start"[Title/Abstract] OR "sure start"[Title/Abstract] OR "surestart"[Title/Abstract] OR "family child care home*"[Title/Abstract] OR "family childcare home*"[Title/Abstract] OR "infant*"[Title/Abstract] OR "toddler*"[Title/Abstract] OR "childcare"[Title/Abstract] OR "child-care"[Title/Abstract] OR "child-care"[Title/Abstract] OR "early learning center*"[Title/Abstract] OR "early learning centre*"[Title/Abstract] OR "early child*"[Title/Abstract] OR "early care and education"[Title/Abstract] OR ("child*"[Title/Abstract] AND ("day-care"[Title/Abstract] OR "day-care"[Title/Abstract] OR "daycare"[Title/Abstract])) OR "playgroup*"[Title/Abstract] OR "play group*"[Title/Abstract] OR "schools, nursery"[MeSH Terms] OR "schools nursery"[Title/Abstract] OR (("center"[Title/Abstract] OR "center s"[Title/Abstract] OR "centers"[Title/Abstract] OR "centre"[Title/Abstract] OR "centre s"[Title/Abstract] OR "centres"[Title/Abstract]) AND "child, preschool"[MeSH Terms]) OR "child day care centers"[MeSH Terms] OR "child day care centers"[Title/Abstract])
#2 Diet terms	("diet"[MeSH Terms] OR "diet"[Title/Abstract] OR "dietary"[Title/Abstract] OR "food"[MeSH Terms] OR "food"[Title/Abstract] OR "beverages"[MeSH Terms] OR "beverages"[Title/Abstract] OR "snack*"[Title/Abstract] OR "fruit*"[Title/Abstract] OR "vegetable*"[Title/Abstract] OR "food quality"[MeSH Terms] OR "food quality"[Title/Abstract] OR "drink*"[Title/Abstract] OR "juice*"[Title/Abstract] OR "nutrition*"[Title/Abstract] OR "food habit*"[Title/Abstract] OR "food choice*"[Title/Abstract] OR "overeat*"[Title/Abstract] OR "over eat*"[Title/Abstract] OR "weight control"[Title/Abstract] OR "weight management"[Title/Abstract] OR "menu planning"[Title/Abstract] OR "menu choice*"[Title/Abstract] OR "meal choice*"[Title/Abstract] OR "meal*"[Title/Abstract] OR "meal planning"[Title/Abstract] OR ("food"[Title/Abstract] AND "consum*"[Title/Abstract]) OR "food preferences"[MeSH Terms] OR "food preferences"[Title/Abstract] OR "feeding behavior*"[MeSH Terms] OR "feeding behavior*"[Title/Abstract] OR "feeding behaviour*"[Title/Abstract] OR "energy density"[Title/Abstract] OR "lifestyle behaviour"[Title/Abstract] OR "eating behaviour"[Title/Abstract] OR "healthy eating"[Title/Abstract] OR "health promotion"[Title/Abstract] OR "eating habit*"[Title/Abstract])
#3 **PA terms**	("exercise"[MeSH Terms] OR "exercise"[Title/Abstract] OR "sedentary behavior"[MeSH Terms] OR "sedentary behavior"[Title/Abstract] OR "sedentary behaviour"[Title/Abstract] OR "physical fitness"[MeSH Terms] OR "physical fitness"[Title/Abstract] OR "movement"[Title/Abstract] OR "playtime"[Title/Abstract] OR "play*"[Title/Abstract] OR "sport*"[Title/Abstract])
#4 Intervention terms	("intervention*"[Title/Abstract] OR "weight reduction programs"[MeSH Terms] OR "program*"[Title/Abstract] OR "programme*"[Title/Abstract] OR "project*"[Title/Abstract] OR "therapeutics"[Title/Abstract] OR "scheme"[Title/Abstract] OR "initiatives"[Title/Abstract] OR "strateg*"[Title/Abstract] OR "service*"[Title/Abstract] OR "pilot projects"[MeSH Terms] OR "pilot projects"[Title/Abstract] OR "feasibility studies"[MeSH Terms] OR "feasibility studies"[Title/Abstract] OR "program evaluation"[MeSH Terms] OR "program evaluation"[Title/Abstract] OR "health education"[MeSH Terms] OR "health education"[Title/Abstract] OR "health promotion"[MeSH Terms] OR "health promotion"[Title/Abstract] OR "primary prevention"[Title/Abstract] OR "treat*"[Title/Abstract] OR "random*"[Title/Abstract] OR "cluster random*"[Title/Abstract] OR "trial*"[Title/Abstract] OR "evaluat*"[Title/Abstract] OR "effective*"[Title/Abstract] OR "mixed methods"[Title/Abstract] OR "impact*"[Title/Abstract])
#5	("intervention*"[Title/Abstract] OR "weight reduction programs"[MeSH Terms] OR "program*"[Title/Abstract] OR "programme*"[Title/Abstract] OR "project*"[Title/Abstract] OR "therapeutics"[Title/Abstract] OR "scheme"[Title/Abstract] OR "initiative*"[Title/Abstract] OR "strateg*"[Title/Abstract] OR "service*"[Title/Abstract] OR "health education"[MeSH Terms] OR "health promotion"[MeSH Terms] OR "primary prevention"[MeSH Terms] OR "primary prevention"[Title/Abstract] OR "health education"[Title/Abstract] OR "health

For peer review only

	promotion"[Title/Abstract] OR "primary prevention"[Title/Abstract] OR "treat*"[Title/Abstract] OR "therapy*"[Title/Abstract] OR "pilot projects"[MeSH Terms] OR "pilot projects"[Title/Abstract] OR "feasibility studies"[MeSH Terms] OR "feasibility studies"[Title/Abstract] OR "program evaluation"[MeSH Terms] OR "program evaluation"[Title/Abstract])
#6	("random*"[Title/Abstract] OR "cluster random*"[Title/Abstract] OR "trial*"[Title/Abstract] OR "evaluat*"[Title/Abstract] OR "effective*"[Title/Abstract] OR "mixed methods"[Title/Abstract] OR "impact*"[Title/Abstract] OR "non randomized controlled trial*"[Title/Abstract] OR "Pragmatic Clinical Trial"[Title/Abstract] OR "controlled clinical trial*"[Title/Abstract] OR "randomized controlled trial*"[Title/Abstract] OR "randomized"[Title/Abstract] OR "randomised"[Title/Abstract] OR "clinical trial*"[MeSH Terms] OR "clinical trial*"[Title/Abstract] OR "randomly"[Title/Abstract] OR "controlled study"[Title/Abstract] OR "controlled trial*"[Title/Abstract] OR "control group"[Title/Abstract] OR "intervention group"[Title/Abstract] OR "experimental group"[Title/Abstract] OR "comparison group"[Title/Abstract] OR "control school"[Title/Abstract] OR "intervention school"[Title/Abstract] OR "control community"[Title/Abstract] OR "intervention community"[Title/Abstract] OR "intervention condition"[Title/Abstract] OR "control condition"[Title/Abstract] OR "treatment group"[Title/Abstract] OR "control participant"[Title/Abstract] OR "quasi experimental design"[Title/Abstract] OR "experimental condition"[Title/Abstract] OR "treatment condition"[Title/Abstract] OR "pilot projects"[MeSH Terms] OR "feasibility studies"[MeSH Terms] OR "program evaluation"[MeSH Terms] OR "pre test"[Title/Abstract] OR "post test"[Title/Abstract] OR "quasi experiment*"[Title/Abstract] OR "blinded"[Title/Abstract] OR "double bind"[Title/Abstract] OR "fidelity"[Title/Abstract] OR "reach"[Title/Abstract] OR "uptake"[Title/Abstract] OR "implementation"[Title/Abstract] OR "intervention effect*"[Title/Abstract] OR "program effect*"[Title/Abstract] OR "programme effect*"[Title/Abstract] OR "proof of concept*"[Title/Abstract] OR "outcome study"[Title/Abstract])
#7	Search: #5 AND #6
#8	Search: #2 OR #3
#9	Search: #8 AND (#4 OR #7)
**#10**	("obesity"[MeSH Terms] OR "obesity"[Title/Abstract] OR "obes*"[Title/Abstract] OR "body mass index"[MeSH Terms] OR "body mass index"[Title/Abstract] OR "BMI"[Title/Abstract] OR "bmi z score"[Title/Abstract] OR "bmi z score"[Title/Abstract] OR "body mass index z-score"[Title/Abstract] OR "body mass index z-score"[Title/Abstract] OR "bmi percentile*"[Title/Abstract] OR "bmi centile*"[Title/Abstract] OR "body mass index percentile*"[Title/Abstract] OR "body mass index centile*"[Title/Abstract] OR "body weight"[MeSH Terms] OR "body size"[MeSH Terms] OR "adipos*"[Title/Abstract] OR "body composition"[MeSH Terms] OR "body weight changes"[MeSH Terms] OR "overweight"[MeSH Terms] OR "overweight"[Title/Abstract] OR "healthy weight"[Title/Abstract] OR "weight loss"[Title/Abstract] OR "weight gain"[Title/Abstract])
**#11**	Search: #1 AND #9 AND #10

For peer review only

CINAHL

#1<br>Population<br>AB	AB (preschool* OR pre-school* OR “pre-school*” OR kindergar?en OR prekinder* OR pre-Kinder* OR “pre Kinder*” OR headstart OR “head start” OR “surestart” OR “sure start” OR “family child care home” OR “family childcare home” OR “infant*” OR “toddler*” OR “child care” OR “child-care” OR childcare OR “early learning centre” OR “early child*” OR “early care and education” OR (child* AND “day care” OR “daycare” OR “day-care” OR playgroup* OR “nurser*” OR child day care centre*)) **OR** TI ((preschool* OR pre-school* OR “pre-school*” OR kindergar?en OR prekinder* OR pre-Kinder* OR “pre Kinder*” OR headstart OR “head start” OR “surestart” OR “sure start” OR “family child care home” OR “family childcare home” OR “infant*” OR “toddler*” OR “child care” OR “child-care” OR childcare OR “early learning centre” OR “early child*” OR “early care and education” OR (child* AND “day care” OR “daycare” OR “day-care” OR playgroup* OR “nurser*” OR child day care centre*))
#2<br>Diet<br>AB	AB (diet* OR dietary OR food* OR snack* OR fruit* OR drink* OR beverage* OR juice* OR vegetable* OR meal* OR nutrition* OR intake* OR consum* OR “food quality” OR “food preference*” OR “energy density” OR “lifestyle behavio#r” OR “eating behavio#r” OR “healthy eating” OR “feeding behavio#r” OR “health promotion” OR “eating habit*” OR “food habit*” OR “food choice*” OR “overeat*” OR “over eat*” OR “weight control” OR “weight management” OR “menu planning” OR “menu choice” OR “meal choice*” OR “meal*” OR “meal planning” OR (food* AND consum*) OR “eating habit*”) **OR** TI (diet* OR dietary OR food* OR snack* OR fruit* OR drink* OR beverage* OR juice* OR vegetable* OR meal* OR nutrition* OR intake* OR consum* OR “food quality” OR “food preference*” OR “energy density” OR “lifestyle behavio#r” OR “eating behavio#r” OR “healthy eating” OR “feeding behavio#r” OR “health promotion” OR “eating habit*” OR “food habit*” OR “food choice*” OR “overeat*” OR “over eat*” OR “weight control” OR “weight management” OR “menu planning” OR “menu choice” OR “meal choice*” OR “meal*” OR “meal planning” OR (food* AND consum*) OR “eating habit*”)
#3<br>PA<br>AB	AB (“physical* activ*” OR playtime OR sedentary OR exercise* OR play* OR “physical fitness” OR “movement” OR “playtime” OR “sport*”) **OR** TI (“physical* activ*” OR playtime OR sedentary OR exercise* OR play* OR “physical fitness” OR “movement” OR “playtime” OR “sport*”)
#4<br>Interventions<br>AB	AB (“intervention*” OR “weight reduction program*” "program*" OR "programme*" OR "project*" OR "therapeutics" OR "scheme" OR "initiative*" OR "strateg*" OR "life style" OR "service*" OR “pilot project*” OR “feasibility stud*” OR “program evaluation” OR “health education*” OR “health promotion” OR “primary prevention” OR “treat*” OR “random*” OR “cluster random*” OR “trial*” OR “evaluat*” OR “effective*” OR “mixed methods” OR “impact*”) **OR** TI (“intervention*” OR “weight reduction program*” "program*" OR "programme*" OR "project*" OR "therapeutics" OR "scheme" OR "initiative*" OR "strateg*" OR "life style" OR "service*" OR “pilot project*” OR “feasibility stud*” OR “program evaluation” OR “health education*” OR “health promotion” OR “primary prevention” OR “treat*” OR “random*” OR “cluster random*” OR “trial*” OR “evaluat*” OR “effective*” OR “mixed methods” OR “impact*”)
#5<br>Intervention terms	AB ("intervention*" OR "weight reduction programs" OR "program*" OR "programme*" OR "project*" OR "therapeutics" OR "scheme" OR "initiative*" OR "strateg*" OR "service*" OR "health education" OR "health promotion" OR "primary prevention" OR "health education" OR "health promotion" OR "primary prevention" or "treat*" OR therapy* or "pilot projects" OR "feasibility studies" OR "program evaluation") **OR** TI ("intervention*" OR "weight reduction programs" OR "program*" OR "programme*" OR "project*" OR "therapeutics" OR "scheme" OR "initiative*" OR "strateg*" OR "service*" OR "health education" OR "health promotion" OR "primary prevention" OR "health education" OR "health promotion" OR "primary prevention" or "treat*" OR therapy* or "pilot projects" OR "feasibility studies" OR "program evaluation")
#6<br>Evaluati	AB ("random*" OR "cluster random*" OR "trial*" OR "evaluat*" OR "effective*" OR "mixed methods" OR "impact*" OR "Non-Randomized Controlled Trial*" OR "Pragmatic Clinical Trial" OR "controlled Clinical Trial*" OR "randomized controlled trial*" OR “randomized” OR “randomised” OR "clinical trial*" OR “randomly” OR "controlled study" OR "controlled trial*" OR "control group" OR "intervention group" OR "experimental group" OR "comparison group" OR "control school" OR "intervention school" OR "control community" OR "intervention community" OR "intervention condition" OR "control condition" OR "treatment group" OR "control participant" OR "quasi experimental design" OR

For peer review only

on terms	"experimental condition" OR "treatment condition" OR "pilot projects" OR "feasibility studies" OR "program evaluation" OR "pre test" OR "post test" OR "quasi experiment*" OR blinded OR "double bind" OR fidelity OR reach OR uptake OR implementation OR "intervention effect*" OR "program effect*" OR "programme effect*" OR "proof of concept*" OR "outcome study") **OR** TI ("random*" OR "cluster random*" OR "trial*" OR "evaluat*" OR "effective*" OR "mixed methods" OR "impact*" OR "Non-Randomized Controlled Trial*" OR "Pragmatic Clinical Trial" OR "controlled Clinical Trial*" OR "randomized controlled trial*" OR “randomized” OR “randomised” OR "clinical trial*" OR “randomly” OR "controlled study" OR "controlled trial*" OR "control group" OR "intervention group" OR "experimental group" OR "comparison group" OR "control school" OR "intervention school" OR "control community" OR "intervention community" OR "intervention condition" OR "control condition" OR "treatment group" OR "control participant" OR "quasi experimental design" OR "experimental condition" OR "treatment condition" OR "pilot projects" OR "feasibility studies" OR "program evaluation" OR "pre test" OR "post test" OR "quasi experiment*" OR blinded OR "double bind" OR fidelity OR reach OR uptake OR implementation OR "intervention effect*" OR "program effect*" OR "programme effect*" OR "proof of concept*" OR "outcome study")
#7	S5 AND S6
#8	S2 OR S3
#9	S4 OR S7
#10	S8 AND S9<br><br>**Limiters** - Age Groups: Infant, 1-23 months, Child, Preschool 2-5 years
#11	AB ("obesity” OR “obes*” OR "body mass index" OR "BMI" OR "bmi z score" OR "bmi z score" OR "body mass index z-score" OR "body mass index z-score" OR "bmi percentile*" OR "bmi centile*" OR "body mass index percentile*" OR "body mass index centile*" OR "body weight" OR "body size" OR "weight*" OR "waist" OR "adipos*" OR "body composition" OR "overweight" OR “body weight changes” OR “healthy weight” OR “weight loss” OR “weight gain”) **OR** TI ("obesity” OR “obes*” OR "body mass index" OR "BMI" OR "bmi z score" OR "bmi z score" OR "body mass index z-score" OR "body mass index z-score" OR "bmi percentile*" OR "bmi centile*" OR "body mass index percentile*" OR "body mass index centile*" OR "body weight" OR "body size" OR "weight*" OR "waist" OR "adipos*" OR "body composition" OR "overweight" OR “body weight changes” OR “healthy weight” OR “weight loss” OR “weight gain”)<br><br>**Limiters** - Age Groups: Infant, 1-23 months, Child, Preschool 2-5 years
#12	#1 AND #10 AND #11<br><br>**Limiters** - Publication Year: 2011-2023

For peer review only

Cochrane

#1<br>Population<br>TI-AB-KW	(preschool* OR pre-school* OR “pre-school*” OR kindergar?en OR prekinder* OR pre-Kinder* OR “pre Kinder*” OR headstart OR “head start” OR “surestart” OR “sure start” OR “family child care home” OR “family childcare home” OR “infant*” OR “toddler*” OR “child care” OR “child-care” OR childcare OR “early learning centre” OR “early child*” OR “early care and education” OR (child* AND “day care” OR “daycare” OR “day-care” OR playgroup* OR “nurser*” OR child day care centre*))
#2<br>Diet<br>TI-AB-KW	(diet* OR dietary OR food* OR snack* OR fruit* OR drink* OR beverage* OR juice* OR vegetable* OR meal* OR nutrition* OR intake* OR consum* OR "food quality" OR "food preference*" OR "energy density" OR "lifestyle behavio*r" OR "eating behavio*r" OR "healthy eating" OR "feeding behavio*r" OR "health promotion" OR "eating habit*" OR "food habit*" OR "food choice*" OR "overeat*" OR "over eat*" OR "weight control" OR "weight management" OR "menu planning" OR "menu choice*" OR "meal choice*" OR "meal*" OR "meal planning" OR (food* AND consum*) OR "eating habit*")
#3<br>PA<br>TI-AB-KW	(“physical* activ*” OR sport OR sports OR exercis* OR playtime OR sedentary OR exercise* OR play* OR “physical fitness” OR “movement” OR “playtime”)
#4<br>Outcome terms<br>TI-AB-KW	(obesity OR obes* OR "body mass index" OR BMI OR "bmi z score" OR "bmi z score" OR "body mass index z-score" OR "body mass index z-score" OR "bmi percentile*" OR "bmi centile*" OR "body mass index percentile*" OR "body mass index centile*" OR "body weight" OR "body size" OR weight* OR waist OR "adipos*" OR "body composition" OR overweight OR “body weight changes” OR “healthy weight” OR “weight loss” OR “weight gain”)
#5	#1 AND #6 AND #7
#6	("intervention*" OR "weight reduction programs" OR "program*" OR "programme*" OR "project*" OR "therapeutics" OR "scheme" OR "initiative*" OR "strateg*" OR "service*" OR "health education" OR "health promotion" OR "primary prevention" OR "health education" OR "health promotion" OR "primary prevention" or "treat*" OR therapy* or "pilot projects" OR "feasibility studies" OR "program evaluation")
#7	#5 AND #6

Web of Science

#1<br>Population<br>TS	TS=(preschool* OR pre-school* OR “pre-school*” OR kindergar$en OR prekinder* OR pre-Kinder* OR “pre Kinder*” OR headstart OR “head start” OR “surestart” OR “sure start” OR “family child care home” OR “family childcare home” OR “infant*” OR “toddler*” OR “child care” OR “child-care” OR childcare OR “early learning centre” OR “early child*” OR “early care and education” OR (child* AND “day care” OR “daycare” OR “day-care” OR playgroup* OR “nurser*” OR “child day care centre*”))
#2<br>Diet<br>TS	TS=((diet* OR dietary OR food* OR snack* OR fruit* OR drink* OR beverage* OR juice* OR vegetable* OR meal* OR nutrition* OR intake* OR consum* OR “food quality” OR “food preference*” OR “energy density” OR “lifestyle behavio$r” OR “eating behavio$r” OR “healthy eating” OR “feeding behavio$r” OR “health promotion” OR “eating habit*” OR “food habit*” OR “food choice*” OR “overeat*” OR “over eat*” OR “weight control” OR “menu planning” OR “menu choice*” OR “meal choice*” OR “meal*” OR “meal planning” OR (food* AND consum*) OR “eating habit*”))
#3<br>PA<br>TS	TS=((“physical* activ*” OR playtime OR sedentary OR exercise* OR play* OR “physical fitness” OR “movement” OR “playtime” OR sport*))
#4<br>Interventio ns<br>TS	TS=((“intervention*” OR “weight reduction program*” "program*" OR "programme*" OR "project*" OR "therapeutics" OR "scheme" OR "initiative*" OR "strateg*" OR "life style" OR "service*" OR “pilot project*” OR “feasibility stud*” OR “program evaluation” OR “health education*” OR “health promotion” OR “primary prevention” OR “treat*” OR “random*” OR “cluster random*” OR “trial*” OR “evaluat*” OR “effective*” OR “mixed methods” OR “impact*”))
#5	TS=(("intervention*" OR "weight reduction programs" OR "program*" OR "programme*" OR "project*" OR "therapeutics" OR "scheme" OR "initiative*" OR "strateg*" OR "service*" OR "health education" OR "health promotion" OR "primary prevention" OR "health education" OR "health promotion" OR "primary prevention" or "treat*" OR therapy* or "pilot projects" OR "feasibility studies" OR "program evaluation"))
#6	TS=(("random*" OR "cluster random*" OR "trial*" OR "evaluat*" OR "effective*" OR "mixed methods" OR "impact*" OR "Non-Randomized Controlled Trial*" OR "Pragmatic Clinical Trial" OR "controlled Clinical Trial*" OR "randomized controlled trial*" OR “randomized” OR “randomised” OR "clinical trial*" OR “randomly” OR "controlled study" OR "controlled trial*" OR "control group" OR "intervention group" OR "experimental group" OR "comparison group" OR "control school" OR "intervention school" OR "control community" OR "intervention community" OR "intervention condition" OR "control condition" OR "treatment group" OR "control participant" OR "quasi experimental design" OR "experimental condition" OR "treatment condition" OR "pilot projects" OR "feasibility studies" OR "program evaluation" OR "pre test" OR "post test" OR "quasi experiment*" OR blinded OR "double bind" OR fidelity OR reach OR uptake OR implementation OR "intervention effect*" OR "program effect*" OR "programme effect*" OR "proof of concept*" OR "outcome study"))
#7	#5 AND #6
#8	#2 OR #3
#9	#4 OR #7
#10	#8 AND #9

For peer review only

#11	TS=(("obesity" OR "obes*" OR "body mass index" OR "BMI" OR "bmi z score" OR "bmi z score" OR "body mass index z-score" OR "body mass index z-score" OR "bmi percentile*" OR "bmi centile*" OR "body mass index percentile*" OR "body mass index centile*" OR "body weight" OR "body size" OR "weight*" OR "waist" OR "adipos*" OR "body composition" OR "overweight" OR "body weight changes" OR "healthy weight" OR "weight loss" OR "weight gain"))
#12	#1 AND #10 AND #11<br>and 2023 and 2022<br>and 2021 or 2020 or 2019 or 2018 or 2017 or 2016 or 2015 or 2014 or 2013 or 2012 or 2011 (Publication Years)

For peer review only

SCOPUS

1	(TITLE-ABS ((preschool* OR pre-school* OR {pre-school*} OR kindergar?en OR prekinder* OR pre kinder* OR {pre Kinder} OR headstart OR {head start} OR surestart OR {sure start} OR {family child care home} OR {family childcare home} OR infant* OR toddler* OR {child care} OR {child-care} OR childcare OR {early learning centre} OR {early child} OR {early care and education} OR (child* AND {day care} OR daycare OR {day-care}) OR playgroup* OR nurser* OR {child day care centre*}))) AND (((TITLE-ABS ((diet* OR dietary OR food* OR snack* OR fruit* OR drink* OR beverage* OR juice* OR vegetable* OR meal* OR nutrition* OR intake* OR consum* OR {food quality} OR {food preference} OR {energy density} OR {lifestyle behavio?r} OR {eating behavio?r} OR {healthy eating} OR {feeding behavio?r} OR {health promotion} OR {eating habit*} OR {food habit*} OR {food choice*} OR {overeat*} OR {over eat*} OR {weight control} OR {menu planning} OR {menu choice} OR {meal choice} OR {meal*} OR {meal planning} OR {eating habit*}))) OR (TITLE-ABS (({physical* activ*} OR playtime OR sedentary OR exercise* OR play* OR {physical fitness} OR movement OR playtime OR sport*)))) AND ((TITLE-ABS (intervention* OR {weight reduction program*} OR program* OR programme* OR project* OR therapeutics OR scheme OR initiative OR strateg* OR lifestyle OR service* OR {pilot project*} OR {feasibility stud*} OR {program evaluation} OR {health education*} OR {health promotion} OR "treat*" OR {primary prevention} OR "random*" OR {cluster random*} OR trial* OR evaluat* OR effective* OR {mixed methods} OR impact*)) OR ((TITLE-ABS (intervention* OR {weight reduction programs} OR program* OR programme* OR project* OR therapeutics OR scheme OR initiative* OR strateg* OR service* OR {health education} OR {health promotion} OR {primary prevention} OR {health education} OR treat* OR therapy* OR {pilot projects} OR {feasibility studies} OR {program evaluation})) AND (TITLE-ABS (random* OR {cluster random*} OR trial* OR evaluat* OR effective* OR {mixed methods} OR impact* OR {Non-Randomized Controlled Trial*} OR {Pragmatic Clinical Trial} OR {controlled Clinical Trial*} OR {randomized controlled trial*} OR randomized OR randomised OR {clinical trial*} OR randomly OR {controlled study} OR {controlled trial*} OR {control group} OR {intervention group} OR {experimental group} OR {comparison group} OR {control school} OR {intervention school} OR {control community} OR {intervention community} OR {intervention condition} OR {control condition} OR {treatment group} OR {control participant} OR {quasi experimental design} OR {experimental condition} OR {treatment condition} OR {pilot projects} OR {feasibility studies} OR {program evaluation} OR {pre test} OR {post test} OR {quasi experiment*} OR blinded OR {double blind} OR fidelity OR reach OR uptake OR implementation OR {intervention effect*} OR {program effect*} OR {programme effect*} OR {proof of concept*} OR {outcome study}))))) AND (TITLE-ABS ((obesity OR obes* OR {body mass index} OR {BMI} OR {bmi z score} OR {bmi z score} OR {body mass index z-score} OR {body mass index z-score} OR {bmi percentile*} OR {bmi centile*} OR {body mass index percentile*} OR {body mass index centile*} OR {body weight} OR {body size} OR weight* OR waist OR adipos* OR {body composition} OR overweight OR {body weight changes} OR {healthy weight} OR {weight loss} OR {weight gain}))) AND (LIMIT-TO (PUBYEAR , 2023) OR LIMIT-TO (PUBYEAR , 2022) OR LIMIT-TO (PUBYEAR , 2021) OR LIMIT-TO (PUBYEAR , 2020) OR LIMIT-TO (PUBYEAR , 2019) OR LIMIT-TO (PUBYEAR , 2018) OR LIMIT-TO (PUBYEAR , 2017) OR LIMIT-TO (PUBYEAR , 2016) OR LIMIT-TO (PUBYEAR , 2015) OR LIMIT-TO (PUBYEAR , 2014) OR LIMIT-TO (PUBYEAR , 2013) OR LIMIT-TO (PUBYEAR , 2012) OR LIMIT-TO (PUBYEAR , 2011))

PsychINFO

**1**	(((preschool* or pre-school* or pre-school* or kindergar$en or prekinder* or pre-Kinder* or pre Kinder* or headstart or head start or surestart or sure start or family child care home or family childcare home or infant* or toddler* or child care or child-care or childcare or early learning centre or early child* or early care) and education) or ((child* and day care) or daycare or day-care or playgroup* or nurser* or child day care centre*)).mp. [mp=title, abstract, heading word, table of contents, key concepts, original title, tests & measures, mesh]
**2**	(diet* or dietary or food* or snack* or fruit* or drink* or beverage* or juice* or vegetable* or meal* or nutrition* or intake* or consum* or food quality or food preference* or energy density or lifestyle behavio?r or eating behavio?r or healthy eating or feeding behaviour?r or health promotion or eating habit* or food habit* or food choice* or overeat* or over eat* or weight control or menu planning or menu choice or meal choice* or meal* or meal planning or (food* and consum*) or eating habit*).mp. [mp=title, abstract, heading word, table of contents, key concepts, original title, tests & measures, mesh]
**3**	(physical* activ* or playtime or sedentary or exercise* or play* or physical fitness or movement or playtime or sport*).mp. [mp=title, abstract, heading word, table of contents, key concepts, original title, tests & measures, mesh]
**4**	(intervention* or weight reduction program* or program* or programme* or project* or therapeutics or scheme or initiative* or strateg* or life style or service* or pilot project* or feasibility stud* or program evaluation or health education* or health promotion or primary prevention or treat* or random* or cluster random* or trial* or evaluat* or effective* or mixed methods or impact*).mp. [mp=title, abstract, heading word, table of contents, key concepts, original title, tests & measures, mesh]
**5**	(intervention* or weight reduction programs or program*OR programme* or project* or therapeutics or scheme or initiative* or strateg* or service* or health education or health promotion or primary prevention or health education or health promotion or primary prevention or treat* or therapy* or pilot projects or feasibility studies or program evaluation).mp. [mp=title, abstract, heading word, table of contents, key concepts, original title, tests & measures, mesh]
**6**	(random* or cluster random* or trial* or evaluat* or effective* or mixed methods or impact* or Non-Randomized Controlled Trial* or Pragmatic Clinical Trial or controlled Clinical Trial* or randomized controlled trial* or randomized or randomised or clinical trial* or randomly or controlled study or controlled trial* or control group or intervention group or experimental group or comparison group or control school or intervention school or control community or intervention community or intervention condition or control condition or treatment group or control participant or quasi experimental design or experimental condition or treatment condition or pilot projects or feasibility studies or program evaluation or pre test or post test or quasi experiment* or blinded or double blind or fidelity or reach or uptake or implementation or intervention effect* or program effect* or programme effect* or proof of concept* or outcome study).mp. [mp=title, abstract, heading word, table of contents, key concepts, original title, tests & measures, mesh]
**7**	5 and 6
**8**	2 or 3
**9**	4 or 7
**10**	8 and 9
**11**	(obesity or obes* or body mass index or BMI or bmi z score or bmi z score or body mass index z-score or body mass index z-score or bmi percentile* or bmi centile* or body mass index percentile* or body mass index centile* or body weight or body size or weight* or waist or adipos* or body composition or overweight or body weight changes or healthy weight or weight loss or weight gain).mp. [mp=title, abstract, heading word, table of contents, key concepts, original title, tests & measures, mesh]
**12**	1 and 10 and 11

For peer review only

**13**	limit 12 to last 12 years

For peer review only

Social Policy and Practice

**1**	(((preschool* or pre-school* or pre-school* or kindergar$en or prekinder* or pre-Kinder* or pre Kinder* or headstart or head start or surestart or sure start or family child care home or family childcare home or infant* or toddler* or child care or child-care or childcare or early learning centre or early child* or early care) and education) or ((child* and day care) or daycare or day-care or playgroup* or nurser* or child day care centre*)).mp. [mp=abstract, title, publication type, heading word, accession number]
**2**	(diet* or dietary or food* or snack* or fruit* or drink* or beverage* or juice* or vegetable* or meal* or nutrition* or intake* or consum* or food quality or food preference* or energy density or lifestyle behavio?r or eating behavio?r or healthy eating or feeding behaviour?r or health promotion or eating habit* or food habit* or food choice* or overeat* or over eat* or weight control or menu planning or menu choice or meal choice* or meal* or meal planning or (food* and consum*) or eating habit*).mp. [mp=abstract, title, publication type, heading word, accession number]
**3**	(physical* activ* or playtime or sedentary or exercise* or play* or physical fitness or movement or playtime or sport*).mp. [mp=abstract, title, publication type, heading word, accession number]
**4**	(intervention* or weight reduction program* or program* or programme* or project* or therapeutics or scheme or initiative* or strateg* or life style or service* or pilot project* or feasibility stud* or program evaluation or health education* or health promotion or primary prevention or treat* or random* or cluster random* or trial* or evaluat* or effective* or mixed methods or impact*).mp. [mp=abstract, title, publication type, heading word, accession number]
**5**	(intervention* or weight reduction programs or program*OR programme* or project* or therapeutics or scheme or initiative* or strateg* or service* or health education or health promotion or primary prevention or health education or health promotion or primary prevention or treat* or therapy* or pilot projects or feasibility studies or program evaluation).mp. [mp=abstract, title, publication type, heading word, accession number]
**6**	(random* or cluster random* or trial* or evaluat* or effective* or mixed methods or impact* or Non-Randomized Controlled Trial* or Pragmatic Clinical Trial or controlled Clinical Trial* or randomized controlled trial* or randomized or randomised or clinical trial* or randomly or controlled study or controlled trial* or control group or intervention group or experimental group or comparison group or control school or intervention school or control community or intervention community or intervention condition or control condition or treatment group or control participant or quasi experimental design or experimental condition or treatment condition or pilot projects or feasibility studies or program evaluation or pre test or post test or quasi experiment* or blinded or double blind or fidelity or reach or uptake or implementation or intervention effect* or program effect* or programme effect* or proof of concept* or outcome study).mp. [mp=abstract, title, publication type, heading word, accession number]
**7**	5 and 6
**8**	2 or 3
**9**	4 or 7
**10**	8 and 9
**11**	(obesity or obes* or body mass index or BMI or bmi z score or bmi z score or body mass index z-score or body mass index z-score or bmi percentile* or bmi centile* or body mass index percentile* or body mass index centile* or body weight or body size or weight* or waist or adipos* or body composition or overweight or body weight changes or healthy weight or weight loss or weight gain).mp. [mp=abstract, title, publication type, heading word, accession number]
**12**	1 and 10 and 11
**13**	limit 12 to last 12 years

For peer review only

HMIC Health Management Information Consortium

**1**	(((preschool* or pre-school* or pre-school* or kindergar$en or prekinder* or pre-Kinder* or pre Kinder* or headstart or head start or surestart or sure start or family child care home or family childcare home or infant* or toddler* or child care or child-care or childcare or early learning centre or early child* or early care) and education) or ((child* and day care) or daycare or day-care or playgroup* or nurser* or child day care centre*)).mp. [mp=title, other title, abstract, heading words]
**2**	(diet* or dietary or food* or snack* or fruit* or drink* or beverage* or juice* or vegetable* or meal* or nutrition* or intake* or consum* or food quality or food preference* or energy density or lifestyle behavio?r or eating behavio?r or healthy eating or feeding behaviour?r or health promotion or eating habit* or food habit* or food choice* or overeat* or over eat* or weight control or menu planning or menu choice or meal choice* or meal* or meal planning or (food* and consum*) or eating habit*).mp. [mp=title, other title, abstract, heading words]
**3**	(physical* activ* or playtime or sedentary or exercise* or play* or physical fitness or movement or playtime or sport*).mp. [mp=title, other title, abstract, heading words]
**4**	(intervention* or weight reduction program* or program* or programme* or project* or therapeutics or scheme or initiative* or strateg* or life style or service* or pilot project* or feasibility stud* or program evaluation or health education* or health promotion or primary prevention or treat* or random* or cluster random* or trial* or evaluat* or effective* or mixed methods or impact*).mp. [mp=title, other title, abstract, heading words]
**5**	(intervention* or weight reduction programs or program*OR programme* or project* or therapeutics or scheme or initiative* or strateg* or service* or health education or health promotion or primary prevention or health education or health promotion or primary prevention or treat* or therapy* or pilot projects or feasibility studies or program evaluation).mp. [mp=title, other title, abstract, heading words]
**6**	(random* or cluster random* or trial* or evaluat* or effective* or mixed methods or impact* or Non-Randomized Controlled Trial* or Pragmatic Clinical Trial or controlled Clinical Trial* or randomized controlled trial* or randomized or randomised or clinical trial* or randomly or controlled study or controlled trial* or control group or intervention group or experimental group or comparison group or control school or intervention school or control community or intervention community or intervention condition or control condition or treatment group or control participant or quasi experimental design or experimental condition or treatment condition or pilot projects or feasibility studies or program evaluation or pre test or post test or quasi experiment* or blinded or double blind or fidelity or reach or uptake or implementation or intervention effect* or program effect* or programme effect* or proof of concept* or outcome study).mp. [mp=title, other title, abstract, heading words]
**7**	5 and 6
**8**	2 or 3
**9**	4 or 7
**10**	8 and 9
**11**	(obesity or obes* or body mass index or BMI or bmi z score or bmi z score or body mass index z-score or body mass index z-score or bmi percentile* or bmi centile* or body mass index percentile* or body mass index centile* or body weight or body size or weight* or waist or adipos* or body composition or overweight or body weight changes or healthy weight or weight loss or weight gain).mp. [mp=title, other title, abstract, heading words]
**12**	1 and 10 and 11
**13**	limit 12 to last 12 years

For peer review only

Trials Register of Promoting Health Interventions (TRoPHI)

saved 551 records from the following searches:

Search 1:

2343: 2341 AND 2342 361 records (of which saved records from year 2011 onwards)

2342: Coded with: children (0-10 yrs) 1933

2341: Coded with: obesity 1194

Search 2:

2366: 2365 AND 2329 (unscreened records): 123 records

2365: 2364 AND 2347 1116

2347: "toddler* OR young child* OR preschool* OR infant* OR child*" (in Title and Abstract)

2364: Coded with: Weight Loss, OR Physical Activity

Search 3:

2344: AND 2347 AND 2355 AND 2338: 221 records

2344: Not coded with: EPPI-Centre Health promotion keywording strategy

2347: "toddler* OR young child* OR preschool* OR infant* OR child*" (in Title and Abstract)

2355: "obes* OR sedentar* OR physical* OR diet* OR exercise OR BMI OR "body weight" OR "body size" OR waist " (in Title and Abstract)

2338: Coded with: Include: Randomised controlled trial, OR Include: Non-Random 14391

Search 4: 136 records: (of which saved records from year 2010 onwards)

"obes* OR sedentar* OR physical* OR diet*" (in Title and Abstract)

AND

"exercise OR BMI OR "body weight" OR "body size" OR waist " (in Title and Abstract)

AND

"toddler* OR young child* OR preschool* OR infant* OR child*" (in Title and Abstract)

AND

Not coded with: EPPI-Centre Health promotion keywording strategy

AND

Not coded with: Ex 1: Not Health Promotion or Public Health, OR Ex 2: Not Human or Environment, OR Ex 3: NOT an evaluation of an intervention, OR Ex: 4 No control or comparison group, OR Ex 5: Item is a review or meta-analysis, OR MARKER: QUERY, OR Duplicate

For peer review only

Appendix 2

Depiction of the screening process

The graph below depicts the screening process on tittle and abstract. The prioritisation of records was refreshing regularly. The most relevant articles were screened first. The screening stopped when the graph reached a plateau.

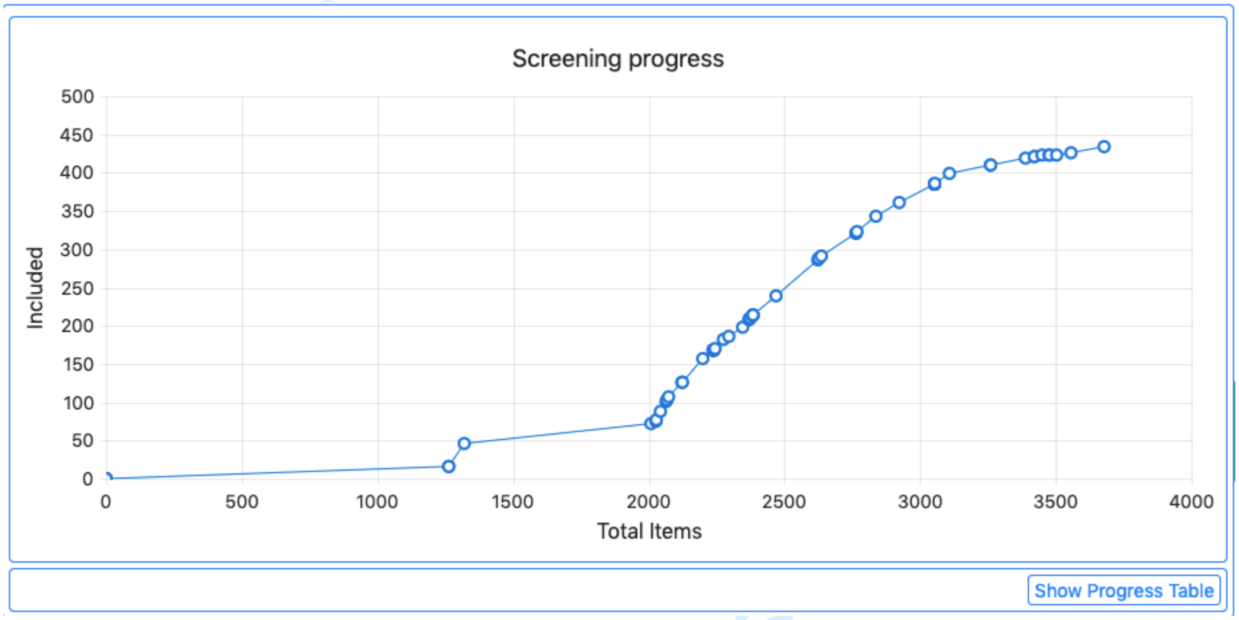

Appendix 3

Bias assessment

Table S2: Studies assessed using the ROB tool

	Randomisation bias	Adhering to the intervention	Missing data	Outcome measurement	Reporting bias	Overall
Hodgkinson 2019	High	High	Low	Low	Low	High
Malden 2019	Moderate	High	Low	Low	Low	High
Bryant 2021	Moderate	High	Low	Low	Low	High
Lanigan (unpublished)	Low	Low	High	Low	High	High

- Red: High risk of bias
- Amber: Moderate risk of bias
- Green: Low risk of bias

Table S3: Studies assessed using the ROBINS-I tool

	Confounding bias	Selection of participants bias	Classification of interventions bias	Adherence to the intervention	Missing data	Outcome measurement	Reporting bias	Overall
Lee 2021	Low	Moderate	Low	Moderate	High	Low	Low	High

For peer review only

PRISMA 2020 Checklist

Section and Topic	Item #	Checklist item	Location where item is reported
**TITLE**			
Title	1	Identify the report as a systematic review.	Lines 1-2
**ABSTRACT**			
Abstract	2	See the PRISMA 2020 for Abstracts checklist.	Lines 16-40
**INTRODUCTION**			
Rationale	3	Describe the rationale for the review in the context of existing knowledge.	Lines 93-100
Objectives	4	Provide an explicit statement of the objective(s) or question(s) the review addresses.	Lines 72-79
**METHODS**			
Eligibility criteria	5	Specify the inclusion and exclusion criteria for the review and how studies were grouped for the syntheses.	Lines 115-128 & Lines 152-155
Information sources	6	Specify all databases, registers, websites, organisations, reference lists and other sources searched or consulted to identify studies. Specify the date when each source was last searched or consulted.	Lines 106-114 & Appendix 1
Search strategy	7	Present the full search strategies for all databases, registers and websites, including any filters and limits used.	Appendix 1
Selection process	8	Specify the methods used to decide whether a study met the inclusion criteria of the review, including how many reviewers screened each record and each report retrieved, whether they worked independently, and if applicable, details of automation tools used in the process.	Lines 129-147
Data collection process	9	Specify the methods used to collect data from reports, including how many reviewers collected data from each report, whether they worked independently, any processes for obtaining or confirming data from study investigators, and if applicable, details of automation tools used in the process.	Lines 141-147
Data items	10a	List and define all outcomes for which data were sought. Specify whether all results that were compatible with each outcome domain in each study were sought (e.g. for all measures, time points, analyses), and if not, the methods used to decide which results to collect.	Lines 155-162
	10b	List and define all other variables for which data were sought (e.g. participant and intervention characteristics, funding sources). Describe any assumptions made about any missing or unclear information.	Lines 142-147
Study risk of bias assessment	11	Specify the methods used to assess risk of bias in the included studies, including details of the tool(s) used, how many reviewers assessed each study and whether they worked independently, and if applicable, details of automation tools used in the process.	Lines 163-167
Effect measures	12	Specify for each outcome the effect measure(s) (e.g. risk ratio, mean difference) used in the synthesis or presentation of results.	Lines 155-157
Synthesis methods	13a	Describe the processes used to decide which studies were eligible for each synthesis (e.g. tabulating the study intervention characteristics and comparing against the planned groups for each synthesis (item #5)).	Lines 152-157
	13b	Describe any methods required to prepare the data for presentation or synthesis, such as handling of missing summary statistics, or data conversions.	NA
	13c	Describe any methods used to tabulate or visually display results of individual studies and syntheses.	NA
	13d	Describe any methods used to synthesize results and provide a rationale for the choice(s). If meta-analysis was performed, describe the model(s), method(s) to identify the presence and extent of statistical heterogeneity, and software package(s) used.	Lines 152-155
	13e	Describe any methods used to explore possible causes of heterogeneity among study results (e.g. subgroup analysis, meta-regression).	NA
	13f	Describe any sensitivity analyses conducted to assess robustness of the synthesized results.	NA
Reporting bias assessment	14	Describe any methods used to assess risk of bias due to missing results in a synthesis (arising from reporting biases).	NA

For peer review only - http://bmjopen.bmj.com/site/about/guidelines.xhtml

PRISMA 2020 Checklist

Section and Topic	Item #	Checklist item	Location where item is reported
Certainty assessment	15	Describe any methods used to assess certainty (or confidence) in the body of evidence for an outcome.	NA
**RESULTS**			
Study selection	16a	Describe the results of the search and selection process, from the number of records identified in the search to the number of studies included in the review, ideally using a flow diagram.	Lines 177-188 & Figure 1
	16b	Cite studies that might appear to meet the inclusion criteria, but which were excluded, and explain why they were excluded.	Figure 1
Study characteristics	17	Cite each included study and present its characteristics.	Lines 187-200 & Table 1
Risk of bias in studies	18	Present assessments of risk of bias for each included study.	Appendix 3
Results of individual studies	19	For all outcomes, present, for each study: (a) summary statistics for each group (where appropriate) and (b) an effect estimate and its precision (e.g. confidence/credible interval), ideally using structured tables or plots.	Table 1
Results of syntheses	20a	For each synthesis, briefly summarise the characteristics and risk of bias among contributing studies.	Lines 202-258
	20b	Present results of all statistical syntheses conducted. If meta-analysis was done, present for each the summary estimate and its precision (e.g. confidence/credible interval) and measures of statistical heterogeneity. If comparing groups, describe the direction of the effect.	NA
	20c	Present results of all investigations of possible causes of heterogeneity among study results.	NA
	20d	Present results of all sensitivity analyses conducted to assess the robustness of the synthesized results.	NA
Reporting biases	21	Present assessments of risk of bias due to missing results (arising from reporting biases) for each synthesis assessed.	NA
Certainty of evidence	22	Present assessments of certainty (or confidence) in the body of evidence for each outcome assessed.	NA
**DISCUSSION**			
Discussion	23a	Provide a general interpretation of the results in the context of other evidence.	Lines 270-277
	23b	Discuss any limitations of the evidence included in the review.	Lines 282-288 & Lines 311-319
	23c	Discuss any limitations of the review processes used.	Lines 337-344
	23d	Discuss implications of the results for practice, policy, and future research.	Lines 345-353
**OTHER INFORMATION**			
Registration and protocol	24a	Provide registration information for the review, including register name and registration number, or state that the review was not registered.	Lines 102-105
	24b	Indicate where the review protocol can be accessed, or state that a protocol was not prepared.	Lines 102-105
	24c	Describe and explain any amendments to information provided at registration or in the protocol.	NA
Support	25	Describe sources of financial or non-financial support for the review, and the role of the funders or sponsors in the review.	Lines 50-55
Competing interests	26	Declare any competing interests of review authors.	Line 56
Availability of data, code and	27	Report which of the following are publicly available and where they can be found: template data collection forms; data extracted from included studies; data used for all analyses; analytic code; any other materials used in the review.	NA

For peer review only

For peer review only - http://bmjopen.bmj.com/site/about/guidelines.xhtml

PRISMA 2020 Checklist

Section and Topic	Item #	Checklist item	Location where item is reported
other materials			

From: Page MJ, McKenzie JE, Bossuyt PM, Boutron I, Hoffmann TC, Mulrow CD, et al. The PRISMA 2020 statement: an updated guideline for reporting systematic reviews. BMJ 2021;372:n71. doi: 10.1136/bmj.n71

For more information, visit: http://www.prisma-statement.org/

For peer review only

BMJ Open

Assessing the impact of obesity interventions in the early years: A systematic review of UK-based studies

Journal:	*BMJ Open*
Manuscript ID	bmjopen-2023-076479.R2
Article Type:	Original research
Date Submitted by the Author:	06-Feb-2024
Complete List of Authors:	Michalopoulou, Semina; University College London, Great Ormond Street Institute of Child Health<br>Sifaki, Maria; University College London, Great Ormond Street Institute of Child Health<br>Packer, Jessica; University College London, Great Ormond Street Institute of Child Health<br>Lanigan, Julie; University College London, Great Ormond Street Institute of Child Health<br>Stansfield, Claire; University College London, Institute of Education<br>Viner, Russell; University College London, Great Ormond Street Institute of Child Health<br>Russell, Simon; University College London, Great Ormond Street Institute of Child Health
**Primary Subject Heading**:	Paediatrics
Secondary Subject Heading:	Health policy, Public health
Keywords:	Obesity, NUTRITION & DIETETICS, Community child health < PAEDIATRICS

SCHOLARONE™
Manuscripts

I, the Submitting Author has the right to grant and does grant on behalf of all authors of the Work (as defined in the below author licence), an exclusive licence and/or a non-exclusive licence for contributions from authors who are: i) UK Crown employees; ii) where BMJ has agreed a CC-BY licence shall apply, and/or iii) in accordance with the terms applicable for US Federal Government officers or employees acting as part of their official duties; on a worldwide, perpetual, irrevocable, royalty-free basis to BMJ Publishing Group Ltd ("BMJ") its licensees and where the relevant Journal is co-owned by BMJ to the co-owners of the Journal, to publish the Work in this journal and any other BMJ products and to exploit all rights, as set out in our licence.

The Submitting Author accepts and understands that any supply made under these terms is made by BMJ to the Submitting Author unless you are acting as an employee on behalf of your employer or a postgraduate student of an affiliated institution which is paying any applicable article publishing charge ("APC") for Open Access articles. Where the Submitting Author wishes to make the Work available on an Open Access basis (and intends to pay the relevant APC), the terms of reuse of such Open Access shall be governed by a Creative Commons licence – details of these licences and which Creative Commons licence will apply to this Work are set out in our licence referred to above.

Other than as permitted in any relevant BMJ Author's Self Archiving Policies, I confirm this Work has not been accepted for publication elsewhere, is not being considered for publication elsewhere and does not duplicate material already published. I confirm all authors consent to publication of this Work and authorise the granting of this licence.

For peer review only

Assessing the impact of obesity interventions in the early years: A systematic review of UK-based studies

Semina Michalopoulou,[1] Maria Sifaki,[1] Jessica Packer,[1] Julie Lanigan,[1] Clare Stansfield,[2] Russell Viner[1] and Simon Russell[1]

[1] UCL Great Ormond Street Institute of Child Health, University College London, London, UK,

[2] UCL Institute of Education, University College London, London, UK

Corresponding author:

Simon Russell

UCL Great Ormond Street Institute of Child Health, University College London, 30 Guilford Street, London, WC1N 1EH

Tel: 0207 905 2110

s.russell@ucl.ac.uk

Short version of the title: UK early childhood obesity interventions

Keywords: childhood obesity, interventions, diet, physical activity, early years, United Kingdom

For peer review only

Abstract

Objectives: Childhood obesity rates in the UK are high. The early years of childhood are critical for establishing healthy behaviours and offer interventional opportunities. We aimed to identify studies evaluating the impact of UK-based obesity interventions in early childhood.

Design: Systematic review using the PRISMA guidelines

Data sources: Nine databases were searched in March 2023. Eligibility criteria: We included UK-based obesity intervention studies delivered to children aged 6-months to five years, that had diet and/or physical activity components and reported anthropometric outcomes. The primary outcome of interest was zBMI change (within and between subjects). Studies evaluating the effects of breastfeeding interventions were not included as obesity prevention interventions, given that best practice formula feeding is also likely to encourage healthy growth. The publication date for studies was limited to the previous 12 years (2011-2023), as earlier reviews found few evaluations of interventions in the UK.

Data extraction and synthesis: The reviewers worked independently using standardised approach to search, screen and code the included studies. Risk of bias was assessed using Cochrane tools (ROB 2 or ROBINS-I).

Results: Six trials (five studies) were identified, including two randomised controlled trials (RCT), one cluster randomised trial (CRT), two feasibility CRTs, and one impact assessment. The total number of participants was 566. Three trials focused on disadvantaged families and two included high-risk children categorised as having overweight or obesity. Compared to baseline, five interventions reported reductions in zBMI, three of which were statistically significant ($p<0.05$). Compared to control, five interventions showed zBMI reductions, one of which was

For peer review only

significant. Only two trials were followed up beyond 12 months. All studies were found to have a high risk of bias. Meta-analysis was not possible due to the heterogeneity of studies.

Conclusion: UK evidence was limited but some interventions showed promising results in promoting healthy growth. As part of a programme of policies, interventions in the early years may have an important role in reducing the risk of childhood obesity.

PROSPERO registration number: CRD42021290676

Strengths and limitations of this study:

- A key strength was that searches were comprehensive and were conducted across nine scientific databases.
- For this review, we used specialist software (EPPI Reviewer Web), which was both a strength and limitation. The software utilised 'active learning' to enable priority screening, which greatly reduced screening time but resulted in a proportion of studies being excluded without being screened.
- A limitation of our approach was that we excluded studies published prior to 2011; however, previous research suggests little to no evaluations of UK obesity interventions in the early years prior to this time.
- A further limitation was that evaluations of interventions delivered outside the UK were excluded; however, the work has high relevance to UK contexts, particularly in informing policy and practice.
- A final limitation was that we were unable to perform meta-analysis due to the heterogeneity of the interventions.

For peer review only

Funding statement: This study was funded by the UK National Institute for Health and Care Research Policy Research Programme (PR-PRU-0916-21001; grant number 174868). The views expressed in this Article are those of the authors and not necessarily those of the National Institute for Health and Care Research or the UK Department of Health and Social Care.

Competing interests statement: None

For peer review only

INTRODUCTION

In England, data from the National Child Measurement Programme (NCMP) show that the prevalence of obesity among children aged 4-5 years, though plateaued, remains stably high. Disadvantaged children are the worst affected, as the inequalities in childhood obesity are increasing.(1,2) Once established, obesity tracks strongly through childhood, adolescence, and into adulthood where it is associated with increased risk of physical and mental health morbidities.(3–7) Prospective evidence indicates that half of the adolescents with obesity had being living with overweight or obesity since the age of five. (8) Data from the NCMP suggest that the odds of children with overweight in the age of 4-5 years developing obesity by 10 and 11 years were 13 times higher compared to children with healthy BMI.(9) Longitudinal evidence shows that a child's weight status remains stable through primary school years, as 68% of children with obesity and 78% of children with healthy weight at 4 and 5 years maintained their weight status by 10 and 11 years.(9) The early years of life are therefore a critical period for the development of a healthy weight, representing a window of opportunity for the introduction of a healthy lifestyle to support healthy growth.

Diet(10,11) and physical activity(12) are the major determinants of weight in young children. The diets of many pre-schoolers in the UK do not adhere to government dietary guidelines, exceeding intake of free sugar and saturated fat, and not reaching recommendations for fruit and vegetables.(13,14) Physical activity levels are reported to be below recommended guidelines(15) with most children not engaging in the 180 minutes of physical activity per day recommended by the National Health Service (NHS).(16) Lifestyle behaviours track through childhood, into adolescence, and adulthood, meaning that those children are likely to continue

For peer review only

following unhealthy practices, thereby being at even greater risk of overweight and obesity.(17,18)

Considering the significance of the first five years for outcomes later in life, interventions to support the establishment of a healthy weight and a healthy lifestyle are valuable. In the UK, the National Institute for Health and Care Excellence (NICE)(19) gives guidance on the key interventional components to reduce the risk or manage overweight and obesity. Principally, these include the adoption of a healthy diet, addressing lifestyle risk factors within the family and social settings, i.e., preschools, reducing sedentary lifestyle, and supporting behavioural change. The UK Government's Tackling Obesity Policy Paper(20) aims to place prevention at the heart of the health agenda to proactively tackle the burden of obesity for adults and children. The Paper suggests that an integrated, whole systems approach is likely to be the most effective in addressing the complex pathways to obesity. It is acknowledged that controlling the problem of childhood obesity is a shared responsibility of the Government, local authorities, and the NHS, in addition to the food industry, schools, and local communities.(20) Preventative and treatment interventions for obesity in early childhood can occur in various settings, typically family homes, nurseries, pre-school, or healthcare settings. Interventions in community settings, such as nurseries or pre-schools, are likely to be preventative and universal (for all children), and have the potential to reach more children.(21,22)

The high prevalence of childhood obesity in the UK supports the need for early interventions. To inform such interventions it is crucial to understand which interventions have been successful. It is also important to identify interventions with longer follow-up periods. The aim of this review was to synthesise evidence of interventions implemented and evaluated in the

For peer review only

UK that aim to prevent or treat obesity in children aged 6 months to five years. We restricted this review to UK interventions as early childhood environments can differ markedly across countries. Patient and public involvement workshops were conducted to help inform our approach and interpret findings.

MATERIALS AND METHODS

Design

This systematic review adhered to the Preferred Reporting Items for Systematic Reviews and Meta-Analyses (PRISMA) statement(23) and presents the UK findings of the PROSPERO-registered protocol CRD42021290676.

Search Strategy

In November 2021, we searched nine databases for obesity interventions among children under five years old published in peer reviewed journals (selected databases and search terms used are shown in Appendix 1). The searches were updated in March 2023. In addition to database searches, the authors searched reference lists of similar reviews. No language restrictions were applied. Our searches included international literature; however, here we report findings from UK studies only. Applicability and transferability of the interventions conducted elsewhere might be limited due to variability in obesity prevalence, resource availability, policy landscape, and delivery of the programmes.(24)

Inclusion and exclusion criteria

Primary research studies were included if they fulfilled the following criteria:

Population: children aged 6 months-5 years.

For peer review only

128 Intervention: Pre- and post-intervention studies including randomised controlled trials, quasi-
129 experimental studies, (individual, family- or community-based).
130 Comparison: pre- vs post-intervention and/or intervention vs control group.
131 Outcomes: at least one anthropometric outcome (e.g., BMI, BMI z-score, BMI percentile, waist
132 circumference, skinfold measurements). Anthropometric measurements were chosen as an
133 outcome because these are the most robust way of assessing impact, compared to other
134 potential outcomes (e.g., diet or physical activity).
135 The search was limited to studies published during the previous 12 years (2011-2023) to reflect
136 current or recent interventions and programmes, to understand what works in the current
137 context. Previous reviews found no studies conducted in the UK, meaning it is unlikely that
138 there were UK interventions robustly evaluated prior to 2011.(25,26)

139 **Selection of studies and data extraction**

140 Search results were imported into Endnote, where duplicates were removed, before being
141 imported into EPPI Reviewer Web,(27) in which further duplicate searches were undertaken.
142 Two reviewers (SM and MS) independently double screened on title and abstract. Using EPPI-
143 Reviewer software, we applied an ‘active learning approach’, where the prioritisation of records
144 was periodically refreshed during screening so that the most relevant articles were screened
145 first.(28) A graphical output was used to indicate when to stop screening, i.e., when the number
146 of relevant studies had plateaued (Appendix 2). A classifier model was then built using the
147 machine learning algorithm and applied to unscreened items, which generates a score (0-100)
148 indicating relevance; this process reduces the likelihood that relevant studies would be missed.
149 Reviewers independently screened all records with a score higher than 30.

For peer review only

Searches included children aged 0-5 years, interventions that targeted infant feeding in the first 6 months of life were excluded. Evidence suggests that breastfeeding is optimal in terms of growth, development, maturation of the immune system and programming of the metabolic system, and that formula feeding can be associated with rapid weight gain and later risk of obesity.(29) However, the association is complex and there is research that suggests best practice formula feeding can reduce rapid weight gain.(30) For this reason and owing to the complexity of the evidence, we did not include interventions that promote breastfeeding as obesity prevention interventions. The aim of this work was to assess the evidence of interventions promoting health growth in the early years for both breast and formula fed children. Reviewers developed a data extraction tool and data were doubly extracted. The data extracted included information on trial identification (name of study, authors, year of publication), country (country the trial took place and country of research), trial description, trial duration and follow-up, trial characteristics (preventative/treatment, intervention/programme), participants' characteristics (numbers, ages), and results regarding changes on zBMI. The reviewers double screened independently, and any disagreements were resolved through discussion.

Data synthesis

We performed a narrative synthesis of findings, summarising the effect estimates; meta-analysis was not possible owing to heterogeneity in intervention approaches, i.e., components of the interventions, participants targeted, site of delivery, duration, intensity, and length of follow-up. The included studies are presented in two sections: "preventative" or "treatment", based on their inclusion criteria. Universal interventions with children of any weight status are

For peer review only

included in the "preventative" category, while interventions that recruited children with overweight or obesity only are classified as "treatment" interventions. We report the mean difference of any anthropometric outcomes both between-group (i.e., intervention vs. control), and within-group (i.e., before vs. after). Measurements obtained at any time point (e.g., mid-intervention, right after the intervention, after the end of the intervention) were considered for this review. If studies provided the mean value of anthropometric outcomes before and after the intervention or in the control and the intervention group, two reviewers (SM &MS) calculated the mean difference and the standard deviation of the change using formulas that are suggested by the Cochrane Handbook.(31) The alpha level of significance was $p<0.05$.

Risk of bias assessment

Two reviewers (SM and MS) independently assessed the bias using the Cochrane risk of bias assessment tool (RoB 2) to evaluate the included randomised trials.(32) For one study that was an impact assessment, the ROBINS-I tool was used which is suitable for non-randomised studies.(33) Detailed bias assessment can be found in Appendix 3.

Patient and Public Involvement

We conducted meaningful patient and public engagement to integrate the voice of parents throughout this study. Through the National's Children's Bureau (NCB) we recruited parents with relevant lived experience from the Family Research Advisory Group panel (30). Parents from this panel have received training and understand research approaches and have experience of contributing insights and advice to research projects. Six members of the public (five female and one male) agreed to contribute to this study, all were or had been parents of young children, two were also grandparents. Public contributors were diverse in terms of age

For peer review only

and were recruited from across England. We held two online sessions with the same parents, one at the outset of the project, to incorporate views into our general approach, and one at the end, to share and discuss interpretations of the research. Specifically, we discussed current practices and evidence of obesity interventions for young children in the UK, what parents perceived to be the effective elements or components of interventions, the challenges of implementing effective interventions and for hard-to-reach groups, how to effectively evaluate interventions in the early years, and the role of parents in supporting interventions that promote healthy growth in early childhood.

RESULTS

Description of the studies

In total, the literature search yielded 34,572 potentially relevant articles. After duplicates removal, 21,547 articles were eligible for screening. Two independent reviewers (SM & MS) manually screened 9,538 records, in title and abstract, of which 6,217 were manually removed (Figure 1). Any differences were resolved by reconciliation and, if necessary, in consultations with the other authors. An additional 12,004 records were removed based on title and abstract, as indicated by the machine learning algorithm. A total of 3,326 records were included for full-text screening, two of which could not be retrieved, as we could not gain access to them through the University of London Library. Out of the 3,324 remaining records, 3,108 were manually excluded (reasons for exclusion presented in Figure 1), leaving 217 relevant studies. Out of the 217 international studies, five studies (six trials) were conducted in the UK and were included in the final review.(34–38)

For peer review only

One study was conducted in Lancashire,(34) one in Glasgow,(35) one in two areas in North and Central England, (36) one in Cornwall,(37) and one in Hertfordshire(38). There were two randomised controlled trials,(38) one cluster randomised trial (CRT),(34) two feasibility CRTs,(35,36) and one before/after impact assessment of a weight management programme.(37) All studies described interventions with both diet and physical activity outcomes. Sample sizes ranged from 42 to 117. Intervention duration ranged from eight weeks to 12 months. The follow-up measurements were obtained during the intervention for one study(35) and after the intervention for the rest, ranging from 6 to 30 months from the start of the intervention. Two trials targeted children living with overweight, obesity, or at high risk, (37)'(38) while the remaining four were preventative interventions,(34–36,38) and targeted children of any weight status (children in the general population, not necessarily living with overweight or obesity). Detailed information for each of the included trials can be found in Table 1.

[Figure 1]

Intervention characteristics and effects

Preventative interventions

The four preventative trials include the *Be active, Eat healthy/Healthy Heroes*(34) intervention, the *ToyBox*(35) intervention, the *Health Exercise Nutrition for the Really Young (HENRY)*(36) programme, and the *Planet Munch* programme, trial 2.(38)

Three out of four trials (Be Active, Eat healthy/Healthy Heroes, ToyBox, HENRY) focused on disadvantaged families or deprived areas.(34–36) Most (34–36)(Be Active, Eat healthy/Healthy Heroes, ToyBox, HENRY) were administered by preschool staff, which had been appropriately

trained, while one (Planet Munch)(38) was delivered by a trained multidisciplinary team including dietitians, nutritionists, community artists, and children's centre staff. Two interventions targeted and made changes in preschools' policies; for example, by informing the food policy(34) (Be Active, Eat healthy/Healthy Heroes) or by increasing the time allocated to physical activity (ToyBox).(35) Interventions also targeted the home environment, for example by providing healthy cooking sessions(34,38) (Be Active, Eat healthy/Healthy Heroes, Planet Munch) or sending materials for home-based physical activities. (ToyBox, Planet Munch).(35,38) One study(36) (HENRY) was delivered to parents through workshops organized in early years centres, with recommendations provided on nutrition, physical activity, and parenting. Planet Munch(38) included workshops for parents and children, that aimed to help them improve their dietary and physical activity practices and adopt a healthier lifestyle.

All studies involved a control group; in the majority of cases, the control group received standard care (Be Active, Eat healthy/Healthy Heroes, ToyBox, HENRY),(34–36) while in one study (Planet Munch),(38) the control group was a delayed intervention group. The risk assessment revealed that *Be active, eat healthy/Healthy Heroes, ToyBox, HENRY and Planet Munch* had a high-risk of bias, with the most studies having high or moderate risk of bias in the randomisation process and the intervention adherence (Appendix 3).

Only one trial (Be Active, Eat healthy/Healthy Heroes)(34) found the intervention group to have significantly decreased their zBMI after the intervention, compared to the control group [-0.74, (-1.10 to -0.38)]. Two studies(36,38) also found a lower zBMI in the intervention compared to the control group; however, there was either no statistical comparison between them (HENRY),(36) or the difference was not statistically significant (Planet Munch).(38) In one

For peer review only

trial(35) (ToyBox) the zBMI had been raised for both groups; nonetheless, this increase was larger for the control group (Table 1).

When looking at zBMI changes within the intervention group, three studies showed reductions, ranging from -0.02 (-0.2 to 0.2) to -0.9 (95% CIs not given) (Be Active, Eat healthy/Healthy Heroes, HENRY, Planet Munch).(34,36,38) In one study, this reduction was statistically significant (Be Active, Eat healthy/Healthy Heroes),(34) in one no statistical comparison was reported (HENRY),(36) and one study found the reduction not to be significant (Planet Munch).(38) Finally, one trial (Toy Box)(35) reported zBMI to have increased by 0.02 (-0.11 to 0.15). A second follow-up measurement took place in one of the studies (Planet Munch);(38) while there was a decrease compared to baseline, this was not significant (Table 1).

[Table 1]

Overweight and obesity treatment interventions

We identified two treatment trials. The first was *The Lifestyles, Eating, and Activity for Families (LEAF)*(37) programme, which was an impact assessment of a childhood obesity service and the treatment trial of *Planet Munch*.(38) *The LEAF programme* targeted children with severe obesity or severe obesity with a relevant comorbidity. The programme consisted of an initial home visit, workshops, and follow-up clinic appointments. A community paediatrician, a specialist dietitian, and a physical activity advisor were involved for each child. There was no control group in LEAF. In terms of within-subject changes, the *LEAF* programme found a significant reduction of zBMI, following programme participation (Table 1). The trial was assessed to have a high risk of bias mainly due to missing data.

For peer review only

The treatment trial of *Planet Munch* targeted children with overweight/obesity, or who were high-risk. As with the prevention trial, it was delivered by a multi-disciplinary team through healthy lifestyle workshops. Planet Munch included a control group (delayed intervention group). At the end of the intervention, after accounting for age and gender, the intervention group had lower mean zBMI score than the control group. For Planet Munch, there was a significant reduction in the zBMI of the intervention group, from baseline to the end of the intervention, as well as from baseline to the 2-year follow-up (Table 1)

Patient and Public Involvement

Parents, acting as public contributors, were mixed in their views around the potential impact and effectiveness of obesity interventions in early childhood. Half (n=3) felt that interventions in the early years could be effective, while half were sceptical. All parents thought that preventative interventions had the potential to have the greatest impact and the majority (n=5) were in favour of multi-component interventions that incorporated dietary and physical activity components, giving that the goal in early life is to encourage healthy lifestyles. Parents reported mixed views in relation to intervention components; three parents favoured educational interventions around living healthier lifestyles, two favoured motivational approaches, and one favoured addressing environmental factors to enable healthy growth among children. In terms of the interpretation of findings, all parents were surprised at how few interventions with robust evaluation there were in the UK. All parents reported that the cost of living a healthy lifestyle (e.g., buying fresh food) was an important factor and suggested there should be financial help or subsidies for parents of young children, particularly families with low income. There was consensus among parents that interventions should be properly evaluated and

For peer review only

better funded to ensure they are robust; for example, by following participants up over longer time frames. There was also consensus among parents that families, early years practitioners (e.g., health visitors) and parents of children living with excess weight should be consulted when developing interventions in early childhood.

DISCUSSION

The aim of this systematic review was to identify and evaluate interventions and programmes designed to prevent or treat obesity among children aged 6 months-5 years, implemented and evaluated in the UK. Six studies met the inclusion criteria, all of which included a combination of diet and physical activity components. Compared to baseline, three interventions reported a reduction in zBMI immediately after completing the intervention(37,38) and four interventions a while after the intervention end,(34,36,38) with the effect ranging from -0.02 to -0.9 for the preventative interventions and from -0.3 to -0.5 for the treatment ones. Three of these reductions were significant(34,37,38) (two of which targeted children with overweight/obesity).(37,38) Compared to the control conditions, five interventions showed a reduction in zBMI,(34–36,38) of which one was significant.(34)

This is the first systematic review that focuses on childhood obesity interventions among children aged 6 months-5 years evaluated in the UK. Our findings support existing evidence from international reviews that multicomponent interventions, with both dietary behaviours and physical activity elements, are effective in reducing zBMI among pre-schoolers.(26,39,40) Small effects were found at individual level; however, if the interventions are delivered at population level, the effects can be larger and meaningful.(41) The present work contributes to

For peer review only

the research by synthesizing the evidence of recent interventions and assessing their effects on children of any weight status or on children with overweight and obesity.

The current review highlights a lack of UK interventions with robust evaluation. Only Planet Munch was evaluated through a randomised controlled trial. ToyBox and HENRY were evaluated with feasibility cluster randomised trials without a follow-on full trial being published, and these feasibility trials were not adequately powered to detect zBMI changes. Methodological limitations across the studies identified, such as unclear adherence to the intervention, limit our ability to draw firm conclusions about their impact. Evaluating interventions is complex; it is important not only to ascertain impact, but also when, how, and in which conditions and for whom interventions work. A theoretical framework, a process to identify failures in implementation, an assessment of the sample, and a range of outcomes, in addition to adaptation to local settings, are important contributory factors to the effectiveness and applicability of an intervention.(42,43)

Of the studies identified in this review, only two were followed up beyond 12-months(34,38) meaning there is very little evidence for longer-term effects. All the studies included in this review were small-scale evaluations and not easily generalisable. While effect sizes give some measure of clinical importance (if adequately powered), statistical significance is affected by the sample size, and so has limited importance.(44) A reduction in zBMI may be clinically relevant when setting a child on a healthier growth trajectory and improving health outcomes.(45)

Overall, we found a lack of evaluations of interventions that recorded anthropometric outcomes. There were evaluations that reported indirect indicators of intervention impact

For peer review only - http://bmjopen.bmj.com/site/about/guidelines.xhtml

including reductions in calorie intake or increases in physical activity; however, these indicators can be prone to bias. Children and parents often underreport their energy intake,(46),(47) with greater underreporting often observed among children living with obesity.(48) Subjective measures of physical activity also often have low validity and may be biased due to poor recall or social desirability.(49) There are evaluations of interventions (e.g., the HENRY programme) that show effectiveness in changing energy balance behaviours(50) but have not been included in this review.

Within a whole-systems approach, local authorities can play a leading role in influencing health behaviour. However, it is important to recognize that financial constraints can limit local action to predominantly statutory services. Owing to these and other limitations, many local authorities have developed interventions relevant to the early years which have not been subject to robust assessment. These are often brief interventions (e.g., cooking classes or baby yoga), for which there is no evidence of meaningful and sustained lifestyle changes. Published studies also had a high risk of bias, limiting the confidence in their results. Additionally, findings were difficult to compare owing to heterogeneity in study designs, intervention approaches, and outcome reporting. Some of the reported reductions in zBMI should be interpreted with caution. For example, children that received the *HENRY* intervention started the programme with higher mean zBMI than those in the control group, which may have influenced the effect size. This highlights the need for appropriately designed RCTs with stated primary outcomes, in order to evaluate obesity interventions. Indicators of eating behaviour, physical activity and other energy balance behaviours are important,

For peer review only

anthropometric measures remain the most robust and reliable measure of intervention effectiveness.

Findings from this review suggest that interventions that promote healthy growth in the early years could be effective in reducing the risk of obesity as part of a programme of policies and interventions for young children and families. However, all studies would benefit from evaluation using large-scale RCTs before firm conclusions can be drawn. Unpublished data were provided for two trials relating to the *Planet Munch* (formerly Trim Tots) intervention, which found reductions in zBMI for children living with overweight or obesity or who were at high risk. Importantly, reductions were maintained at longer-term follow-up, up to 24 months after completing the intervention. *Planet Munch* was the only preschool obesity intervention in the UK that complied with all NICE recommendations i.e., provided advice on how to achieve a healthy diet, encouraged physical activity and included behaviour change strategies in the whole family.

The *LEAF* Programme was also effective in reducing zBMI but was only delivered to children with severe obesity and was not evaluated in an RCT. Our findings are consistent with previous work,(26) which found obesity management interventions to be more effective compared to preventative interventions in young children. This is an expected outcome, considering the different populations in treatment and prevention trials. Treatment interventions aim to slow weight gain and normalise zBMI while preventative interventions aim to maintain a healthy growth trajectory. Both aim to promote healthy lifestyle behaviours.

This systematic review has limitations. The initial search strategy included international evidence without applying limits on geographical location or language and thus yielded a high

For peer review only

number of records. We used EPPI-Reviewer software to apply an active learning approach, which greatly reduced screening time but resulted in 12,004 records being excluded without being screened. Though we also conducted a hand search, there may be relevant studies that have not been included in this review. We found but did not include a child-care self-assessment intervention that aimed to improved physical activity, oral health and nutrition for children aged 2-4 years and was delivered in nurseries.(51) The study was published but not formally peer reviewed, meaning it did not fulfil our criteria for inclusion. We also excluded studies published before 2011; however, previous systematic reviews have not included any UK studies conducted prior to that point, so it is unlikely that any robustly evaluated interventions had been conducted.

Only a small number of preschool obesity interventions have been implemented and evaluated in the UK and a smaller number have been robustly evaluated in randomised controlled trials. Meanwhile obesity prevalence and the health inequalities remain high in the UK. Obesity policies and interventions are not always implemented or delivered at scale, and implemented interventions are rarely evaluated robustly. Interventions often target individual behaviour changes without addressing the structural and systemic determinants of obesity.(52)The evaluation outcomes of some interventions are encouraging, but more evidence is needed via larger scale trials. However, scaling-up interventions can be challenging, as adaptations are often required, to meet real-world contexts. Intervention effects can also decrease when applied to the general population, so adaptations should consider the context, implementation and setting of the intervention.(50,53) More research is required to understand what support might be most useful to UK pre-schoolers and their families.

For peer review only

CONCLUSIONS

This review found that there are very few evaluations of childhood obesity interventions in preschool children in the UK. Some interventions reported effect sizes that could be clinically significant if replicated in larger sample sizes. Effect sizes were smaller in preventative interventions where the potential for zBMI change is smaller compared to children living with excess weight at the outset. However, improvements in lifestyle behaviours were reported that may reduce the risk of excess weight gain in childhood. More interventions that aim to prevent and treat obesity in preschool children are needed in the UK. Robust evaluation, ideally in RCTs will be crucial in order to guide policy makers and inform evidence-based practice in families, in communities and local authorities in the UK.

For peer review only

Acknowledgements: We thank Dr. Julie Lanigan and all the Planet Munch team that agreed to share with us findings from their unpublished work, entitled: 'The TrimTots Healthy Lifestyle Programme for prevention of obesity in preschool children: evidence from 2 randomised controlled trials'. Also, we would like to thank Dr. Steven Hope for commenting on the final draft of the manuscript. Finally, we would like to thank the Family Research Advisory Group for their contribution in our research.

Authorship confirmation: SR and RV conceived and designed the project. SM, SR and CS designed the search strategy, and SM, MS and CS conducted the searches. SM, MS and JP conducted screening, data extraction, and assessment of bias. JL provided data from one unpublished study (two trials). SM, MS and SR analysed the data. All authors contributed to the interpretation of findings. SM drafted the paper and all authors revised and edited the manuscript, before approving the final draft.

Data availability statement: All data relevant to the study are included in the article or uploaded as supplementary information.

Ethics statement: This study was exempt from the requirement of approval by the UCL Ethics Committee as it used secondary data available in the public domain and it is not possible to identify individuals from the information provided.

For peer review only

References

1. NHS Digital [Internet]. [cited 2022 Nov 15]. National Child Measurement Programme, England 2020/21 School Year. Available from: https://digital.nhs.uk/data-and-information/publications/statistical/national-child-measurement-programme/2020-21-school-year

2. NHS Digital [Internet]. [cited 2022 Nov 15]. National Child Measurement Programme, England, Provisional 2021/22 School Year Outputs. Available from: https://digital.nhs.uk/data-and-information/publications/statistical/national-child-measurement-programme/england-provisional-2021-22-school-year-outputs

3. Umer A, Kelley GA, Cottrell LE, Giacobbi P, Innes KE, Lilly CL. Childhood obesity and adult cardiovascular disease risk factors: A systematic review with meta-analysis. BMC Public Health. 2017;17(1):1–24.

4. Rankin J, Matthews L, Cobley S, Han A, Sanders R, Wiltshire HD, et al. Psychological consequences of childhood obesity: psychiatric comorbidity and prevention. Adolesc Health Med Ther. 2016;Volume 7:125–46.

5. Van Geel M, Vedder P, Tanilon J. Are overweight and obese youths more often bullied by their peers? A meta-analysis on the relation between weight status and bullying. Int J Obes. 2014;38(10):1263–7.

6. Abdelaal M, le Roux CW, Docherty NG. Morbidity and mortality associated with obesity. Ann Transl Med. 2017;5(7):1–12.

7. Simmonds M, Llewellyn A, Owen CG, Woolacott N. Predicting adult obesity from childhood obesity: A systematic review and meta-analysis. Obes Rev. 2016;17(2):95–107.

8. Mandy Geserick, M.Sc., Mandy Vogel, Ph.D., Ruth Gausche, M.B.A., Tobias Lipek, M.D., Ulrike Spielau, M.Sc., Eberhard Keller, M.D., Roland Pfäffle, M.D., Wieland Kiess, M.D., and Antje Körner MD, M V, R G, T L, U S, E K, et al. Acceleration of BMI in early childhood and risk of sustained obesity. N Engl J Med. 2018;

9. Pearce M, Webb-Phillips S, Bray I. Changes in objectively measured BMI in children aged 4-11 years: Data from the National Child Measurement Programme. J Public Health U K. 2016;38(3):459–66.

10. Anderson EL, Tilling K, Fraser A, Macdonald-Wallis C, Emmett P, Cribb V, et al. Estimating trajectories of energy intake through childhood and adolescence using linear-spline multilevel models. Epidemiology. 2013;24(4):507–15.

11. Hebestreit A, Börnhorst C, Pala V, Barba G, Eiben G, Veidebaum T, et al. Dietary energy density in young children across Europe. Int J Obes. 2014;38:S124–34.

For peer review only

12. Niederer I, Zahner L, Bürgi F, Kriemler S, Ebenegger V, Marques-Vidal P, et al. BMI group-related differences in physical fitness and physical activity in preschool-Age children: A cross-sectional analysis. Res Q Exerc Sport. 2012;83(1):12–9.

13. Public Health England. Government Dietary Recommendations. Gov Diet Recomm. 2016;1–12.

14. NHS Digital. Health Survey for England, Fruit and Vegetable Consumption, 2018. 2019.

15. O'Dwyer M V., Foweather L, Stratton G, Ridgers ND. Physical activity in non-overweight and overweight UK preschool children: Preliminary findings and methods of the Active Play Project. Sci Sports. 2011;26(6):345–9.

16. NHS England. Physical activity guidelines for children (under 5 years).

17. Ambrosini GL, Emmett PM, Northstone K, Howe LD, Tilling K, Jebb SA. Identification of a dietary pattern prospectively associated with increased adiposity during childhood and adolescence. World Rev Nutr Diet. 2014;109(2):13.

18. Jones RA, Hinkley T, Okely AD, Salmon J. Tracking physical activity and sedentary behavior in childhood: A systematic review. Am J Prev Med. 2013;44(6):651–8.

19. National Institute for Health and Care Excellence. Obesity in children and young people : prevention and lifestyle weight management programmes. (July 2015).

20. Department of Health and Social Care. Tackling obesity: empowering adults and children to live healthier lives. 2020.

21. Smith JD, Fu E, Kobayashi M. Prevention and Management of Childhood Obesity and its Psychological and Health Comorbidities. Annu Rev Clin Psychol [Internet]. 2020 May 7 [cited 2023 Dec 1];16:351–78. Available from: https://www.ncbi.nlm.nih.gov/pmc/articles/PMC7259820/

22. Ickes MJ, McMullen J, Haider T, Sharma M. Global School-Based Childhood Obesity Interventions: A Review. Int J Environ Res Public Health [Internet]. 2014 Sep [cited 2023 Dec 1];11(9):8940–61. Available from: https://www.mdpi.com/1660-4601/11/9/8940

23. Page MJ, McKenzie JE, Bossuyt PM, Boutron I, Hoffmann TC, Mulrow CD, et al. The PRISMA 2020 statement: an updated guideline for reporting systematic reviews. BMJ [Internet]. 2021 Mar 29 [cited 2023 Jun 8];372:n71. Available from: https://www.bmj.com/content/372/bmj.n71

24. Wang S, Moss JR, Hiller JE. Applicability and transferability of interventions in evidence-based public health. Health Promot Int. 2006;21(1):76–83.

25. Flynn MAT, McNeil DA, Maloff B, Mutasingwa D, Wu M, Ford C, et al. Reducing obesity and related chronic disease risk in children and youth: A synthesis of evidence with 'best practice' recommendations. Vol. 7, Obesity Reviews. 2006. 7–66 p.

For peer review only - http://bmjopen.bmj.com/site/about/guidelines.xhtml

26. Ling J, Robbins LB, Wen F. Interventions to prevent and manage overweight or obesity in preschool children: A systematic review. Int J Nurs Stud. 2016;53:270–89.

27. Institute of Education (IOE). EPPI-Reviewer Web (Beta) [Internet]. Available from: https://eppi.ioe.ac.uk/EPPIReviewer-Web/home

28. EPPI Reviewer. Priority Screening.

29. Kouwenhoven SMP, Muts J, Finken MJJ, van Goudoever JB. Low-Protein Infant Formula and Obesity Risk. Nutrients [Internet]. 2022 Jun 30 [cited 2023 Nov 30];14(13):2728. Available from: https://www.ncbi.nlm.nih.gov/pmc/articles/PMC9268498/

30. Appleton J, Russell CG, Laws R, Fowler C, Campbell K, Denney-Wilson E. Infant formula feeding practices associated with rapid weight gain: A systematic review. Matern Child Nutr [Internet]. 2018 Apr 14 [cited 2023 Nov 30];14(3):e12602. Available from: https://www.ncbi.nlm.nih.gov/pmc/articles/PMC6866175/

31. Julian, Higgins, James T. 6.3. 2022. Cochrane Handbook for Systematic Reviews of Interventions. Available from: https://training.cochrane.org/handbook/current

32. Sterne JAC, Savović J, Page MJ, Elbers RG, Blencowe NS, Boutron I, et al. RoB 2: A revised tool for assessing risk of bias in randomised trials. The BMJ. 2019;366:1–8.

33. Sterne JA, Hernán MA, Reeves BC, Savović J, Berkman ND, Viswanathan M, et al. ROBINS-I: A tool for assessing risk of bias in non-randomised studies of interventions. BMJ Online. 2016;355:4–10.

34. Hodgkinson A, Abbott J, Hurley MA, Lowe N, Qualter P. An educational intervention to prevent overweight in pre-school years: a cluster randomised trial with a focus on disadvantaged families. BMC Public Health [Internet]. 2019 Nov 1 [cited 2022 Nov 24];19(1):1430. Available from: https://doi.org/10.1186/s12889-019-7595-2

35. Malden S, Reilly JJ, Gibson AM, Bardid F, Summerbell C, De Craemer M, et al. A feasibility cluster randomised controlled trial of a preschool obesity prevention intervention: ToyBox-Scotland. Pilot Feasibility Stud. 2019;5(1):1–11.

36. Bryant M, Collinson M, Burton W, Stamp E, Schofield H, Copsey B, et al. Cluster randomised controlled feasibility study of HENRY: a community-based intervention aimed at reducing obesity rates in preschool children. Pilot Feasibility Stud. 2021;7(1):59.

37. Tee LP, Brandreth RA, Sauven N, Clarke L, Frampton I. Successful outcomes in childrens specialist weight management: Impact assessment of a novel early years weight management programme. J Hum Nutr Diet. 2021;34(5):819–26.

38. Julie Lanigan, Sarah Collins, Kerry Coyte, Toni Birbara, Maria Kokoreli, Tim Cole, et al. The TrimTots Healthy Lifestyle Programme for prevention of obesity in preschool children: evidence from 2 randomised controlled trials. Unpublished.

For peer review only - http://bmjopen.bmj.com/site/about/guidelines.xhtml

39. Brown T, Moore TH, Hooper L, Gao Y, Zayegh A, Ijaz S, et al. Interventions for preventing obesity in children. Cochrane Database Syst Rev. 2019;2019(7).

40. Colquitt JL, Loveman E, O'Malley C, Azevedo LB, Mead E, Al-Khudairy L, Ells LJ, Metzendorf MI RK. Diet, physical activity, and behavioural interventions for the treatment of overweight or obesity in preschool children up to the age of 6 years (Review). Cochrane Database Syst Rev. 2016;

41. Matthay EC, Hagan E, Gottlieb LM, Tan ML, Vlahov D, Adler N, et al. Powering population health research: Considerations for plausible and actionable effect sizes. SSM - Popul Health. 2021;14:100789.

42. Mills A, Gilson L, Hanson K, Palmer N, Lagarde M. What do we mean by rigorous health-systems research? The Lancet. 2008;372(9649):1527–9.

43. Craig P, Dieppe P, Macintyre S, Mitchie S, Nazareth I, Petticrew M. Developing and evaluating complex interventions: The new Medical Research Council guidance. Bmj. 2008;337(7676):979–83.

44. Dunkler D, Haller M, Oberbauer R, Heinze G. To test or to estimate? P-values versus effect sizes. Transpl Int. 2020;33(1):50–5.

45. Ryan DH, Yockey SR. Weight Loss and Improvement in Comorbidity: Differences at 5%, 10%, 15%, and Over. Curr Obes Rep. 2017;6(2):187–94.

46. Livingstone MBE, Robson PJ, Wallace JMW. Issues in dietary intake assessment of children and adolescents. Br J Nutr. 2004;92(S2):S213–22.

47. Foster E, Hawkins A, Barton KL, Stamp E, Matthews JNS, Adamson AJ. Development of food photographs for use with children aged 18 months to 16 years: Comparison against weighed food diaries-The Young Person's Food Atlas (UK). PLoS ONE. 2017;12(2):1–18.

48. Swinburn B, Sacks G, Ravussin E. Increased food energy supply is more than sufficient to explain the US epidemic of obesity. Am J Clin Nutr. 2009;90(6):1453–6.

49. Strath SJ, Kaminsky LA, Ainsworth BE, Ekelund U, Freedson PS, Gary RA, et al. Guide to the assessment of physical activity: Clinical and research applications: A scientific statement from the American Heart association. Circulation. 2013;128(20):2259–79.

50. Pfadenhauer LM, Gerhardus A, Mozygemba K, Lysdahl KB, Booth A, Hofmann B, et al. Making sense of complexity in context and implementation: The Context and Implementation of Complex Interventions (CICI) framework. Implement Sci. 2017;12(1):1–17.

51. Kipping R, Langford R, Brockman R, Wells S, Metcalfe C, Papadaki A, et al. Child-care self-assessment to improve physical activity, oral health and nutrition for 2- to 4-year-olds: a feasibility cluster RCT. Public Health Res [Internet]. 2019 Jul [cited 2023 Nov 30];7(13):1–164. Available from: https://www.journalslibrary.nihr.ac.uk/phr/phr07130

For peer review only - http://bmjopen.bmj.com/site/about/guidelines.xhtml

52. THEIS DRZ, WHITE M. Is Obesity Policy in England Fit for Purpose? Analysis of Government Strategies and Policies, 1992–2020. Milbank Q [Internet]. 2021 Mar [cited 2023 Nov 27];99(1):126–70. Available from: https://www.ncbi.nlm.nih.gov/pmc/articles/PMC7984668/

53. McCrabb S, Lane C, Hall A, Milat A, Bauman A, Sutherland R, et al. Scaling-up evidence-based obesity interventions: A systematic review assessing intervention adaptations and effectiveness and quantifying the scale-up penalty. Obes Rev. 2019;(December 2018):964–82.

For peer review only

Tables included in the text

Table 1 Characteristics of the included studies

Author, year, study type	Participants eligible / targeted	Participants randomized (intervention, control)[a]	Intervention duration	Time of the follow-up measurements	Participants with valid data in the measurements	Change in mean zBMI 1st or 2nd measurement vs baseline (SD / CI 95%)	Change in mean zBMI vs control (SD / CI 95%)	When evaluated
Hodgkinson et al., (2019), Pragmatic CRT	2-4 years, Mean age=NR	87 (48, 39)	6 months	2-years post baseline	81 (47, 34)	-0.18* (SD 0.99)	-0.74* (-1.10 to -0.38)	NR (Trial published in 2019)
Malden et al., (2019), Feasibility CRT	3-5 years, Mean age=4.4	42 (26, 16)	18 weeks	15th-17th week of the intervention	36 (22, 14)	+0.02 (-0.11 to 0.15)	-0.04 (SD 0.13)	2018
Bryant et al., (2021), Feasibility CRT	6 months - 5 years Mean age=NR	117 (47, 70)	8 weeks	11-months post baseline	99 (39, 60)	-0.9 (SD 0.46)	-1.4 (SD 0.08)	NR (Trial published in 2021)
Lanigan et al., (Unpublished) RCT – trial 1	1-5 years, with OW/OB/high risk Mean age=2.4	88 (49, 39)	6 months	6-months post baseline	64 (36, 28)	-0.2 (-0.3 to -0.03)	-0.2 (-0.5 to 0.05)	(Trial not yet published)
				24 months after the end of the intervention	42	-0.3 * (-0.5, -0.1),	NR	(Trial not yet published)
Lanigan et al., (Unpublished) RCT -trial 2	1-5 years, of any weight status. Mean age=2.1	85 (38, 47)	6 months	6-months post baseline	64 (27, 37)	-0.02 (-0.2 to 0.2),	-0.09 (-0.4 to 0.2)	(Trial not yet published)
				12 months after the end of the intervention	39	-0.3 (-0.7, 0.1),	NR	(Trial not yet published)

For peer review only - http://bmjopen.bmj.com/site/about/guidelines.xhtml

Tee et al., (2021), Impact Assessment	Children aged ≤6 years with severe OB Mean age=4.1	147	12 months	12 months post baseline	41	-0.5*	NR	2012-17

CRT, cluster randomised trial, NR, not reported, NA, not applicable, RCT, randomised controlled trial, OW, overweight, OB, obesity

[a]: Numbers at randomisation, *: significant change in zBMI

For peer review only

For peer review only - http://bmjopen.bmj.com/site/about/guidelines.xhtml

Figure legends

Figure 1 Flowchart of the included studies

For peer review only

For peer review only - http://bmjopen.bmj.com/site/about/guidelines.xhtml

For peer review only

For peer review only - http://bmjopen.bmj.com/site/about/guidelines.xhtml

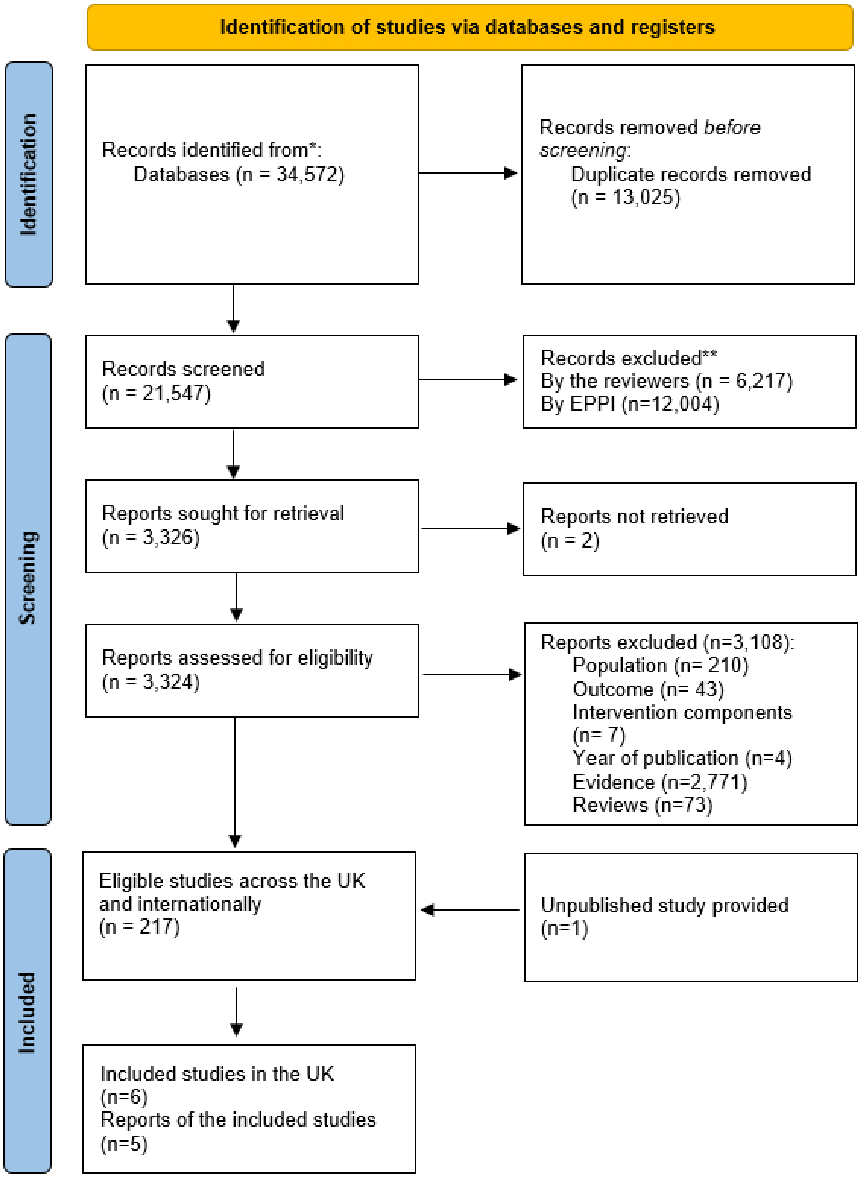

Figure 1 Flowchart of the included studies

408x528mm (38 x 38 DPI)

Supplementary Material

Appendix 1 – Search terms and search strategy

The following databases were searched in November 2021. The searches were updated in March 2023.

1. Pubmed/Medline
2. Cochrane Library Web
3. CINAHL
4. Scopus
5. PsycINFO
6. Web of Science
7. Social Policy and Practice
8. Health Management Information Consortium (HMIC)
9. Trials Register of Promoting Health Interventions (TRoPHI)

The indicative search terms that were used are presented on the table below:

Table S1: Search terms

Concept 1<br><br>Participants	Concept 2<br><br>Exposure	Concept 3<br><br>Outcomes
Subject headings:<br><br>"Child, preschool" OR<br><br>"Schools, nursery" OR<br><br>"Child day care centers"	(("diet" OR "food" OR "beverages" OR "food quality" OR "food preferences" OR "feeding behavior*")<br><br>OR<br><br>("exercise" OR "sedentary behavior" " OR "physical fitness"))<br><br>AND<br><br>("weight reduction programs" OR "health education" OR "health promotion" OR "primary prevention" OR "pilot projects" OR "feasibility studies" OR "program evaluation")	("obesity" OR "body mass index" OR "body weight" OR "body size" OR "body composition" OR "body weight changes" OR

For peer review only

Keywords<br><br>"child preschool" OR "preschool*" OR "preschool*" OR "pre school*" OR "kindergarten" OR "kindergarden" OR "prekinder*" OR "pre kinder*" OR "pre kinder*" OR "headstart" OR "head start" OR "sure start" OR "surestart" OR "family child care home*" OR "family childcare home*" OR "infant*" OR "toddler*" OR "childcare" OR "child-care" OR "child-care" OR "early learning center*" OR "early learning centre*" OR "early child*" OR "early care and education" OR ("child*" AND ("day-care" OR "day-care" OR "daycare")) OR "playgroup*" OR "play group*" OR "schools nursery" OR (("center" OR "center s" OR "centers"OR "centre" OR "centre s" OR "centres") AND "child, preschool") OR "child day care centers" OR "child day care centers")	(("diet" OR "dietary" OR "food" OR "beverages"OR "snack*" OR "fruit*" OR "vegetable*" OR OR "food quality" OR "drink*" OR "juice*" OR "nutrition*" OR "food habit*" OR "food choice*" OR "overeat*" OR "over eat*" OR "weight control" OR "weight management" OR "menu planning" OR "menu choice*" OR "meal choice*" OR "meal*" OR "meal planning" OR ("food" AND "consum*") OR "food preferences" OR "feeding behavior*" OR "feeding behaviour*" OR "energy density" OR "lifestyle behaviour" OR "eating behaviour" OR "healthy eating" OR "health promotion" OR "eating habit*")<br><br>OR<br><br>("exercise" OR "sedentary behaviour OR "physical fitness" OR "movement" OR "playtime"OR "play*" OR "sport*"))<br><br>AND<br><br>("intervention*" OR "program*" OR "programme*" OR "project*" OR "therapeutics" OR "scheme" OR "initiative*" OR "strateg*" OR "service*" OR "primary prevention" OR "health education" OR "health promotion" OR "primary prevention" OR "treat*" OR "therapy*" OR "pilot projects" OR "feasibility studies" OR "program evaluation")	"overweight")("obesity" OR "obes*" OR "body mass index" OR "BMI" OR "bmi z score" OR "bmi z score" OR "body mass index z-score" OR "body mass index z-score" OR "bmi percentile*" OR "bmi centile*" OR "body mass index percentile*" OR "body mass index centile*" OR "adipos*" OR "overweight" OR "healthy weight" OR "weight loss" OR "weight gain")

For peer review only

Search strategy in all databases:

PubMed

#1<br><br>Child/setting terms	("child, preschool"[MeSH Terms] OR "child preschool"[Title/Abstract] OR "preschool*"[Title/Abstract] OR "preschool*"[Title/Abstract] OR "pre school*"[Title/Abstract] OR "kindergarten"[Title/Abstract] OR "kindergarden"[Title/Abstract] OR "prekinder*"[Title/Abstract] OR "pre kinder*"[Title/Abstract] OR "pre kinder*"[Title/Abstract] OR "headstart"[Title/Abstract] OR "head start"[Title/Abstract] OR "sure start"[Title/Abstract] OR "surestart"[Title/Abstract] OR "family child care home*"[Title/Abstract] OR "family childcare home*"[Title/Abstract] OR "infant*"[Title/Abstract] OR "toddler*"[Title/Abstract] OR "childcare"[Title/Abstract] OR "child-care"[Title/Abstract] OR "child-care"[Title/Abstract] OR "early learning center*"[Title/Abstract] OR "early learning centre*"[Title/Abstract] OR "early child*"[Title/Abstract] OR "early care and education"[Title/Abstract] OR ("child*"[Title/Abstract] AND ("day-care"[Title/Abstract] OR "day-care"[Title/Abstract] OR "daycare"[Title/Abstract])) OR "playgroup*"[Title/Abstract] OR "play group*"[Title/Abstract] OR "schools, nursery"[MeSH Terms] OR "schools nursery"[Title/Abstract] OR (("center"[Title/Abstract] OR "center s"[Title/Abstract] OR "centers"[Title/Abstract] OR "centre"[Title/Abstract] OR "centre s"[Title/Abstract] OR "centres"[Title/Abstract]) AND "child, preschool"[MeSH Terms]) OR "child day care centers"[MeSH Terms] OR "child day care centers"[Title/Abstract])
#2<br><br>Diet terms	("diet"[MeSH Terms] OR "diet"[Title/Abstract] OR "dietary"[Title/Abstract] OR "food"[MeSH Terms] OR "food"[Title/Abstract] OR "beverages"[MeSH Terms] OR "beverages"[Title/Abstract] OR "snack*"[Title/Abstract] OR "fruit*"[Title/Abstract] OR "vegetable*"[Title/Abstract] OR "food quality"[MeSH Terms] OR "food quality"[Title/Abstract] OR "drink*"[Title/Abstract] OR "juice*"[Title/Abstract] OR "nutrition*"[Title/Abstract] OR "food habit*"[Title/Abstract] OR "food choice*"[Title/Abstract] OR "overeat*"[Title/Abstract] OR "over eat*"[Title/Abstract] OR "weight control"[Title/Abstract] OR "weight management"[Title/Abstract] OR "menu planning"[Title/Abstract] OR "menu choice*"[Title/Abstract] OR "meal choice*"[Title/Abstract] OR "meal*"[Title/Abstract] OR "meal planning"[Title/Abstract] OR ("food"[Title/Abstract] AND "consum*"[Title/Abstract]) OR "food preferences"[MeSH Terms] OR "food preferences"[Title/Abstract] OR "feeding behavior*"[MeSH Terms] OR "feeding behavior*"[Title/Abstract] OR "feeding behaviour*"[Title/Abstract] OR "energy density"[Title/Abstract] OR "lifestyle behaviour"[Title/Abstract] OR "eating behaviour"[Title/Abstract] OR "healthy eating"[Title/Abstract] OR "health promotion"[Title/Abstract] OR "eating habit*"[Title/Abstract])
#3<br><br>**PA terms**	("exercise"[MeSH Terms] OR "exercise"[Title/Abstract] OR "sedentary behavior"[MeSH Terms] OR "sedentary behavior"[Title/Abstract] OR "sedentary behaviour"[Title/Abstract] OR "physical fitness"[MeSH Terms] OR "physical fitness"[Title/Abstract] OR "movement"[Title/Abstract] OR "playtime"[Title/Abstract] OR "play*"[Title/Abstract] OR "sport*"[Title/Abstract])
#4<br><br>Intervention terms	("intervention*"[Title/Abstract] OR "weight reduction programs"[MeSH Terms] OR "program*"[Title/Abstract] OR "programme*"[Title/Abstract] OR "project*"[Title/Abstract] OR "therapeutics"[Title/Abstract] OR "scheme"[Title/Abstract] OR "initiatives"[Title/Abstract] OR "strateg*"[Title/Abstract] OR "service*"[Title/Abstract] OR "pilot projects"[MeSH Terms] OR "pilot projects"[Title/Abstract] OR "feasibility studies"[MeSH Terms] OR "feasibility studies"[Title/Abstract] OR "program evaluation"[MeSH Terms] OR "program evaluation"[Title/Abstract] OR "health education"[MeSH Terms] OR "health education"[Title/Abstract] OR "health promotion"[MeSH Terms] OR "health promotion"[Title/Abstract] OR "primary prevention"[Title/Abstract] OR "treat*"[Title/Abstract] OR "random*"[Title/Abstract] OR "cluster random*"[Title/Abstract] OR "trial*"[Title/Abstract] OR "evaluat*"[Title/Abstract] OR "effective*"[Title/Abstract] OR "mixed methods"[Title/Abstract] OR "impact*"[Title/Abstract])
#5	("intervention*"[Title/Abstract] OR "weight reduction programs"[MeSH Terms] OR "program*"[Title/Abstract] OR "programme*"[Title/Abstract] OR "project*"[Title/Abstract] OR "therapeutics"[Title/Abstract] OR "scheme"[Title/Abstract] OR "initiative*"[Title/Abstract] OR "strateg*"[Title/Abstract] OR "service*"[Title/Abstract] OR "health education"[MeSH Terms] OR "health promotion"[MeSH Terms] OR "primary prevention"[MeSH Terms] OR "primary prevention"[Title/Abstract] OR "health education"[Title/Abstract] OR "health

For peer review only

	promotion"[Title/Abstract] OR "primary prevention"[Title/Abstract] OR "treat*"[Title/Abstract] OR "therapy*"[Title/Abstract] OR "pilot projects"[MeSH Terms] OR "pilot projects"[Title/Abstract] OR "feasibility studies"[MeSH Terms] OR "feasibility studies"[Title/Abstract] OR "program evaluation"[MeSH Terms] OR "program evaluation"[Title/Abstract])
#6	("random*"[Title/Abstract] OR "cluster random*"[Title/Abstract] OR "trial*"[Title/Abstract] OR "evaluat*"[Title/Abstract] OR "effective*"[Title/Abstract] OR "mixed methods"[Title/Abstract] OR "impact*"[Title/Abstract] OR "non randomized controlled trial*"[Title/Abstract] OR "Pragmatic Clinical Trial"[Title/Abstract] OR "controlled clinical trial*"[Title/Abstract] OR "randomized controlled trial*"[Title/Abstract] OR "randomized"[Title/Abstract] OR "randomised"[Title/Abstract] OR "clinical trial*"[MeSH Terms] OR "clinical trial*"[Title/Abstract] OR "randomly"[Title/Abstract] OR "controlled study"[Title/Abstract] OR "controlled trial*"[Title/Abstract] OR "control group"[Title/Abstract] OR "intervention group"[Title/Abstract] OR "experimental group"[Title/Abstract] OR "comparison group"[Title/Abstract] OR "control school"[Title/Abstract] OR "intervention school"[Title/Abstract] OR "control community"[Title/Abstract] OR "intervention community"[Title/Abstract] OR "intervention condition"[Title/Abstract] OR "control condition"[Title/Abstract] OR "treatment group"[Title/Abstract] OR "control participant"[Title/Abstract] OR "quasi experimental design"[Title/Abstract] OR "experimental condition"[Title/Abstract] OR "treatment condition"[Title/Abstract] OR "pilot projects"[MeSH Terms] OR "feasibility studies"[MeSH Terms] OR "program evaluation"[MeSH Terms] OR "pre test"[Title/Abstract] OR "post test"[Title/Abstract] OR "quasi experiment*"[Title/Abstract] OR "blinded"[Title/Abstract] OR "double bind"[Title/Abstract] OR "fidelity"[Title/Abstract] OR "reach"[Title/Abstract] OR "uptake"[Title/Abstract] OR "implementation"[Title/Abstract] OR "intervention effect*"[Title/Abstract] OR "program effect*"[Title/Abstract] OR "programme effect*"[Title/Abstract] OR "proof of concept*"[Title/Abstract] OR "outcome study"[Title/Abstract])
#7	Search: #5 AND #6
#8	Search: #2 OR #3
#9	Search: #8 AND (#4 OR #7)
**#10**	("obesity"[MeSH Terms] OR "obesity"[Title/Abstract] OR "obes*"[Title/Abstract] OR "body mass index"[MeSH Terms] OR "body mass index"[Title/Abstract] OR "BMI"[Title/Abstract] OR "bmi z score"[Title/Abstract] OR "bmi z score"[Title/Abstract] OR "body mass index z-score"[Title/Abstract] OR "body mass index z-score"[Title/Abstract] OR "bmi percentile*"[Title/Abstract] OR "bmi centile*"[Title/Abstract] OR "body mass index percentile*"[Title/Abstract] OR "body mass index centile*"[Title/Abstract] OR "body weight"[MeSH Terms] OR "body size"[MeSH Terms] OR "adipos*"[Title/Abstract] OR "body composition"[MeSH Terms] OR "body weight changes"[MeSH Terms] OR "overweight"[MeSH Terms] OR "overweight"[Title/Abstract] OR "healthy weight"[Title/Abstract] OR "weight loss"[Title/Abstract] OR "weight gain"[Title/Abstract])
**#11**	Search: #1 AND #9 AND #10

For peer review only

CINAHL

#1 Population AB	AB (preschool* OR pre-school* OR “pre-school*” OR kindergar?en OR prekinder* OR pre-Kinder* OR “pre Kinder*” OR headstart OR “head start” OR “surestart” OR “sure start” OR “family child care home” OR “family childcare home” OR “infant*” OR “toddler*” OR “child care” OR “child-care” OR childcare OR “early learning centre” OR “early child*” OR “early care and education” OR (child* AND “day care” OR “daycare” OR “day-care” OR playgroup* OR “nurser*” OR child day care centre*)) **OR** TI ((preschool* OR pre-school* OR “pre-school*” OR kindergar?en OR prekinder* OR pre-Kinder* OR “pre Kinder*” OR headstart OR “head start” OR “surestart” OR “sure start” OR “family child care home” OR “family childcare home” OR “infant*” OR “toddler*” OR “child care” OR “child-care” OR childcare OR “early learning centre” OR “early child*” OR “early care and education” OR (child* AND “day care” OR “daycare” OR “day-care” OR playgroup* OR “nurser*” OR child day care centre*))
#2 Diet AB	AB (diet* OR dietary OR food* OR snack* OR fruit* OR drink* OR beverage* OR juice* OR vegetable* OR meal* OR nutrition* OR intake* OR consum* OR “food quality” OR “food preference*” OR “energy density” OR “lifestyle behavio#r” OR “eating behavio#r” OR “healthy eating” OR “feeding behavio#r” OR “health promotion” OR “eating habit*” OR “food habit*” OR “food choice*” OR “overeat*” OR “over eat*” OR “weight control” OR “weight management” OR “menu planning” OR “menu choice” OR “meal choice*” OR “meal*” OR “meal planning” OR (food* AND consum*) OR “eating habit*”) **OR** TI (diet* OR dietary OR food* OR snack* OR fruit* OR drink* OR beverage* OR juice* OR vegetable* OR meal* OR nutrition* OR intake* OR consum* OR “food quality” OR “food preference*” OR “energy density” OR “lifestyle behavio#r” OR “eating behavio#r” OR “healthy eating” OR “feeding behavio#r” OR “health promotion” OR “eating habit*” OR “food habit*” OR “food choice*” OR “overeat*” OR “over eat*” OR “weight control” OR “weight management” OR “menu planning” OR “menu choice” OR “meal choice*” OR “meal*” OR “meal planning” OR (food* AND consum*) OR “eating habit*”)
#3 PA AB	AB (“physical* activ*” OR playtime OR sedentary OR exercise* OR play* OR “physical fitness” OR “movement” OR “playtime” OR “sport*”) **OR** TI (“physical* activ*” OR playtime OR sedentary OR exercise* OR play* OR “physical fitness” OR “movement” OR “playtime” OR “sport*”)
#4 Interventions AB	AB (“intervention*” OR “weight reduction program*” "program*" OR "programme*" OR "project*" OR "therapeutics" OR "scheme" OR "initiative*" OR "strateg*" OR "life style" OR "service*" OR “pilot project*” OR “feasibility stud*” OR “program evaluation” OR “health education*” OR “health promotion” OR “primary prevention” OR “treat*” OR “random*” OR “cluster random*” OR “trial*” OR “evaluat*” OR “effective*” OR “mixed methods” OR “impact*”) **OR** TI (“intervention*” OR “weight reduction program*” "program*" OR "programme*" OR "project*" OR "therapeutics" OR "scheme" OR "initiative*" OR "strateg*" OR "life style" OR "service*" OR “pilot project*” OR “feasibility stud*” OR “program evaluation” OR “health education*” OR “health promotion” OR “primary prevention” OR “treat*” OR “random*” OR “cluster random*” OR “trial*” OR “evaluat*” OR “effective*” OR “mixed methods” OR “impact*”)
#5 Intervention terms	AB ("intervention*" OR "weight reduction programs" OR "program*" OR "programme*" OR "project*" OR "therapeutics" OR "scheme" OR "initiative*" OR "strateg*" OR "service*" OR "health education" OR "health promotion" OR "primary prevention" OR "health education" OR "health promotion" OR "primary prevention" or "treat*" OR therapy* or "pilot projects" OR "feasibility studies" OR "program evaluation") **OR** TI ("intervention*" OR "weight reduction programs" OR "program*" OR "programme*" OR "project*" OR "therapeutics" OR "scheme" OR "initiative*" OR "strateg*" OR "service*" OR "health education" OR "health promotion" OR "primary prevention" OR "health education" OR "health promotion" OR "primary prevention" or "treat*" OR therapy* or "pilot projects" OR "feasibility studies" OR "program evaluation")
#6 Evaluati	AB ("random*" OR "cluster random*" OR "trial*" OR "evaluat*" OR "effective*" OR "mixed methods" OR "impact*" OR "Non-Randomized Controlled Trial*" OR "Pragmatic Clinical Trial" OR "controlled Clinical Trial*" OR "randomized controlled trial*" OR “randomized” OR “randomised” OR "clinical trial*" OR “randomly” OR "controlled study" OR "controlled trial*" OR "control group" OR "intervention group" OR "experimental group" OR "comparison group" OR "control school" OR "intervention school" OR "control community" OR "intervention community" OR "intervention condition" OR "control condition" OR "treatment group" OR "control participant" OR "quasi experimental design" OR

For peer review only

on ter ms	"experimental condition" OR "treatment condition" OR "pilot projects" OR "feasibility studies" OR "program evaluation" OR "pre test" OR "post test" OR "quasi experiment*" OR blinded OR "double bind" OR fidelity OR reach OR uptake OR implementation OR "intervention effect*" OR "program effect*" OR "programme effect*" OR "proof of concept*" OR "outcome study") **OR** TI ("random*" OR "cluster random*" OR "trial*" OR "evaluat*" OR "effective*" OR "mixed methods" OR "impact*" OR "Non-Randomized Controlled Trial*" OR "Pragmatic Clinical Trial" OR "controlled Clinical Trial*" OR "randomized controlled trial*" OR “randomized” OR “randomised” OR "clinical trial*" OR “randomly” OR "controlled study" OR "controlled trial*" OR "control group" OR "intervention group" OR "experimental group" OR "comparison group" OR "control school" OR "intervention school" OR "control community" OR "intervention community" OR "intervention condition" OR "control condition" OR "treatment group" OR "control participant" OR "quasi experimental design" OR "experimental condition" OR "treatment condition" OR "pilot projects" OR "feasibility studies" OR "program evaluation" OR "pre test" OR "post test" OR "quasi experiment*" OR blinded OR "double bind" OR fidelity OR reach OR uptake OR implementation OR "intervention effect*" OR "program effect*" OR "programme effect*" OR "proof of concept*" OR "outcome study")
#7	S5 AND S6
#8	S2 OR S3
#9	S4 OR S7
#10	S8 AND S9<br><br>**Limiters** - Age Groups: Infant, 1-23 months, Child, Preschool 2-5 years
#11	AB ("obesity” OR “obes*” OR "body mass index" OR "BMI" OR "bmi z score" OR "bmi z score" OR "body mass index z-score" OR "body mass index z-score" OR "bmi percentile*" OR "bmi centile*" OR "body mass index percentile*" OR "body mass index centile*" OR "body weight" OR "body size" OR "weight*" OR "waist" OR "adipos*" OR "body composition" OR "overweight" OR “body weight changes” OR “healthy weight” OR “weight loss” OR “weight gain”) **OR** TI ("obesity” OR “obes*” OR "body mass index" OR "BMI" OR "bmi z score" OR "bmi z score" OR "body mass index z-score" OR "body mass index z-score" OR "bmi percentile*" OR "bmi centile*" OR "body mass index percentile*" OR "body mass index centile*" OR "body weight" OR "body size" OR "weight*" OR "waist" OR "adipos*" OR "body composition" OR "overweight" OR “body weight changes” OR “healthy weight” OR “weight loss” OR “weight gain”)<br><br>**Limiters** - Age Groups: Infant, 1-23 months, Child, Preschool 2-5 years
#12	#1 AND #10 AND #11<br><br>**Limiters** - Publication Year: 2011-2023

For peer review only

Cochrane

#1<br><br>Population<br><br>TI-AB-KW	(preschool* OR pre-school* OR “pre-school*” OR kindergar?en OR prekinder* OR pre-Kinder* OR “pre Kinder*” OR headstart OR “head start” OR “surestart” OR “sure start” OR “family child care home” OR “family childcare home” OR “infant*” OR “toddler*” OR “child care” OR “child-care” OR childcare OR “early learning centre” OR “early child*” OR “early care and education” OR (child* AND “day care” OR “daycare” OR “day-care” OR playgroup* OR “nurser*” OR child day care centre*))
#2<br><br>Diet<br><br>TI-AB-KW	(diet* OR dietary OR food* OR snack* OR fruit* OR drink* OR beverage* OR juice* OR vegetable* OR meal* OR nutrition* OR intake* OR consum* OR "food quality" OR "food preference*" OR "energy density" OR "lifestyle behavio*r" OR "eating behavio*r" OR "healthy eating" OR "feeding behavio*r" OR "health promotion" OR "eating habit*" OR "food habit*" OR "food choice*" OR "overeat*" OR "over eat*" OR "weight control" OR "weight management" OR "menu planning" OR "menu choice*" OR "meal choice*" OR "meal*" OR "meal planning" OR (food* AND consum*) OR "eating habit*")
#3<br><br>PA<br><br>TI-AB-KW	(“physical* activ*” OR sport OR sports OR exercis* OR playtime OR sedentary OR exercise* OR play* OR “physical fitness” OR “movement” OR “playtime”)
#4<br><br>Outcome terms<br><br>TI-AB-KW	(obesity OR obes* OR "body mass index" OR BMI OR "bmi z score" OR "bmi z score" OR "body mass index z-score" OR "body mass index z-score" OR "bmi percentile*" OR "bmi centile*" OR "body mass index percentile*" OR "body mass index centile*" OR "body weight" OR "body size" OR weight* OR waist OR "adipos*" OR "body composition" OR overweight OR “body weight changes” OR “healthy weight” OR “weight loss” OR “weight gain”)
#5	#1 AND #6 AND #7
#6	("intervention*" OR "weight reduction programs" OR "program*" OR "programme*" OR "project*" OR "therapeutics" OR "scheme" OR "initiative*" OR "strateg*" OR "service*" OR "health education" OR "health promotion" OR "primary prevention" OR "health education" OR "health promotion" OR "primary prevention" or "treat*" OR therapy* or "pilot projects" OR "feasibility studies" OR "program evaluation")
#7	#5 AND #6

Web of Science

#1 Population TS	TS=(preschool* OR pre-school* OR “pre-school*” OR kindergar$en OR prekinder* OR pre-Kinder* OR “pre Kinder*” OR headstart OR “head start” OR “surestart” OR “sure start” OR “family child care home” OR “family childcare home” OR “infant*” OR “toddler*” OR “child care” OR “child-care” OR childcare OR “early learning centre” OR “early child*” OR “early care and education” OR (child* AND “day care” OR “daycare” OR “day-care” OR playgroup* OR “nurser*” OR “child day care centre*”))
#2 Diet TS	TS=((diet* OR dietary OR food* OR snack* OR fruit* OR drink* OR beverage* OR juice* OR vegetable* OR meal* OR nutrition* OR intake* OR consum* OR “food quality” OR “food preference*” OR “energy density” OR “lifestyle behavio$r” OR “eating behavio$r” OR “healthy eating” OR “feeding behavio$r” OR “health promotion” OR “eating habit*” OR “food habit*” OR “food choice*” OR “overeat*” OR “over eat*” OR “weight control” OR “menu planning” OR “menu choice*” OR “meal choice*” OR “meal*” OR “meal planning” OR (food* AND consum*) OR “eating habit*”))
#3 PA TS	TS=((“physical* activ*” OR playtime OR sedentary OR exercise* OR play* OR “physical fitness” OR “movement” OR “playtime” OR sport*))
#4 Interventions TS	TS=((“intervention*” OR “weight reduction program*” "program*" OR "programme*" OR "project*" OR "therapeutics" OR "scheme" OR "initiative*" OR "strateg*" OR "life style" OR "service*" OR “pilot project*” OR “feasibility stud*” OR “program evaluation” OR “health education*” OR “health promotion” OR “primary prevention” OR “treat*” OR “random*” OR “cluster random*” OR “trial*” OR “evaluat*” OR “effective*” OR “mixed methods” OR “impact*”))
#5	TS=(("intervention*" OR "weight reduction programs" OR "program*" OR "programme*" OR "project*" OR "therapeutics" OR "scheme" OR "initiative*" OR "strateg*" OR "service*" OR "health education" OR "health promotion" OR "primary prevention" OR "health education" OR "health promotion" OR "primary prevention" or "treat*" OR therapy* or "pilot projects" OR "feasibility studies" OR "program evaluation"))
#6	TS=(("random*" OR "cluster random*" OR "trial*" OR "evaluat*" OR "effective*" OR "mixed methods" OR "impact*" OR "Non-Randomized Controlled Trial*" OR "Pragmatic Clinical Trial" OR "controlled Clinical Trial*" OR "randomized controlled trial*" OR “randomized” OR “randomised” OR "clinical trial*" OR “randomly” OR "controlled study" OR "controlled trial*" OR "control group" OR "intervention group" OR "experimental group" OR "comparison group" OR "control school" OR "intervention school" OR "control community" OR "intervention community" OR "intervention condition" OR "control condition" OR "treatment group" OR "control participant" OR "quasi experimental design" OR "experimental condition" OR "treatment condition" OR "pilot projects" OR "feasibility studies" OR "program evaluation" OR "pre test" OR "post test" OR "quasi experiment*" OR blinded OR "double bind" OR fidelity OR reach OR uptake OR implementation OR "intervention effect*" OR "program effect*" OR "programme effect*" OR "proof of concept*" OR "outcome study"))
#7	#5 AND #6
#8	#2 OR #3
#9	#4 OR #7
#10	#8 AND #9

#11	TS=(("obesity" OR "obes*" OR "body mass index" OR "BMI" OR "bmi z score" OR "bmi z score" OR "body mass index z-score" OR "body mass index z-score" OR "bmi percentile*" OR "bmi centile*" OR "body mass index percentile*" OR "body mass index centile*" OR "body weight" OR "body size" OR "weight*" OR "waist" OR "adipos*" OR "body composition" OR "overweight" OR "body weight changes" OR "healthy weight" OR "weight loss" OR "weight gain"))
#12	#1 AND #10 AND #11<br>and 2023 and 2022<br>and 2021 or 2020 or 2019 or 2018 or 2017 or 2016 or 2015 or 2014 or 2013 or 2012 or 2011 (Publication Years)

For peer review only

SCOPUS

1	(TITLE-ABS ((preschool* OR pre-school* OR {pre-school*} OR kindergar?en OR prekinder* OR pre kinder* OR {pre Kinder} OR headstart OR {head start} OR surestart OR {sure start} OR {family child care home} OR {family childcare home} OR infant* OR toddler* OR {child care} OR {child-care} OR childcare OR {early learning centre} OR {early child} OR {early care and education} OR (child* AND {day care} OR daycare OR {day-care}) OR playgroup* OR nurser* OR {child day care centre*}))) AND (((TITLE-ABS ((diet* OR dietary OR food* OR snack* OR fruit* OR drink* OR beverage* OR juice* OR vegetable* OR m eal* OR nutrition* OR intake* OR consum* OR {food quality} OR {food preference} OR {energy density} OR {lifestyle behavio?r} OR {eating behavio?r} OR {healthy eating} OR {feeding behavio?r} OR {health promotion} OR {eating habit*} OR {food habit*} OR {food choice*} OR {overeat*} OR {over eat*} OR {weight control} OR {menu planning} OR {menu choice} OR {meal choice} OR {meal*} OR {meal planning} OR {eating habit*}))) OR (TITLE-ABS (({physical* activ*} OR playtime OR sedentary OR exercise* OR play* OR {physical fitness} OR movement OR playtime OR sport*)))) AND ((TITLE-ABS (intervention* OR {weight reduction program*} OR program* OR programme* OR project* OR therapeutics OR scheme OR initiative OR strateg* OR lifestyle OR service* OR {pilot project*} OR {feasibility stud*} OR {program evaluation} OR {health education*} OR {health promotion} OR "treat*" OR {primary prevention} OR "random*" OR {cluster random*} OR trial* OR evaluat* OR effective* OR {mixed methods} OR impact*)) OR ((TITLE-ABS (intervention* OR {weight reduction programs} OR program* OR programme* OR project* OR therapeutics OR scheme OR initiative* OR strateg* OR service* OR {health education} OR {health promotion} OR {primary prevention} OR {health education} OR treat* OR therapy* OR {pilot projects} OR {feasibility studies} OR {program evaluation})) AND (TITLE-ABS (random* OR {cluster random*} OR trial* OR evaluat* OR effective* OR {mixed methods} OR impact* OR {Non-Randomized Controlled Trial*} OR {Pragmatic Clinical Trial} OR {controlled Clinical Trial*} OR {randomized controlled trial*} OR randomized OR randomised OR {clinical trial*} OR randomly OR {controlled study} OR {controlled trial*} OR {control group} OR {intervention group} OR {experimental group} OR {comparison group} OR {control school} OR {intervention school} OR {control community} OR {intervention community} OR {intervention condition} OR {control condition} OR {treatment group} OR {control participant} OR {quasi experimental design} OR {experimental condition} OR {treatment condition} OR {pilot projects} OR {feasibility studies} OR {program evaluation} OR {pre test} OR {post test} OR {quasi experiment*} OR blinded OR {double blind} OR fidelity OR reach OR uptake OR implementation OR {intervention effect*} OR {program effect*} OR {programme effect*} OR {proof of concept*} OR {outcome study}))))) AND (TITLE-ABS ((obesity OR obes* OR {body mass index} OR {BMI} OR {bmi z score} OR {bmi z score} OR {body mass index z-score} OR {body mass index z-score} OR {bmi percentile*} OR {bmi centile*} OR {body mass index percentile*} OR {body mass index centile*} OR {body weight} OR {body size} OR weight* OR waist OR adipos* OR {body composition} OR overweight OR {body weight changes} OR {healthy weight} OR {weight loss} OR {weight gain}))) AND (LIMIT-TO (PUBYEAR , 2023) OR LIMIT-TO (PUBYEAR , 2022) OR LIMIT-TO (PUBYEAR , 2021) OR LIMIT-TO (PUBYEAR , 2020) OR LIMIT-TO (PUBYEAR , 2019) OR LIMIT-TO (PUBYEAR , 2018) OR LIMIT-TO (PUBYEAR , 2017) OR LIMIT-TO (PUBYEAR , 2016) OR LIMIT-TO (PUBYEAR , 2015) OR LIMIT-TO (PUBYEAR , 2014) OR LIMIT-TO (PUBYEAR , 2013) OR LIMIT-TO (PUBYEAR , 2012) OR LIMIT-TO (PUBYEAR , 2011))

PsychINFO

**1**	(((preschool* or pre-school* or pre-school* or kindergar$en or prekinder* or pre-Kinder* or pre Kinder* or headstart or head start or surestart or sure start or family child care home or family childcare home or infant* or toddler* or child care or child-care or childcare or early learning centre or early child* or early care) and education) or ((child* and day care) or daycare or day-care or playgroup* or nurser* or child day care centre*)).mp. [mp=title, abstract, heading word, table of contents, key concepts, original title, tests & measures, mesh]
**2**	(diet* or dietary or food* or snack* or fruit* or drink* or beverage* or juice* or vegetable* or meal* or nutrition* or intake* or consum* or food quality or food preference* or energy density or lifestyle behavio?r or eating behavio?r or healthy eating or feeding behaviour?r or health promotion or eating habit* or food habit* or food choice* or overeat* or over eat* or weight control or menu planning or menu choice or meal choice* or meal* or meal planning or (food* and consum*) or eating habit*).mp. [mp=title, abstract, heading word, table of contents, key concepts, original title, tests & measures, mesh]
**3**	(physical* activ* or playtime or sedentary or exercise* or play* or physical fitness or movement or playtime or sport*).mp. [mp=title, abstract, heading word, table of contents, key concepts, original title, tests & measures, mesh]
**4**	(intervention* or weight reduction program* or program* or programme* or project* or therapeutics or scheme or initiative* or strateg* or life style or service* or pilot project* or feasibility stud* or program evaluation or health education* or health promotion or primary prevention or treat* or random* or cluster random* or trial* or evaluat* or effective* or mixed methods or impact*).mp. [mp=title, abstract, heading word, table of contents, key concepts, original title, tests & measures, mesh]
**5**	(intervention* or weight reduction programs or program*OR programme* or project* or therapeutics or scheme or initiative* or strateg* or service* or health education or health promotion or primary prevention or health education or health promotion or primary prevention or treat* or therapy* or pilot projects or feasibility studies or program evaluation).mp. [mp=title, abstract, heading word, table of contents, key concepts, original title, tests & measures, mesh]
**6**	(random* or cluster random* or trial* or evaluat* or effective* or mixed methods or impact* or Non-Randomized Controlled Trial* or Pragmatic Clinical Trial or controlled Clinical Trial* or randomized controlled trial* or randomized or randomised or clinical trial* or randomly or controlled study or controlled trial* or control group or intervention group or experimental group or comparison group or control school or intervention school or control community or intervention community or intervention condition or control condition or treatment group or control participant or quasi experimental design or experimental condition or treatment condition or pilot projects or feasibility studies or program evaluation or pre test or post test or quasi experiment* or blinded or double blind or fidelity or reach or uptake or implementation or intervention effect* or program effect* or programme effect* or proof of concept* or outcome study).mp. [mp=title, abstract, heading word, table of contents, key concepts, original title, tests & measures, mesh]
**7**	5 and 6
**8**	2 or 3
**9**	4 or 7
**10**	8 and 9
**11**	(obesity or obes* or body mass index or BMI or bmi z score or bmi z score or body mass index z-score or body mass index z-score or bmi percentile* or bmi centile* or body mass index percentile* or body mass index centile* or body weight or body size or weight* or waist or adipos* or body composition or overweight or body weight changes or healthy weight or weight loss or weight gain).mp. [mp=title, abstract, heading word, table of contents, key concepts, original title, tests & measures, mesh]
**12**	1 and 10 and 11

**13**	limit 12 to last 12 years

For peer review only

Social Policy and Practice

**1**	(((preschool* or pre-school* or pre-school* or kindergar$en or prekinder* or pre-Kinder* or pre Kinder* or headstart or head start or surestart or sure start or family child care home or family childcare home or infant* or toddler* or child care or child-care or childcare or early learning centre or early child* or early care) and education) or ((child* and day care) or daycare or day-care or playgroup* or nurser* or child day care centre*)).mp. [mp=abstract, title, publication type, heading word, accession number]
**2**	(diet* or dietary or food* or snack* or fruit* or drink* or beverage* or juice* or vegetable* or meal* or nutrition* or intake* or consum* or food quality or food preference* or energy density or lifestyle behavio?r or eating behavio?r or healthy eating or feeding behaviour?r or health promotion or eating habit* or food habit* or food choice* or overeat* or over eat* or weight control or menu planning or menu choice or meal choice* or meal* or meal planning or (food* and consum*) or eating habit*).mp. [mp=abstract, title, publication type, heading word, accession number]
**3**	(physical* activ* or playtime or sedentary or exercise* or play* or physical fitness or movement or playtime or sport*).mp. [mp=abstract, title, publication type, heading word, accession number]
**4**	(intervention* or weight reduction program* or program* or programme* or project* or therapeutics or scheme or initiative* or strateg* or life style or service* or pilot project* or feasibility stud* or program evaluation or health education* or health promotion or primary prevention or treat* or random* or cluster random* or trial* or evaluat* or effective* or mixed methods or impact*).mp. [mp=abstract, title, publication type, heading word, accession number]
**5**	(intervention* or weight reduction programs or program*OR programme* or project* or therapeutics or scheme or initiative* or strateg* or service* or health education or health promotion or primary prevention or health education or health promotion or primary prevention or treat* or therapy* or pilot projects or feasibility studies or program evaluation).mp. [mp=abstract, title, publication type, heading word, accession number]
**6**	(random* or cluster random* or trial* or evaluat* or effective* or mixed methods or impact* or Non-Randomized Controlled Trial* or Pragmatic Clinical Trial or controlled Clinical Trial* or randomized controlled trial* or randomized or randomised or clinical trial* or randomly or controlled study or controlled trial* or control group or intervention group or experimental group or comparison group or control school or intervention school or control community or intervention community or intervention condition or control condition or treatment group or control participant or quasi experimental design or experimental condition or treatment condition or pilot projects or feasibility studies or program evaluation or pre test or post test or quasi experiment* or blinded or double blind or fidelity or reach or uptake or implementation or intervention effect* or program effect* or programme effect* or proof of concept* or outcome study).mp. [mp=abstract, title, publication type, heading word, accession number]
**7**	5 and 6
**8**	2 or 3
**9**	4 or 7
**10**	8 and 9
**11**	(obesity or obes* or body mass index or BMI or bmi z score or bmi z score or body mass index z-score or body mass index z-score or bmi percentile* or bmi centile* or body mass index percentile* or body mass index centile* or body weight or body size or weight* or waist or adipos* or body composition or overweight or body weight changes or healthy weight or weight loss or weight gain).mp. [mp=abstract, title, publication type, heading word, accession number]
**12**	1 and 10 and 11
**13**	limit 12 to last 12 years

For peer review only

HMIC Health Management Information Consortium

**1**	(((preschool* or pre-school* or pre-school* or kindergar$en or prekinder* or pre-Kinder* or pre Kinder* or headstart or head start or surestart or sure start or family child care home or family childcare home or infant* or toddler* or child care or child-care or childcare or early learning centre or early child* or early care) and education) or ((child* and day care) or daycare or day-care or playgroup* or nurser* or child day care centre*)).mp. [mp=title, other title, abstract, heading words]
**2**	(diet* or dietary or food* or snack* or fruit* or drink* or beverage* or juice* or vegetable* or meal* or nutrition* or intake* or consum* or food quality or food preference* or energy density or lifestyle behavio?r or eating behavio?r or healthy eating or feeding behaviour?r or health promotion or eating habit* or food habit* or food choice* or overeat* or over eat* or weight control or menu planning or menu choice or meal choice* or meal* or meal planning or (food* and consum*) or eating habit*).mp. [mp=title, other title, abstract, heading words]
**3**	(physical* activ* or playtime or sedentary or exercise* or play* or physical fitness or movement or playtime or sport*).mp. [mp=title, other title, abstract, heading words]
**4**	(intervention* or weight reduction program* or program* or programme* or project* or therapeutics or scheme or initiative* or strateg* or life style or service* or pilot project* or feasibility stud* or program evaluation or health education* or health promotion or primary prevention or treat* or random* or cluster random* or trial* or evaluat* or effective* or mixed methods or impact*).mp. [mp=title, other title, abstract, heading words]
**5**	(intervention* or weight reduction programs or program*OR programme* or project* or therapeutics or scheme or initiative* or strateg* or service* or health education or health promotion or primary prevention or health education or health promotion or primary prevention or treat* or therapy* or pilot projects or feasibility studies or program evaluation).mp. [mp=title, other title, abstract, heading words]
**6**	(random* or cluster random* or trial* or evaluat* or effective* or mixed methods or impact* or Non-Randomized Controlled Trial* or Pragmatic Clinical Trial or controlled Clinical Trial* or randomized controlled trial* or randomized or randomised or clinical trial* or randomly or controlled study or controlled trial* or control group or intervention group or experimental group or comparison group or control school or intervention school or control community or intervention community or intervention condition or control condition or treatment group or control participant or quasi experimental design or experimental condition or treatment condition or pilot projects or feasibility studies or program evaluation or pre test or post test or quasi experiment* or blinded or double blind or fidelity or reach or uptake or implementation or intervention effect* or program effect* or programme effect* or proof of concept* or outcome study).mp. [mp=title, other title, abstract, heading words]
**7**	5 and 6
**8**	2 or 3
**9**	4 or 7
**10**	8 and 9
**11**	(obesity or obes* or body mass index or BMI or bmi z score or bmi z score or body mass index z-score or body mass index z-score or bmi percentile* or bmi centile* or body mass index percentile* or body mass index centile* or body weight or body size or weight* or waist or adipos* or body composition or overweight or body weight changes or healthy weight or weight loss or weight gain).mp. [mp=title, other title, abstract, heading words]
**12**	1 and 10 and 11
**13**	limit 12 to last 12 years

For peer review only

Trials Register of Promoting Health Interventions (TRoPHI)

saved 551 records from the following searches:

Search 1:

2343: 2341 AND 2342 361 records (of which saved records from year 2011 onwards)

2342: Coded with: children (0-10 yrs) 1933

2341: Coded with: obesity 1194

Search 2:

2366: 2365 AND 2329 (unscreened records): 123 records

2365: 2364 AND 2347 1116

2347: "toddler* OR young child* OR preschool* OR infant* OR child*" (in Title and Abstract)

2364: Coded with: Weight Loss, OR Physical Activity

Search 3:

2344: AND 2347 AND 2355 AND 2338: 221 records

2344: Not coded with: EPPI-Centre Health promotion keywording strategy

2347: "toddler* OR young child* OR preschool* OR infant* OR child*" (in Title and Abstract)

2355: "obes* OR sedentar* OR physical* OR diet* OR exercise OR BMI OR "body weight" OR "body size" OR waist " (in Title and Abstract)

2338: Coded with: Include: Randomised controlled trial, OR Include: Non-Random 14391

Search 4: 136 records: (of which saved records from year 2010 onwards)

"obes* OR sedentar* OR physical* OR diet*" (in Title and Abstract)

AND

"exercise OR BMI OR "body weight" OR "body size" OR waist " (in Title and Abstract)

AND

"toddler* OR young child* OR preschool* OR infant* OR child*" (in Title and Abstract)

AND

Not coded with: EPPI-Centre Health promotion keywording strategy

AND

Not coded with: Ex 1: Not Health Promotion or Public Health, OR Ex 2: Not Human or Environment, OR Ex 3: NOT an evaluation of an intervention, OR Ex: 4 No control or comparison group, OR Ex 5: Item is a review or meta-analysis, OR MARKER: QUERY, OR Duplicate

For peer review only

Appendix 2

Depiction of the screening process

The graph below depicts the screening process on tittle and abstract. The prioritisation of records was refreshing regularly. The most relevant articles were screened first. The screening stopped when the graph reached a plateau.

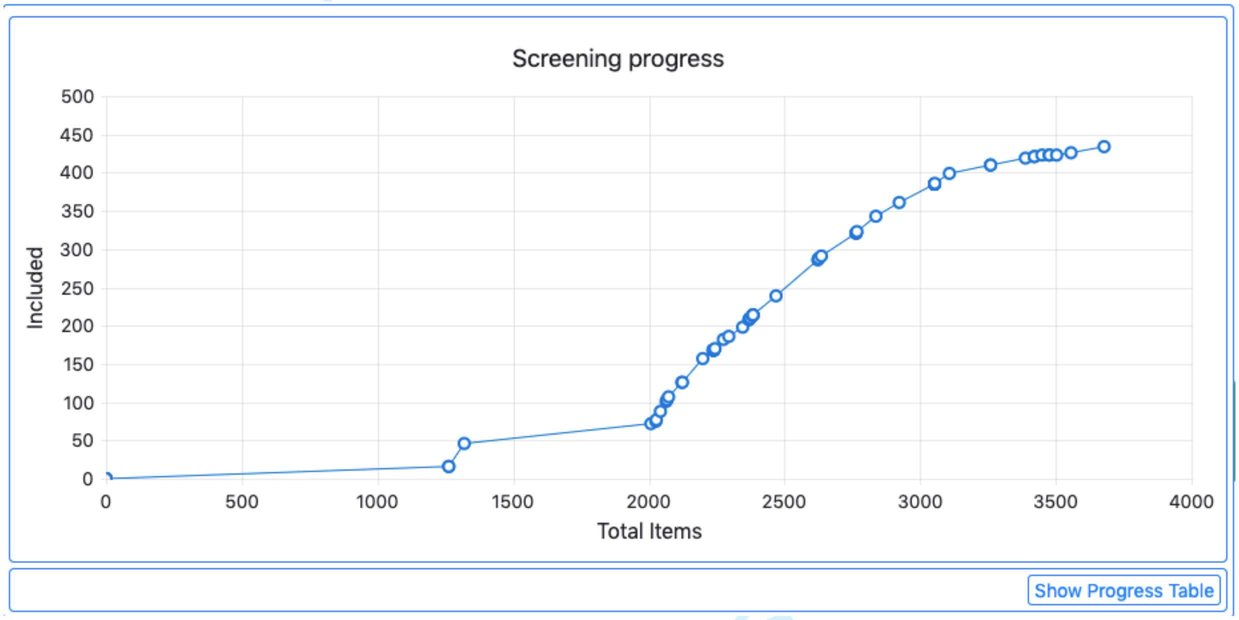

Appendix 3

Bias assessment

Table S2: Studies assessed using the ROB tool

	Randomisation bias	Adhering to the intervention	Missing data	Outcome measurement	Reporting bias	Overall
Hodgkinson 2019	High	High	Low	Low	Low	High
Malden 2019	Moderate	High	Low	Low	Low	High
Bryant 2021	Moderate	High	Low	Low	Low	High
Lanigan (unpublished)	Low	Low	High	Low	High	High

High risk of bias

Moderate risk of bias

Low risk of bias

Table S3: Studies assessed using the ROBINS-I tool

	Confounding bias	Selection of participants bias	Classification of interventions bias	Adherence to the intervention	Missing data	Outcome measurement	Reporting bias	Overall
Lee 2021	Low	Moderate	Low	Moderate	High	Low	Low	High

For peer review only

PRISMA 2020 Checklist

Section and Topic	Item #	Checklist item	Location where item is reported
**TITLE**			
Title	1	Identify the report as a systematic review.	Lines 1-2
**ABSTRACT**			
Abstract	2	See the PRISMA 2020 for Abstracts checklist.	Lines 16-40
**INTRODUCTION**			
Rationale	3	Describe the rationale for the review in the context of existing knowledge.	Lines 93-100
Objectives	4	Provide an explicit statement of the objective(s) or question(s) the review addresses.	Lines 72-79
**METHODS**			
Eligibility criteria	5	Specify the inclusion and exclusion criteria for the review and how studies were grouped for the syntheses.	Lines 115-128 & Lines 152-155
Information sources	6	Specify all databases, registers, websites, organisations, reference lists and other sources searched or consulted to identify studies. Specify the date when each source was last searched or consulted.	Lines 106-114 & Appendix 1
Search strategy	7	Present the full search strategies for all databases, registers and websites, including any filters and limits used.	Appendix 1
Selection process	8	Specify the methods used to decide whether a study met the inclusion criteria of the review, including how many reviewers screened each record and each report retrieved, whether they worked independently, and if applicable, details of automation tools used in the process.	Lines 129-147
Data collection process	9	Specify the methods used to collect data from reports, including how many reviewers collected data from each report, whether they worked independently, any processes for obtaining or confirming data from study investigators, and if applicable, details of automation tools used in the process.	Lines 141-147
Data items	10a	List and define all outcomes for which data were sought. Specify whether all results that were compatible with each outcome domain in each study were sought (e.g. for all measures, time points, analyses), and if not, the methods used to decide which results to collect.	Lines 155-162
	10b	List and define all other variables for which data were sought (e.g. participant and intervention characteristics, funding sources). Describe any assumptions made about any missing or unclear information.	Lines 142-147
Study risk of bias assessment	11	Specify the methods used to assess risk of bias in the included studies, including details of the tool(s) used, how many reviewers assessed each study and whether they worked independently, and if applicable, details of automation tools used in the process.	Lines 163-167
Effect measures	12	Specify for each outcome the effect measure(s) (e.g. risk ratio, mean difference) used in the synthesis or presentation of results.	Lines 155-157
Synthesis methods	13a	Describe the processes used to decide which studies were eligible for each synthesis (e.g. tabulating the study intervention characteristics and comparing against the planned groups for each synthesis (item #5)).	Lines 152-157
	13b	Describe any methods required to prepare the data for presentation or synthesis, such as handling of missing summary statistics, or data conversions.	NA
	13c	Describe any methods used to tabulate or visually display results of individual studies and syntheses.	NA
	13d	Describe any methods used to synthesize results and provide a rationale for the choice(s). If meta-analysis was performed, describe the model(s), method(s) to identify the presence and extent of statistical heterogeneity, and software package(s) used.	Lines 152-155
	13e	Describe any methods used to explore possible causes of heterogeneity among study results (e.g. subgroup analysis, meta-regression).	NA
	13f	Describe any sensitivity analyses conducted to assess robustness of the synthesized results.	NA
Reporting bias assessment	14	Describe any methods used to assess risk of bias due to missing results in a synthesis (arising from reporting biases).	NA

For peer review only - http://bmjopen.bmj.com/site/about/guidelines.xhtml

PRISMA 2020 Checklist

Section and Topic	Item #	Checklist item	Location where item is reported
Certainty assessment	15	Describe any methods used to assess certainty (or confidence) in the body of evidence for an outcome.	NA
**RESULTS**			
Study selection	16a	Describe the results of the search and selection process, from the number of records identified in the search to the number of studies included in the review, ideally using a flow diagram.	Lines 177-188 & Figure 1
	16b	Cite studies that might appear to meet the inclusion criteria, but which were excluded, and explain why they were excluded.	Figure 1
Study characteristics	17	Cite each included study and present its characteristics.	Lines 187-200 & Table 1
Risk of bias in studies	18	Present assessments of risk of bias for each included study.	Appendix 3
Results of individual studies	19	For all outcomes, present, for each study: (a) summary statistics for each group (where appropriate) and (b) an effect estimate and its precision (e.g. confidence/credible interval), ideally using structured tables or plots.	Table 1
Results of syntheses	20a	For each synthesis, briefly summarise the characteristics and risk of bias among contributing studies.	Lines 202-258
	20b	Present results of all statistical syntheses conducted. If meta-analysis was done, present for each the summary estimate and its precision (e.g. confidence/credible interval) and measures of statistical heterogeneity. If comparing groups, describe the direction of the effect.	NA
	20c	Present results of all investigations of possible causes of heterogeneity among study results.	NA
	20d	Present results of all sensitivity analyses conducted to assess the robustness of the synthesized results.	NA
Reporting biases	21	Present assessments of risk of bias due to missing results (arising from reporting biases) for each synthesis assessed.	NA
Certainty of evidence	22	Present assessments of certainty (or confidence) in the body of evidence for each outcome assessed.	NA
**DISCUSSION**			
Discussion	23a	Provide a general interpretation of the results in the context of other evidence.	Lines 270-277
	23b	Discuss any limitations of the evidence included in the review.	Lines 282-288 & Lines 311-319
	23c	Discuss any limitations of the review processes used.	Lines 337-344
	23d	Discuss implications of the results for practice, policy, and future research.	Lines 345-353
**OTHER INFORMATION**			
Registration and protocol	24a	Provide registration information for the review, including register name and registration number, or state that the review was not registered.	Lines 102-105
	24b	Indicate where the review protocol can be accessed, or state that a protocol was not prepared.	Lines 102-105
	24c	Describe and explain any amendments to information provided at registration or in the protocol.	NA
Support	25	Describe sources of financial or non-financial support for the review, and the role of the funders or sponsors in the review.	Lines 50-55
Competing interests	26	Declare any competing interests of review authors.	Line 56
Availability of data, code and	27	Report which of the following are publicly available and where they can be found: template data collection forms; data extracted from included studies; data used for all analyses; analytic code; any other materials used in the review.	NA

For peer review only - http://bmjopen.bmj.com/site/about/guidelines.xhtml

PRISMA 2020 Checklist

Section and Topic	Item #	Checklist item	Location where item is reported
other materials			

From: Page MJ, McKenzie JE, Bossuyt PM, Boutron I, Hoffmann TC, Mulrow CD, et al. The PRISMA 2020 statement: an updated guideline for reporting systematic reviews. BMJ 2021;372:n71. doi: 10.1136/bmj.n71

For more information, visit: http://www.prisma-statement.org/

For peer review only
